# Can synthetic data reproduce real-world findings in epidemiology? A replication study using adversarial random forests

**DOI:** 10.1093/ije/dyag164

**Published:** 2026-08-29

**Authors:** Jan Kapar, Kathrin Günther, Lori Ann Vallis, Klaus Berger, Nadine Binder, Hermann Brenner, Stefanie Castell, Beate Fischer, Volker Harth, Bernd Holleczek, Timm Intemann, Till Ittermann, André Karch, Thomas Keil, Lilian Krist, Berit Lange, Michael F Leitzmann, Katharina Nimptsch, Nadia Obi, Iris Pigeot, Tobias Pischon, Tamara Schikowski, Börge Schmidt, Carsten Oliver Schmidt, Anja M Sedlmair, Justine Tanoey, Harm Wienbergen, Andreas Wienke, Claudia Wigmann, Marvin N Wright

**Affiliations:** Leibniz Institute for Prevention Research and Epidemiology—BIPS, Bremen, Germany; Faculty of Mathematics and Computer Science, University of Bremen, Bremen, Germany; Department of Human Health Sciences, University of Guelph, Guelph, Ontario, Canada; Leibniz Institute for Prevention Research and Epidemiology—BIPS, Bremen, Germany; Department of Human Health Sciences, University of Guelph, Guelph, Ontario, Canada; Institute of Epidemiology and Social Medicine, University of Münster, Münster, Germany; Institute of General Practice/Family Medicine, Faculty of Medicine and Medical Center, University of Freiburg, Freiburg, Germany; Freiburg Center for Data Analysis, Modeling and AI, University of Freiburg, Freiburg, Germany; Cancer Prevention Graduate School, German Cancer Research Center (DKFZ), Heidelberg, Germany; Department of Epidemiology, Helmholtz Centre for Infection Research (HZI), Brunswick, Germany; Department of Epidemiology and Preventive Medicine, University of Regensburg, Regensburg, Germany; Institute for Occupational and Maritime Medicine (ZfAM), University Medical Center Hamburg-Eppendorf, Hamburg, Germany; Saarland Cancer Registry, Saarbrücken, Germany; Leibniz Institute for Prevention Research and Epidemiology—BIPS, Bremen, Germany; Institute for Community Medicine, University Medicine Greifswald, Greifswald, Germany; Institute of Epidemiology and Social Medicine, University of Münster, Münster, Germany; Institute of Social Medicine, Epidemiology and Health Economics, Charité Universitätsmedizin Berlin, Berlin, Germany; Institute of Clinical Epidemiology and Biometry, University of Würzburg, Würzburg, Germany; State Institute of Health I, Bavarian Health and Food Safety Authority, Erlangen, Germany; Institute of Social Medicine, Epidemiology and Health Economics, Charité Universitätsmedizin Berlin, Berlin, Germany; Department of Epidemiology, Helmholtz Centre for Infection Research (HZI), Brunswick, Germany; Department of Epidemiology and Preventive Medicine, University of Regensburg, Regensburg, Germany; Molecular Epidemiology Research Group, Max Delbrück Center for Molecular Medicine in the Helmholtz Association (MDC), Berlin, Germany; Institute for Occupational and Maritime Medicine (ZfAM), University Medical Center Hamburg-Eppendorf, Hamburg, Germany; Leibniz Institute for Prevention Research and Epidemiology—BIPS, Bremen, Germany; Faculty of Mathematics and Computer Science, University of Bremen, Bremen, Germany; Molecular Epidemiology Research Group, Max Delbrück Center for Molecular Medicine in the Helmholtz Association (MDC), Berlin, Germany; Biobank Technology Platform, Max Delbrück Center for Molecular Medicine in the Helmholtz Association (MDC), Berlin, Germany; Charité – Universitätsmedizin Berlin, corporate member of Freie Universität Berlin and Humboldt-Universität zu Berlin, Berlin, Germany; IUF—Leibniz Research Institute for Environmental Medicine, Düsseldorf, Germany; Department of Environment and Health, School of Public Health, University of Bielefeld, Bielefeld, Germany; Institute for Medical Informatics, Biometry and Epidemiology, University Hospital of Essen, University of Duisburg, Essen, Germany; Institute for Community Medicine, University Medicine Greifswald, Greifswald, Germany; Department of Epidemiology and Preventive Medicine, University of Regensburg, Regensburg, Germany; Center for Translational Oncology, University Hospital Regensburg, Regensburg, Germany; Bavarian Cancer Research Center (BZKF), Regensburg, Germany; Leibniz Institute for Prevention Research and Epidemiology—BIPS, Bremen, Germany; Bremen Institute for Heart and Circulation Research (BIHKF), Bremen, Germany; Institute of Medical Epidemiology, Biometry, and Informatics, Profile Center Health Sciences, Faculty of Medicine, Martin Luther University Halle-Wittenberg, Halle, Germany; IUF—Leibniz Research Institute for Environmental Medicine, Düsseldorf, Germany; Leibniz Institute for Prevention Research and Epidemiology—BIPS, Bremen, Germany; Faculty of Mathematics and Computer Science, University of Bremen, Bremen, Germany; Department of Public Health, University of Copenhagen, Copenhagen, Denmark

**Keywords:** generative artificial intelligence, generative machine learning, adversarial random forests, NAKO German National Cohort, Guelph Family Health Study, synthetic data

## Abstract

**Background:**

Synthetic data hold substantial potential to address practical challenges in epidemiology due to restricted data access and privacy concerns. However, many current methods suffer from limited quality, high computational demands, and complexity for non-experts. Furthermore, common evaluation strategies for synthetic data often fail to directly reflect statistical utility and measure privacy risks sufficiently. Against this background, a critical underexplored question is whether synthetic data can reliably reproduce key findings from epidemiological research while preserving privacy.

**Methods:**

We propose adversarial random forests (ARF) as an efficient and convenient method for synthesizing tabular epidemiological data. To evaluate its performance, we replicated statistical analyses from six epidemiological publications covering blood pressure, anthropometry, myocardial infarction, accelerometry, loneliness, and diabetes, from the German National Cohort (NAKO Gesundheitsstudie), the Bremen STEMI Registry U45 Study, and the Guelph Family Health Study. We further assessed how dataset dimensionality and variable complexity affect the quality of synthetic data, and contextualized ARF’s performance by comparison with commonly used tabular data synthesizers in terms of utility, privacy, generalization, and runtime.

**Results:**

Across all replicated studies, results on ARF-generated synthetic data consistently aligned with original findings. Even for datasets with relatively low sample size-to-dimensionality ratios, replication outcomes closely matched the original results across descriptive and inferential analyses. Reduced dimensionality and variable complexity further enhanced synthesis quality. ARF demonstrated favourable performance regarding utility, privacy preservation, and generalization relative to other synthesizers and superior computational efficiency.

**Conclusions:**

In summary, ARF reliably generates high-quality synthetic data that replicate diverse epidemiological analyses while offering a competitive privacy–utility trade-off.

Key Messages

- We systematically evaluated synthetic epidemiological data generated with adversarial random forests (ARF), assessing reproducibility of key study findings as well as privacy preservation, generalization to unseen data, and computational efficiency.
- ARF-generated synthetic data consistently replicated original results, demonstrating a superior or matching utility-privacy trade-off in comparison to alternative methods in the majority of experiments while offering considerably greater computational efficiency.
- These findings confirm that ARF is a valuable and versatile tool for generating high-quality synthetic data in diverse epidemiological research contexts.

## Introduction

Researchers in epidemiology often encounter specific challenges before conducting statistical analyses: health-related data are highly protected by privacy laws, such as Health Insurance Portability and Accountability [1] in the United States and General Data Protection Regulation [2] in the European Union, and access requests can take several months from the initial application to final data access.

Generative artificial intelligence (AI) [3] famously applied to text and image data (e.g. ChatGPT [4], DALL-E [5]), could help address these obstacles by producing realistic, privacy-preserving synthetic health data for sharing, previewing, or substituting original data.

Despite successes in medical image synthesis [6, 7], generating and evaluating high-quality tabular health data—rows as individual observations, columns as variables—remains challenging [8]. Existing approaches are still developing, no gold-standard method exists, and many new methods are not yet directly applicable in epidemiology, as they often lack support for missing values and are not available in well-maintained packages. With only a few exceptions [9, 10], most generative methods are evaluated on standard ML datasets. Benchmarks typically rely on metrics that are difficult to interpret in applied research and rarely or insufficiently assess privacy [11–13], which is essential in epidemiological applications.

We propose adversarial random forests (ARF) [14], a generative AI method specifically designed for tabular data. ARF is available as an R package [15], easy to use for researchers with limited machine learning experience, computationally efficient, and competitive in performance. We demonstrate its utility in real-world epidemiological settings by replicating analyses from six studies [16–21] based on the German National Cohort (NAKO) [22], the Bremen STEMI Registry U45 Study (BSR-U45) [18], and the Guelph Family Health Study (GFHS) [19, 23] using ARF-generated synthetic data. Moreover, we examine how dataset dimensionality and variable complexity affect the quality and stability of synthetic data to assess how ARF performs across diverse scenarios. In addition, we perform experiments to systematically evaluate privacy preservation, generalization to out-of-sample data, and hyperparameter effects. We further contextualize ARF’s performance by comparing it with commonly used tabular data synthesizers [11, 24–26] along these aspects of synthetic data quality as well as in terms of runtime. This comprehensive assessment positions ARF as a valuable approach for generating high-quality synthetic epidemiological data while providing insight into its strengths, limitations, and applicability in real-world research contexts.

## Data and methods

The selected datasets, along with the synthesis and evaluation approach, were chosen with a focus on their applicability to epidemiological research.

### Data

We selected the included data sources and original studies with an emphasis on variety regarding study subject, data origin, size and dimensionality, and the complexity of statistical analyses. This way, we cover different challenging scenarios for successful data synthesis, such as small data size, high dimensionality, mixed variable types, and irregular distribution shapes.

#### Data sources

The original studies chosen for this replication analysis were based on NAKO, BSR-U45, and GFHS data. The NAKO is Germany’s largest cohort study, following over 200 000 participants from age 19 to 74 over the long term. It encompasses socioeconomic, demographic, genetic and lifestyle information as well as medical history and examination data in order to investigate the development of major diseases, such as cancer, cardiovascular conditions, diabetes, and mental health disorders [22]. The BSR-U45 is a specialized substudy within the Bremen Heart Registry that focuses on patients aged 45 or younger who experienced myocardial infarction, combining retrospective and prospective data on demographic, lifestyle, clinical, and laboratory factors to investigate early-onset MI risk [18]. The GFHS is a long-term cohort study that involves over 246 families in Wellington County, Ontario, Canada. It aims to identify early life risk factors for obesity and chronic diseases and help families establish healthy routines related to nutrition, physical activity, sleep, and screen time [23].

#### Original studies

Five of the six selected original studies were based on NAKO data [16–18, 20, 21], one of which also used BSR-U45 data [18], and one was based on GFHS data [19]. Table 1 summarizes all datasets used for these studies, including data source, size, dimensionality, variable types, and missing values. Schikowski *et al.* [16] examined methodological differences in blood pressure measurement, with NAKO conducting two readings compared to three in other German population studies. Age- and sex-specific mean blood pressure values and hypertension frequencies were compared. Fischer *et al.* [17] presented descriptive analyses of anthropometric measures, comparing overweight and obesity frequencies alongside mean adipose tissue thickness by sex and age. In a case-control study, Wienbergen *et al.* [18] used logistic regression to determine associations of lifestyle and metabolic factors with early-onset myocardial infarction risk. Breau *et al.* [19] examined the effect of movement intensity cutpoints for accelerometer data of young children on physical activity metrics. Berger *et al.* [20] analysed loneliness frequency and its association with depression and anxiety during the first SARS-CoV-2 wave, using linear regression to identify factors influencing loneliness. In Tanoey *et al.* [21], the relationships between birth order, delivery mode, and daycare attendance with the risk of type 1 diabetes were examined and stratified by childhood- and adult-onset type 1 diabetes using Cox regression.

**Table 1:** Overview of publications and data used for synthetic data replications.

Publication	Data source	Research topic	*n*	*d* (num/cat/lvls)	*n*_NA_	*d*_NA_	%_NA_
Schikowski *et al.* [16]	NAKO	Blood pressure	98 332	7 (5/2/4)	0	0	0
Fischer *et al.* [17]	NAKO	Anthropometry	101 557	12 (10/2/20)	97 903	9	34.43
Wienbergen *et al.* [18]	NAKO, BSR-U45	Myocardial infarction	1713	12 (3/9/24)	176	1	0.86
Breau *et al.* [19]	GFHS	Accelerometry	262	28 (25/3/34)	16	2	0.23
Berger *et al.* [20]	NAKO	Loneliness/COVID-19	113 527	47 (3/44/230)	0	0	0
Tanoey *et al.* [21]	NAKO	Type 1 diabetes	101 570	19 (7/12/42)	100 621	6	12.66

The values for *n* partially deviate from the numbers given in the original publications due to participants withdrawing their consent for data usage in the respective cohort studies over time. *n*, number of rows/participants; *d*, dimensionality; num, number of numeric variables; cat, number of categorical variables; lvls, total number of categories (i.e. levels) across all categorical variables; *n*_NA_, number of rows containing missing values; *d*_NA_, number of variables containing missing values; %_NA_, percentage of missing values; NAKO, German National Cohort (NAKO Gesundheitsstudie); BSR-U45, Bremen STEMI Registry U45 Study; GFHS, Guelph Family Health Study.

### Methods

The generative AI method ARF was used for data synthesis. The statistical analyses of the included studies were replicated using synthetic data, and their results then compared to the original findings to assess the statistical utility of the synthetic data.

#### Tree-based generative artificial intelligence for tabular data: adversarial random forests

Generative AI is concerned with modelling the underlying joint distribution of given data in order to generate realistic synthetic samples [3]. Deep learning approaches dominate this discipline, especially for image and text data. Tabular data, however, present unique challenges, such as mixed variable types (e.g. continuous or categorical), a lack of natural positional ordering of variables, and irregular distributions, all of which substantially complicate the learning task [8, 27, 28]. Recent advances of generative deep learning methods for tabular data often come at increased computational cost and demanding hardware requirements, and using these methods is often non-trivial for non-experts [28].

Tree-based methods [29] are a well-suited and less resource-demanding alternative to deep learning for tabular learning tasks [12, 30, 31]. The tree-based method ARF is competitive to state-of-the-art deep learning models for tabular data synthesis while often operating orders of magnitude faster and requiring no GPU calculations [12, 14]. Synthetic data can be created with one line of R code: synthetic_data <- rarf(original_data).

ARF utilizes random forests (RFs) [32] as its foundation. It iteratively learns data dependencies starting with an RF classification, where the original dataset is distinguished from a constructed version built from original entries with permuted, and thus independent, variables. Hereby, a partitioning of the data manifold is determined in which original and constructed entries are indistinguishable for the RF. This is utilized to assume variable independence for the original data locally and perform variable-wise univariate density estimation within the partitioning units, which are then combined in a global mixture distribution [33]. ARF generates data by drawing samples from this estimated mixture [14]. It further supports joint and conditional density estimation, training and synthesis with missing data, and conditional sampling, which enables applications such as what-if analyses, missing data imputation, and data balancing [34–36].

#### Replication and downstream utility evaluation procedure

The replication and evaluation procedure are split into three main parts: original dataset preparation before synthesis, generating synthetic data with ARF, and performing the original statistical analyses both on original and synthetic data to compare the results.

##### Original dataset preparation

Data cleaning, missing data handling, and variable derivations followed the original authors’ protocols where available. To show the effects of dimensionality and complexity induced by derived variables, a dataset with a task-specific variable subset was prepared for selected analyses. We refer to the corresponding synthesis approaches as *general synthesis* and *task-specific synthesis*, respectively: for general synthesis, all variables present in the original dataset were included without derivations; for task-specific synthesis, only the variable subset used for the analysis was included, with derived variables replacing original ones when applicable.

##### Synthesis

Data synthesis with ARF contains two sources of randomness: model training and data sampling. To account for that, we trained 100 ARF models for each original dataset and sampled 20 times from each model, resulting in a total of 2000 synthetic datasets per original dataset.

##### Evaluation

For each statistical result of the original studies, we reported the estimate together with a 95% bootstrap percentile confidence interval (CI) [37] based on 2000 bootstrap resamples to quantify the uncertainty of the original analyses. Across the 2000 synthetic datasets, we reported the median estimate and the corresponding empirical 95% percentile CI. To quantify deviations between original and synthetic results across all reported statistics within each analysis table or plot, we additionally reported summary metrics, including the mean absolute standardized difference (MASD) and the mean confidence interval overlap (CIO) [38]. Further details on the evaluation procedure and the employed metrics for quantification are provided in Supplement A, in the supplementary material.

##### Additional evaluation of privacy, generalization, and comparative performance

While the main evaluation focuses on the reproducibility of published analyses using ARF-generated synthetic data, it does not assess other important aspects of synthetic data quality, such as privacy risk and generalization to unseen data. In practice, privacy preservation and utility typically form a trade-off [39]. We therefore conducted additional experiments evaluating these aspects.

To contextualize ARF’s performance, we further compared it with several commonly used tabular data synthesizers: synthpop [24], Bayesian networks [25], PrivBayes [26], CTGAN [11], and TVAE [11]. As the minimum node size in ARF controls the granularity of tree partitions and thereby affects the utility–privacy trade-off, we additionally evaluated performance across different values of this hyperparameter. Further methodological details are provided in Supplement C, in the supplementary material.

## Results

We first present general synthesis results to assess the utility of ARF-generated data across datasets and analyses, followed by the evaluation of task-specific synthesis, highlighting the effects of dimensionality and derived variables. The complete set of replication results for all analyses in the original publications including numerical tables for figures is available in the Supplement B, in the supplementary material. The section concludes with a summary of the additional evaluation results regarding privacy, generalization, and runtime.

### Downstream utility (general synthesis)

#### *Replication results for Schikowski et al.* [16]

**Figure 1:**
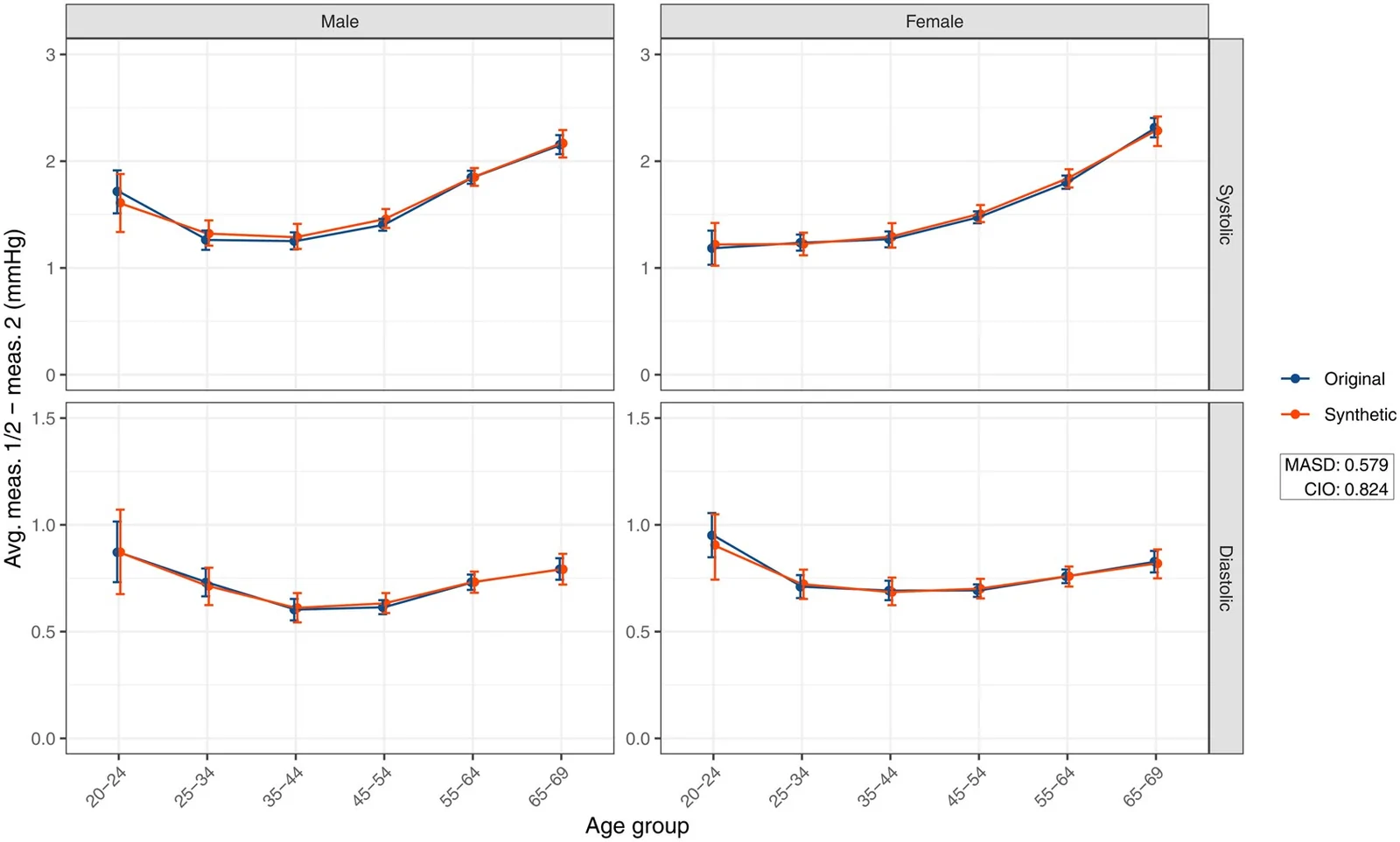
Replication of Figure 2 in Schikowski *et al*. [16]: differences of mean blood pressure values (in mmHg) using the mean of first and second measurement or the second measurement only by sex and age group (in years). Percentile-based 95% bootstrap confidence intervals are reported for original data results. Median and percentile-based 95% confidence intervals of synthetic data results are reported. avg., average; meas., measurement; MASD, mean absolute standardized difference; CIO, mean confidence interval overlap.

The dataset for Schikowski *et al.* [16] contained 98 332 participants, seven variables, and no missing values. Figure 1 (replicating Figure 2 of Schikowski *et al.* [16]) shows the interplay of six of these variables, following derivations as in the original study: for systolic and diastolic blood pressure, the difference between using the mean of the first and second measurements versus only the second measurement was calculated. All median values of the synthetic estimates closely resembled those from the original data, while the 95% CIs were consistently well-aligned (MASD = 0.85; CIO = 0.823). Similar conclusions were drawn for the remaining analyses of this publication (see supplementary material for Supplement B.1). Given the strong similarity of results, all conclusions in Schikowski *et al.* [16] drawn from original data were equally supported by ARF-generated data.

#### *Replication results for Fischer et al.* [17]

**Figure 2:**
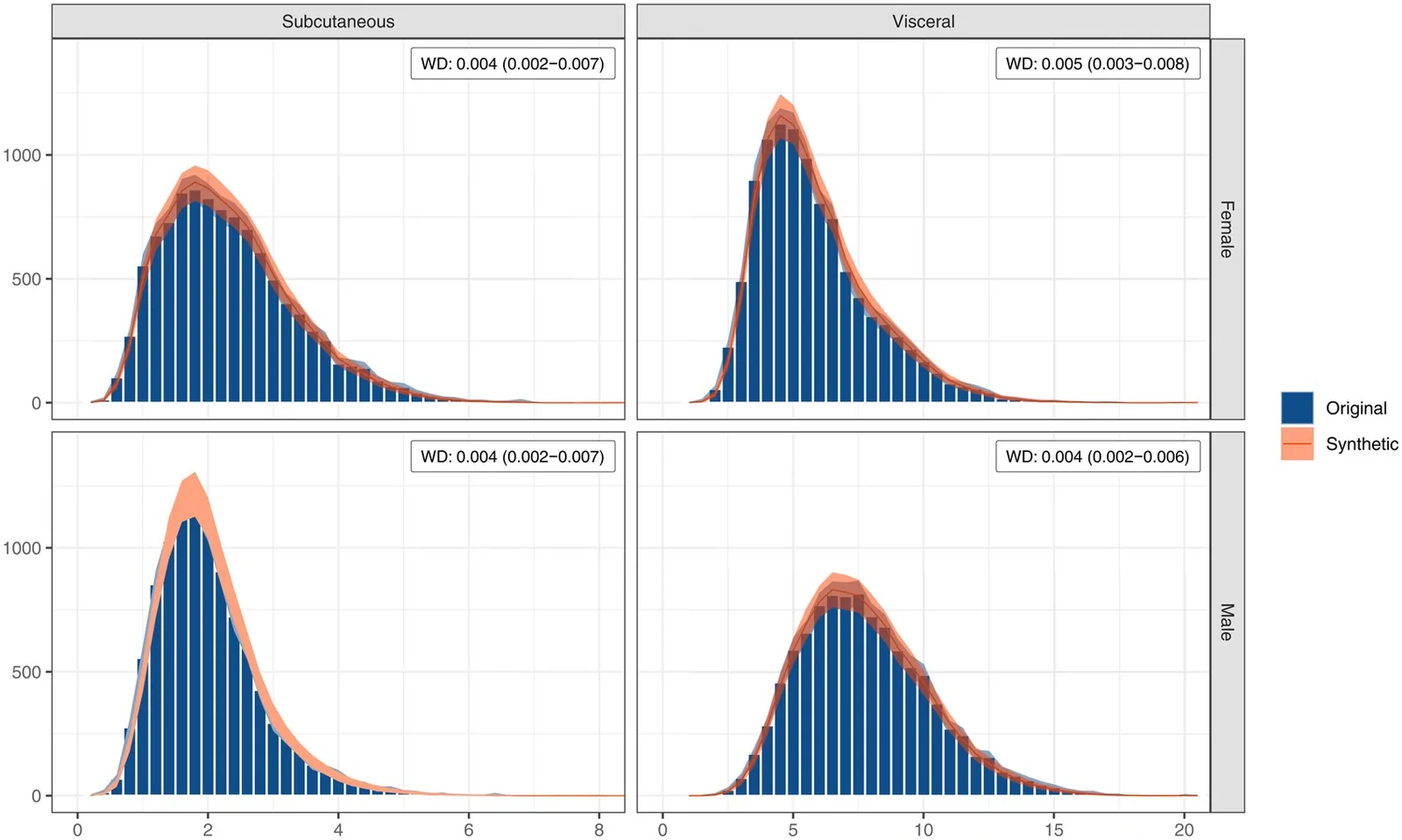
Replication of Figure 5 in Fischer *et al*. [17]: subcutaneous and visceral abdominal adipose tissue thickness by sex. Percentile-based 95% bootstrap confidence intervals are reported for original data results. Median and percentile-based 95% confidence intervals of synthetic data results are reported. In order to provide a clear comparability of the distributions despite the variability in the distribution of missing values in the synthetic data, the counts for the synthetic results were rescaled to match the total count of the original ones; WD, Wasserstein distance.

With 101 557 participants and 12 variables, the data for Fischer *et al.* [17] were of similar size and dimensionality as those from Schikowski *et al.* [16] but contained over one-third of missing values. The histograms shown in Fig. 2 (replicating Figure 5 in Fischer *et al.* [17]) were calculated using five variables. The sex-specific means of both measurements for subcutaneous and visceral adipose tissue thickness were calculated, with 80% missingness reflecting NAKO’s scan coverage plan. For comparability between original and synthetic distributions, the synthetic results were rescaled sex-independently to match the total count of non-missing original values. This accounts for variability in the distribution of missing values in the synthetic data. Despite the high missingness rate, the synthetic data histograms recreated the original ones closely, which is reflected by low Wasserstein distance [40] values (WD ≤ 0.005 for all subgroups). Also, the further synthetic results for this study (see supplementary material for Supplement B.2) consistently mirrored the original outcomes.

#### *Replication results for Wienbergen et al.* [18]

**Figure 3:**
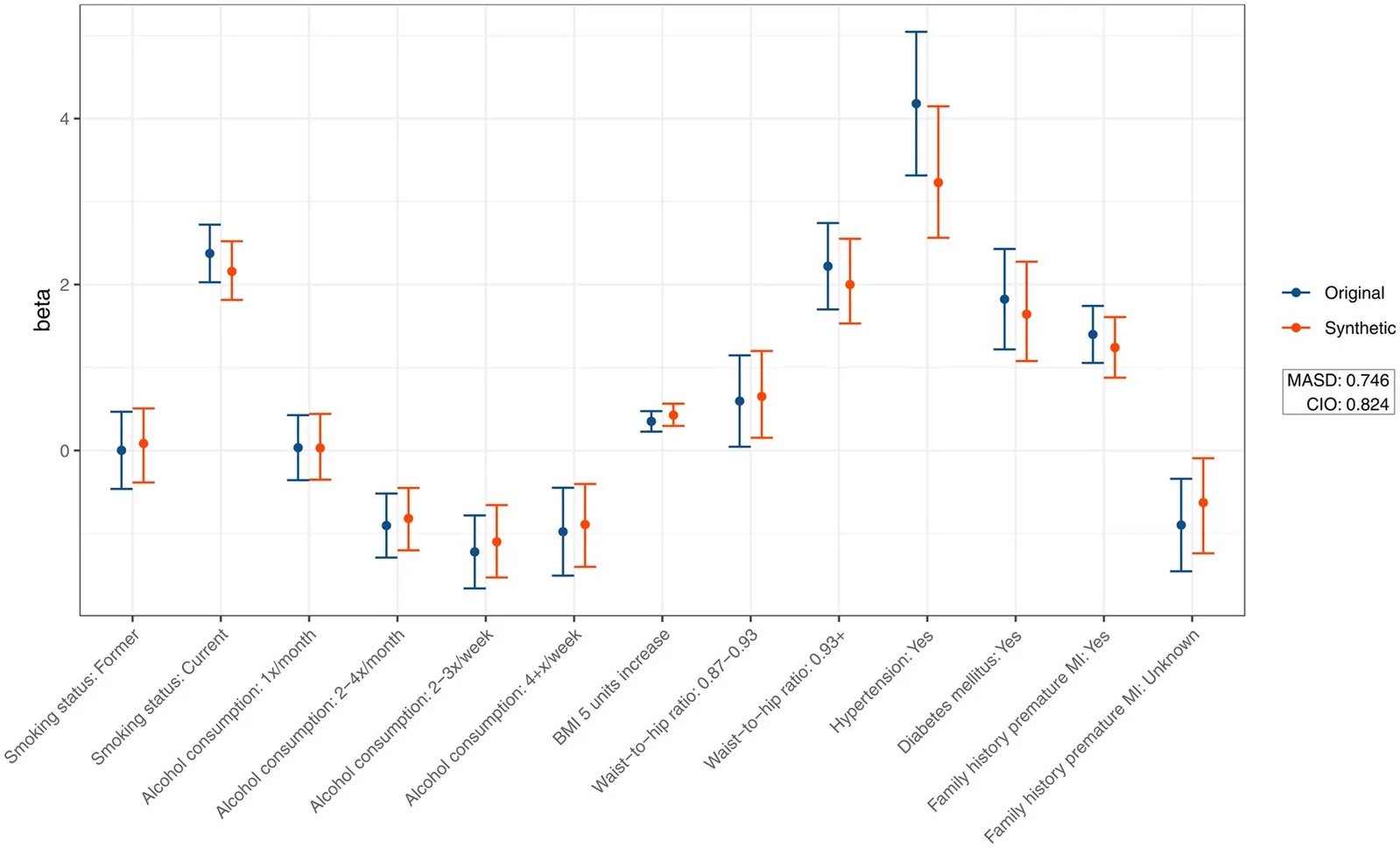
Replication of separate logistic regressions per variable, each adjusted for age, sex, country of birth, and years of school education, Table 2 in Wienbergen *et al*. [18]: associations between lifestyle and metabolic factors, as well as family history of premature MI and risk of early–onset MI. Median beta estimates and percentile-based 95% confidence intervals computed from the beta coefficient distribution across synthesis repetitions are reported for synthetic data. BMI, body mass index; MI, myocardial infarction; MASD, mean absolute standardized difference; CIO, mean confidence interval overlap.

Wienbergen *et al.* [18] used a dataset with 12 variables and 1713 participants. Table 2 and Fig. 3 (replicating Table 1 and the basic adjustment level logistic regressions of Table 2 in Wienbergen *et al.* [18]) show the performance of ARF-generated data replicating a typical ‘Table 1’ and logistic regression results. The synthetic data medians and 95% CIs closely resembled the original values in Table 2 (MASD = 0.664; CIO = 0.863). The largest relative median deviation was observed for the hypertension cases in the original control group of only six participants, but the synthetic data 95% CI still covered the original value. These findings align with Fig. 3, where synthetic data results were close to the original ones (MASD = 0.746; CIO = 0.824), while the hypertension variable posed the biggest difficulty as well. All results for this publication are presented in Supplement B.3, in the supplementary material.

**Table 2:** Replication of Table 1 in Wienbergen *et al.* [18]: Distribution of sociodemographic, lifestyle, and metabolic factors and family history of premature myocardial infarction according to cases and controls.

Variable	Value cases	Proportion cases	Value controls	Proportion controls
Number of participants (*n*)	522 (485-560)		1191 (1153-1228)	
	*522 (486-560)*		*1191 (1153-1227)*	
Age (years) (median)	42 (42-43)		42 (41-42)	
	*41 (41-42)*		*41 (40-41)*	
Age (years) (1st quartile)	39 (38-39)		38 (37-38)	
	*38 (38-39)*		*37 (37-38)*	
Age (years) (3rd quartile)	44 (44-44)		44 (43-44)	
	*43 (43-44)*		*43 (43-43)*	
Sex: Male (*n*)	434 (399-470)	0.83 (0.80-0.86)	927 (888-967)	0.78 (0.75-0.80)
	*435 (401-469)*	*0.83 (0.80-0.86)*	*927 (889-965)*	*0.78 (0.76-0.80)*
Sex: Female (*n*)	88 (71-106)	0.17 (0.14-0.20)	264 (235-295)	0.22 (0.20-0.25)
	*87 (71-105)*	*0.17 (0.14-0.20)*	*264 (236-292)*	*0.22 (0.20-0.24)*
Country of birth: Other countries (*n*)	102 (83-122)	0.20 (0.16-0.23)	230 (204-258)	0.19 (0.17-0.22)
	*104 (84-124)*	*0.20 (0.16-0.23)*	*229 (203-257)*	*0.19 (0.17-0.21)*
Country of birth: Germany (*n*)	420 (386-455)	0.80 (0.77-0.84)	961 (920-1001)	0.81 (0.78-0.83)
	*419 (384-455)*	*0.80 (0.77-0.84)*	*961 (921-1000)*	*0.81 (0.79-0.83)*
School education (years): *<*10 (*n*)	38 (27-51)	0.07 (0.05-0.10)	76 (60-94)	0.06 (0.05-0.08)
	*38 (27-52)*	*0.07 (0.05-0.10)*	*75 (59-93)*	*0.06 (0.05-0.08)*
School education (years): 10-11 (*n*)	382 (348-414)	0.73 (0.69-0.77)	241 (212-268)	0.20 (0.18-0.22)
	*371 (337-408)*	*0.71 (0.67-0.76)*	*251 (223-282)*	*0.21 (0.19-0.24)*
School education (years): 12 + (*n*)	102 (82-121)	0.20 (0.16-0.23)	874 (835-918)	0.73 (0.71-0.76)
	*113 (92-134)*	*0.21 (0.18-0.25)*	*864 (820-902)*	*0.73 (0.70-0.75)*
Smoking status: Never (*n*)	53 (40-67)	0.10 (0.08-0.13)	580 (543-617)	0.49 (0.46-0.52)
	*61 (47-78)*	*0.12 (0.09-0.15)*	*572 (532-612)*	*0.48 (0.45-0.51)*
Smoking status: Former (*n*)	39 (27-51)	0.07 (0.05-0.10)	324 (294-357)	0.27 (0.25-0.30)
	*46 (33-60)*	*0.09 (0.06-0.11)*	*317 (285-349)*	*0.27 (0.24-0.29)*
Smoking status: Current (*n*)	430 (395-465)	0.82 (0.79-0.86)	287 (257-318)	0.24 (0.22-0.27)
	*415 (382-452)*	*0.80 (0.76-0.83)*	302 (271-334)	*0.25 (0.23-0.28)*
Alcohol consumption: Never (*n*)	112 (92-133)	0.21 (0.18-0.25)	121 (101-142)	0.10 (0.08-0.12)
	*110 (91-130)*	*0.21 (0.18-0.25)*	*124 (103-144)*	*0.10 (0.09-0.12)*
Alcohol consumption: Once a month (*n*)	176 (153-201)	0.34 (0.30-0.38)	193 (169-219)	0.16 (0.14-0.18)
	*171 (147-197)*	*0.33 (0.29-0.37)*	*197 (172-224)*	*0.17 (0.15-0.19)*
Alcohol consumption: 2-4 times a month (*n*)	130 (108-151)	0.25 (0.21-0.29)	441 (405-477)	0.37 (0.34-0.40)
	*133 (113-155)*	*0.26 (0.22-0.29)*	*437 (404-473)*	*0.37 (0.34-0.39)*
Alcohol consumption: 2-3 times a week (*n*)	67 (52-84)	0.13 (0.10-0.16)	302 (273-332)	0.25 (0.23-0.28)
	*70 (54-86)*	*0.13 (0.10-0.16)*	*299 (269-329)*	*0.25 (0.23-0.28)*
Alcohol consumption: 4+ times a week (*n*)	37 (26-49)	0.07 (0.05-0.09)	134 (112-156)	0.11 (0.09-0.13)
	*38 (26-50)*	*0.07 (0.05-0.09)*	*133 (112-155)*	*0.11 (0.09-0.13)*
Body mass index (kg/m^2^) (median)	28.40 (27.76-28.88)		25.50 (25.30-25.80)	
	*28.62 (28.07-29.20)*		*25.68 (25.38-25.97)*	
Body mass index (kg/m^2^) (1st quartile)	25.10 (24.84-25.51)		23.00 (22.80-23.50)	
	*25.46 (24.98-25.96)*		*23.25 (22.98-23.55)*	
Body mass index (kg/m^2^) (3rd quartile)	31.79 (31.23-32.51)		28.35 (28.00-28.70)	
	*32.17 (31.52-32.86)*		*28.63 (28.23-29.03)*	
Body mass index: *<*25.0 (*n*)	123 (103-144)	0.24 (0.20-0.27)	514 (475-549)	0.43 (0.40-0.46)
	*111 (92-133)*	*0.21 (0.18-0.25)*	*511 (471-549)*	*0.43 (0.40-0.46)*
Body mass index: 25.0-29.9 (*n*)	208 (180-233)	0.40 (0.36-0.44)	482 (447-517)	0.40 (0.38-0.43)
	*205 (178-234)*	*0.39 (0.35-0.44)*	*472 (432-511)*	*0.40 (0.37-0.43)*
Body mass index: 30.0 + (*n*)	191 (165-217)	0.37 (0.33-0.41)	195 (170-220)	0.16 (0.14-0.18)
	*205 (178-234)*	*0.39 (0.35-0.44)*	*209 (180-236)*	*0.18 (0.15-0.20)*
Waist-to-hip ratio (median)	0.98 (0.97-0.99)		0.90 (0.89-0.90)	
	*0.98 (0.97-0.99)*		*0.90 (0.89-0.90)*	
Waist-to-hip ratio (1st quartile)	0.93 (0.91-0.95)		0.85 (0.84-0.86)	
	*0.92 (0.91-0.94)*		*0.85 (0.84-0.85)*	
Waist-to-hip ratio (3rd quartile)	1.03 (1.02-1.04)		0.95 (0.94-0.96)	
	*1.03 (1.02-1.04)*		*0.95 (0.94-0.96)*	
Hypertension: No (*n*)	391 (358-425)	0.75 (0.71-0.79)	1185 (1147-1223)	0.99 (0.99-1.00)
	*398 (365-433)*	*0.76 (0.73-0.80)*	*1178 (1140-1214)*	*0.99 (0.98-0.99)*
Hypertension: Yes (*n*)	131 (111-153)	0.25 (0.21-0.29)	6 (2-11)	0.01 (0.00-0.01)
	*124 (103-146)*	*0.24 (0.20-0.27)*	*13 (6-21)*	*0.01 (0.01-0.02)*
Diabetes mellitus: No (*n*)	461 (425-497)	0.88 (0.85-0.91)	1171 (1133-1209)	0.98 (0.98-0.99)
	*464 (429-500)*	*0.89 (0.86-0.92)*	*1168 (1131-1204)*	*0.98 (0.97-0.99)*
Diabetes mellitus: Yes (*n*)	61 (46-77)	0.12 (0.09-0.15)	20 (12-29)	0.02 (0.01-0.02)
	*58 (44-74)*	*0.11 (0.08-0.14)*	*22 (13-32)*	*0.02 (0.01-0.03)*
Family history of premature MI: No (*n*)	358 (326-391)	0.69 (0.65-0.72)	1012 (970-1052)	0.85 (0.83-0.87)
	*361 (328-393)*	*0.69 (0.65-0.73)*	*1008 (968-1048)*	*0.85 (0.83-0.87)*
Family history of premature MI: Yes (*n*)	144 (122-166)	0.28 (0.24-0.32)	97 (79-116)	0.08 (0.07-0.10)
	*138 (116-162)*	*0.27 (0.23-0.30)*	*103 (83-124)*	*0.09 (0.07-0.10)*
Family history of premature MI: Unknown (*n*)	20 (12-29)	0.04 (0.02-0.06)	82 (65-100)	0.07 (0.05-0.08)
	*23 (14-32)*	*0.04 (0.03-0.06)*	*79 (63-97)*	*0.07 (0.05-0.08)*
**MASD:** 0.664**; CIO:** 0.863				

Percentile-based 95% bootstrap confidence intervals are reported for original data results. Median and percentile-based 95% confidence intervals of synthetic data results are printed in italics. *n*, number of participants; MI, myocaridal infarction; MASD, mean absolute standardized difference; CIO, mean confidence interval overlap.

#### *Replication results for Breau et al.* [19]

**Figure 4:**
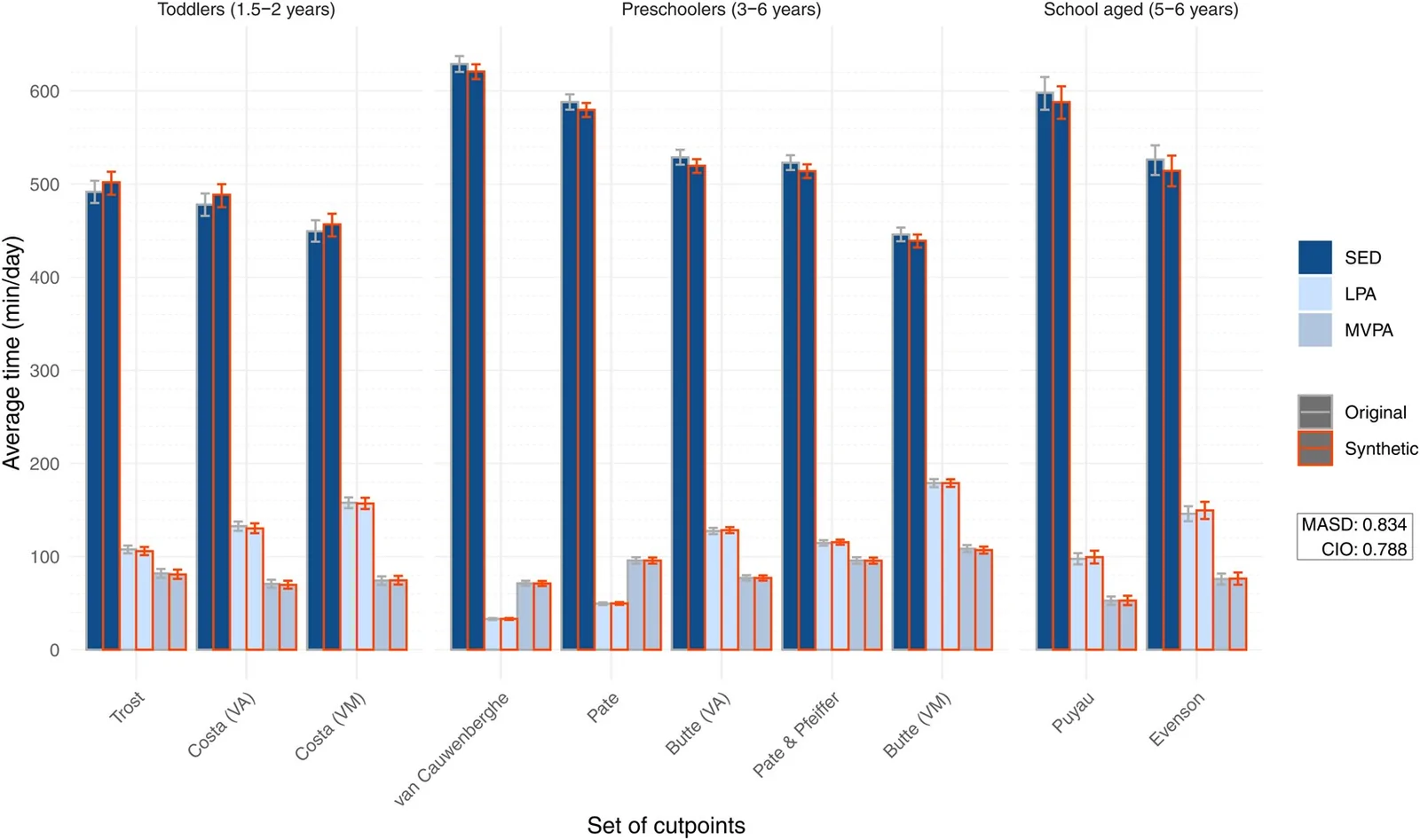
Replication of Figure 2 in Breau *et al*. [19]: calculated average valid wear time minutes per day spent in SED, LPA, and MVPA according to age-appropriate ActiGraph cutpoint sets by age group. Percentile-based 95% bootstrap confidence intervals are reported for original data results. Median and percentile-based 95% confidence intervals of synthetic data results are reported. SED, sedentary behaviour; LPA, light physical activity; MVPA, moderate to vigorous physical activity; VA, vertical axis; VM, vector magnitude; MASD, mean absolute standardized difference; CIO, mean confidence interval overlap.

Containing 262 participants and 28 variables, three of which were categorical with a total of 34 categories, the data characteristics in Breau *et al.* [19] present a challenge for generative machine learning methods. However, the synthetic data results shown in Fig. 4 and Supplementary Fig. 6, in the supplementary material (replicating Figures 2 and 1 in Breau *et al.* [19], respectively) matched the original findings well (MASD = 0.834; CIO = 0.788). Similarly, the replication results in Supplementary Fig. 8, in the supplementary material (replicating Fig. 3 in Breau *et al.* [19]) exhibited strong resemblance, allowing all main conclusions to remain valid with synthetic data. While the synthetic data results in Supplement Table 11, in the supplementary material (replicating Table 3 in Breau *et al.* [19]) yielded closely matching mean estimates, they showed lower precision for standard deviations and subgroup sizes, likely due to the relatively low participants-to-dimensionality ratio and the presence of derived variables (age group and body mass index). All evaluation results are provided in Supplement B.4, in the supplementary material.

### Effect of dimensionality and derived variables (task-specific synthesis)

#### *Replication results for Berger et al.* [20]

**Figure 5:**
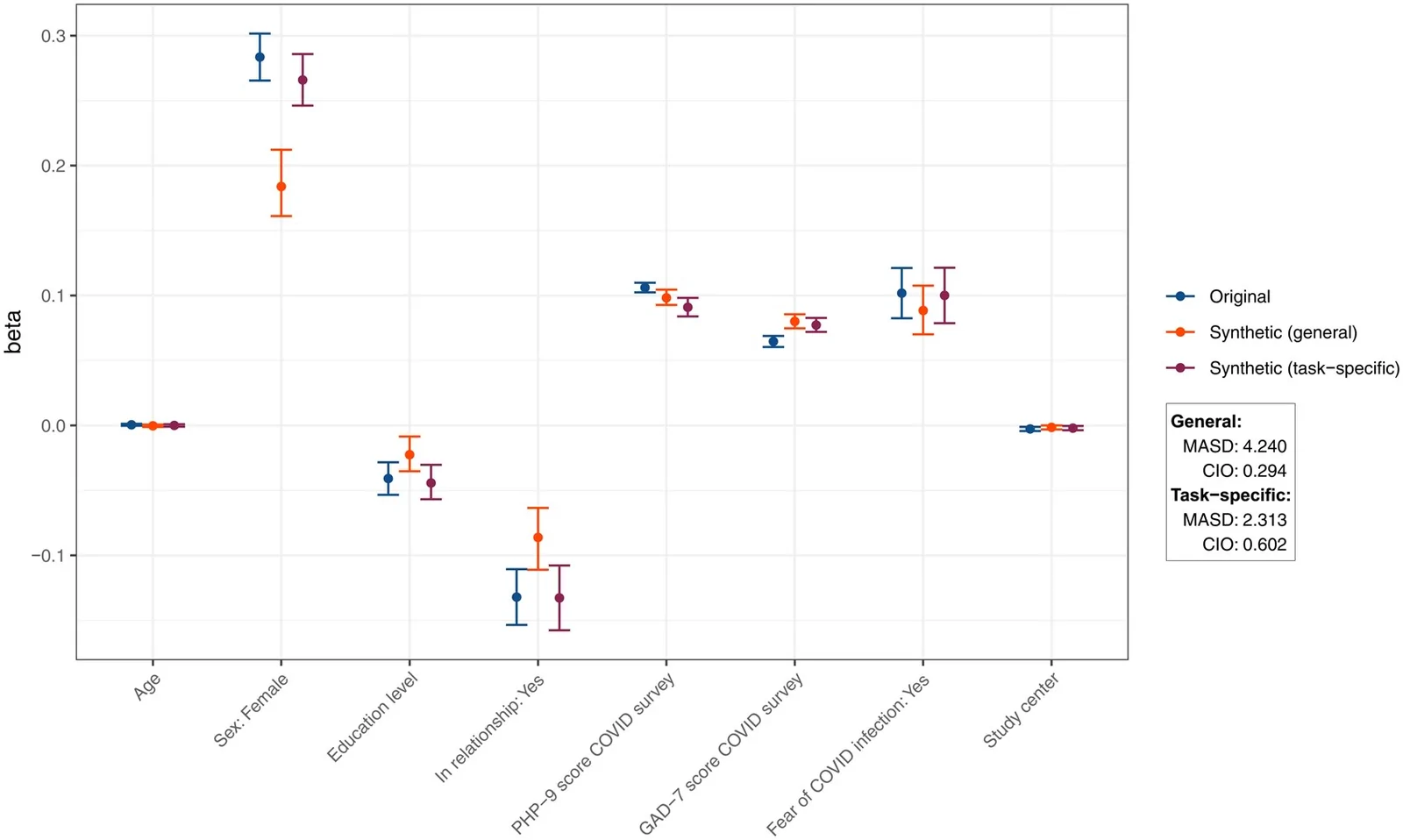
Replication of multivariable linear regression, Table 3 in Berger *et al*. [20], with general and task-specific synthesis: relationship between perceived loneliness and sociodemographic factors as well as symptoms of depression and anxiety among German National Cohort (NAKO Gesundheitsstudie) participants in May 2020. Median beta estimates and percentile-based 95% confidence intervals computed from the beta coefficient distribution across synthesis repetitions are reported for synthetic data. PHQ-9, nine-item Patient Health Questionnaire; GAD-7, Generalized Anxiety Disorder seven-item scale; MASD, mean absolute standardized difference; CIO, mean confidence interval overlap.

The data used in Berger *et al.* [20] contained 113 527 participants and 47 variables, 44 of which were categorical with a total count of 230 categories. After aggregating PHQ-9 [41], GAD-7 [42], and loneliness questionnaire items to a sum score each, nine final variables were used in the study’s linear regression analysis. Figure 5 (replicating Table 3 in Berger *et al.* [20]) shows the resulting *β*-estimates for the association between perceived loneliness as the dependent variable and the eight independent variables. The effect of the reduced dimensionality and complexity on the synthesis quality was apparent comparing general and task-specific synthesis results: While general synthesis results (MASD = 4.24; CIO = 0.294) in many cases overlapped with the original results but struggled especially for the sex and relationship variable, task-specific synthesis results (MASD = 2.313; CIO = 0.602) were consistently closer. Aligning observations were made in Supplement Table 15, in the supplementary material (replicating Table 1 in Berger *et al.* [20]), where task-specific synthesis (MASD = 1.792; CIO = 0.747) yielded estimates closer to the original results compared to general synthesis (MASD = 9.161; CIO = 0.438) for the derived age group variable and for the standard deviations of PHQ-9 and GAD-7 sum scores. All results for this publication are reported in Supplement B.5, in the supplementary material.

#### *Replication results for Tanoey et al.* [21]

**Figure 6:**
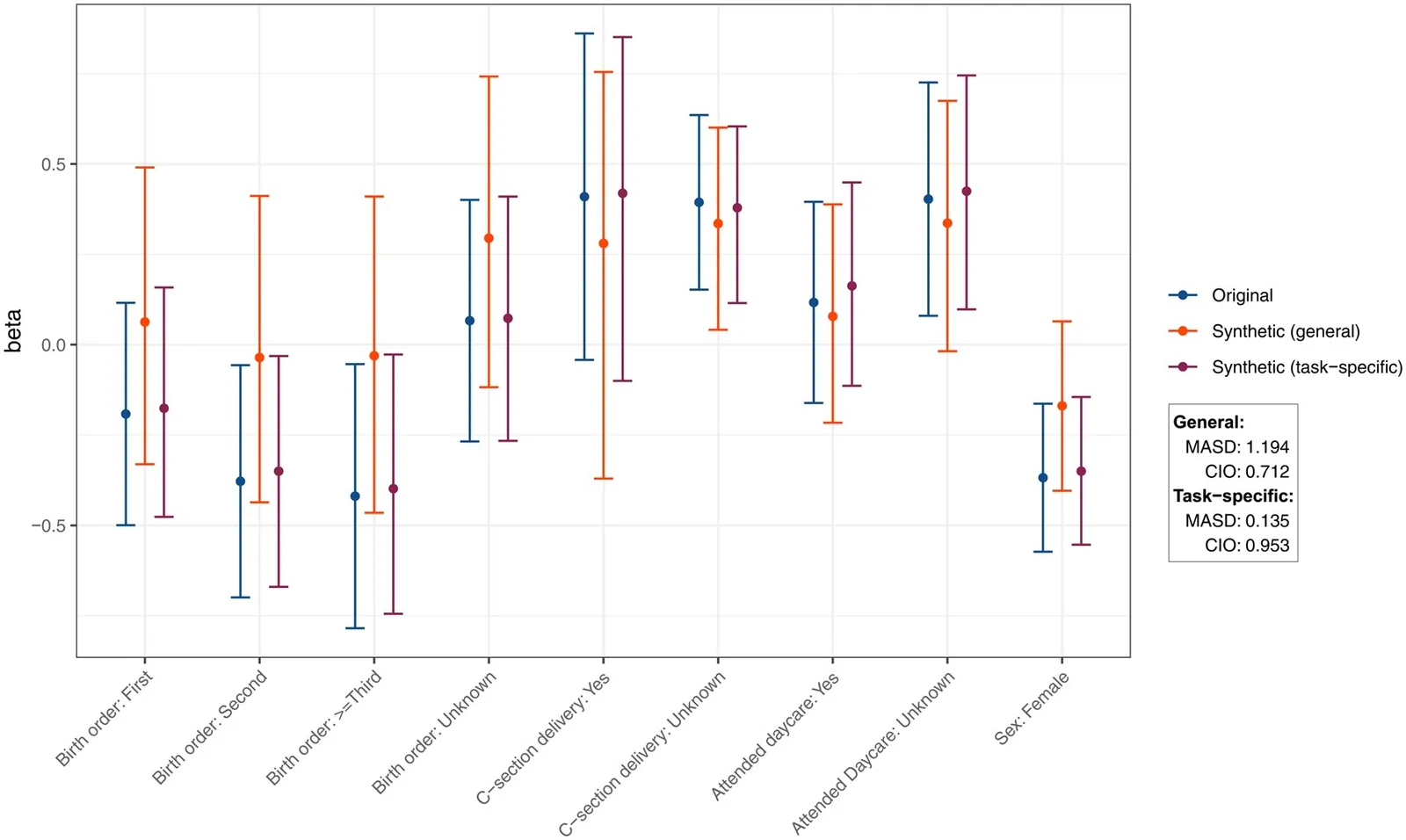
Replication of univariable Cox regressions, Table 2 in Tanoey *et al*. [21], with general and task-specific synthesis: type 1 diabetes univariable Cox regression estimates. Median beta estimates and percentile-based 95% confidence intervals computed from the beta coefficient distribution across synthesis repetitions are reported for synthetic data. C-section, caesarean section; MASD, mean absolute standardized difference; CIO, mean confidence interval overlap.

The Cox regression results in Fig. 6 (replicating the univariable Cox regression on type 1 diabetes risk given in Table 2 in Tanoey *et al.* [21]) support the previous findings. A complex identification algorithm for type 1 diabetes combined five variables and depended on the presence of missing values in some of these. A relatively low share of type 1 diabetes cases (371 out of 101 570 participants) was identified. The results performed on the full original dataset with 19 variables (12 categorical variables with a total count of 42 categories) were comparable to the original ones (MASD = 1.194; CIO = 0.712) but improved using a task-specific dataset of eight derived variables (MASD = 0.135; CIO = 0.953). The further replication results (see supplementary material for Supplement B.6) showed a similar pattern. The effect of variable derivations and the small subset size was most evident in Supplementary Fig. 14, in the supplementary material, where only type 1 diabetes cases were considered (general synthesis: MASD = 2.448, CIO = 0.436; task-specific synthesis: MASD = 0.422, CIO = 0.901).

### Competitive performance: privacy, generalization, and runtime

Across datasets, ARF consistently ranked among the synthesizers with the highest utility, with only minor differences for minimum node sizes of 2, 5, and 10. Lower node sizes generally improved utility but, especially in the case of the mainly categorical Berger *et al.* [20] increased privacy risks, reflecting the expected utility–privacy trade-off. ARF matched or exceeded all alternatives in the utility–privacy trade-off across four of six datasets, ranking second-best in the others.

These results remained consistent across the generalization analysis, confirming that ARF did not merely reproduce the training distribution.

Runtime comparisons further highlighted the efficiency of ARF. For the largest dataset, Berger *et al.* [20], ARF completed training and sampling within 35 seconds, whereas alternative approaches required substantially longer runtimes (six to 36 minutes). Full results are provided Supplement C, in the supplementary material.

## Discussion

Summarizing the results, ARF consistently generated synthetic datasets that closely replicated original study findings across a range of data sizes and dimensionalities without tuning. Various descriptive and inferential analyses were repeated using synthetic ARF data with similar outcomes. In most of the cases, median results on synthetic data were close and the percentile-based 95% CIs well aligned. These findings correspond to existing benchmark studies reporting competitive ARF performance on non-health datasets using different evaluation metrics [12, 14].

We found that a higher participants-to-dimensionality ratio generally led to closer resemblance. Whenever possible, we therefore recommend restricting the data to variables used in subsequent analyses and performing any necessary variable derivation before synthesis. While such task-specific preprocessing is feasible for applications such as data balancing or augmentation, it may not always be possible for privacy-preserving data sharing scenarios where preprocessing must be performed by the data provider.

The extended evaluation further showed that ARF typically achieves a favourable balance between utility and privacy risk compared with commonly used tabular data synthesizers while operating orders of magnitude faster. The minimum node size was found to influence this trade-off: smaller values generally improved utility but increased privacy risks. In practice, slightly larger values (e.g. 5 or 10 instead of the default of 2) may therefore provide a more balanced default in privacy-sensitive applications.

A key strength of this study is its epidemiology-focused validation across multiple real-world datasets and analysis types. Unlike typical machine-learning papers, which rarely perform epidemiology-relevant analyses and often report metrics that are difficult to interpret, we provide application-oriented assessments; moreover, privacy aspects—especially crucial in epidemiological contexts—are frequently omitted, as their evaluation is non-trivial.

Future directions could include developing a differentially private [43] variant of ARF or an extension for extremely high-dimensional data, such as omics datasets.

## Conclusion

This study demonstrated that ARF is a practical and valuable tool for synthesizing tabular epidemiological data, producing synthetic datasets that closely replicate descriptive and inferential findings across diverse datasets and analyses. In the abscence of a universal gold standard for tabular data synthesis, ARF offers a competitive balance between utility and privacy risk compared with commonly used synthesizers, while remaining computationally efficient.

## Ethics approval

The German National Cohort (NAKO Gesundheitsstudie, NAKO) study is performed with the approval of the relevant local ethics committees, and is in accordance with national law and with the Declaration of Helsinki of 1975 (in the current, revised version). The Bremen STEMI Registry U45 Study (BSR-U45) was approved by the ethics committee of the Ärztekammer Bremen, Germany. All participants gave written informed consent. The study was performed in accordance to the Declaration of Helsinki, with approved protocols conducted by trained investigators subject to independent ethical review and under oversight by a properly convened committee. The Guelph Familiy Health Study (GFHS) was conducted in accordance with the Declaration of Helsinki and approved by the University of Guelph Research Ethics Board (REB 17-07-003).

## Supplementary Material

dyag164_Supplementary_Data

## Data Availability

The German National Cohort (NAKO Gesundheitsstudie, NAKO) data are not openly available due to data protection measures. However, scientists can apply for data access following the official usage regulations and upon formal request to the NAKO use and access committee (https://transfer.nako.de). Participant data from the Bremen STEMI Registry U45 Study (BSR-U45) are not publicly available. Due to University of Guelph Research Ethics Board restrictions and participant confidentiality, no Guelph Family Health Study (GFHS) participant data are publicly available. The GFHS welcomes outside collaborators. Interested investigators can contact GFHS investigators to explore this option, which preserves participant confidentiality and meets the requirements of the University of Guelph Research Ethics Board, to protect human subjects.
