# Supplementary material for: Can synthetic data reproduce real-world findings in epidemiology? A replication study using adversarial random forests
Source: Int J Epidemiol. 2026 Aug 29;55(5):dyag164. doi: 10.1093/ije/dyag164 (PMC13525842; doi:10.1093/ije/dyag164)
Supplement: Supplementary file 1 [file dyag164_supplementary_data.zip › ARF_SynthEpiRep_supplement.pdf]

Supplementary Material

Contents

A Evaluation details 3

B Complete set of replication results 4

B.1 Schikowski et al.[5] 4
Table S1. Replication of Figure 2 4
Figure S1. Replication of Figure 2 5
Table S2. Replication of Figures 3 and 4 6
Figure S2. Replication of Figures 3 and 4 7
Table S3. Replication of Figures 5 and 6 8
B.2 Fischer et al.[3] 9
Table S4. Replication of Table 1 9
Table S5. Replication of Figure 2 11
Table S6. Replication of Figure 3 12
Figure S3. Replication of Figure 3 13
Table S7. Replication of Figure 4 14
Figure S4. Replication of Figure 5 15
B.3 Wienbergen et al.[6] 16
Table S8. Replication of Table 1 16
Table S9. Replication of Table 2 17
Figure S5. Replication of separate logistic regressions per variable, each adjusted for age, sex, country of birth, and years of school education - Table 2 18
Table S10. Replication of Table 3 19
B.4 Breau et al.[7] 20
Table S11. Replication of Table 3 20
Table S12. Replication of Figure 1 21
Figure S6. Replication of Figure 1 21
Table S13. Replication of Figure 2 22
Figure S7. Replication of Figure 2 22
Table S14. Replication of Figure 3 23
Figure S8. Replication of Figure 3 23
B.5 Berger et al.[8] 24
Table S15. Replication of Table 1 (general and task-specific synthesis) 24
Table S16. Replication of Table 2 25
Table S17. Replication of multivariable linear regression - Table 3 (general and task-specific synthesis) 26
Figure S9. Replication of multivariable linear regression - Table 3 (general and task-specific synthesis) 26
Table S18. Replication of Figure 1 27
Figure S10. Replication of Figure 1 27
Table S19. Replication of Figure 2 28
Figure S11. Replication of Figure 2 28
Table S20. Replication of Figure 3 29
Figure S12. Replication of Figure 3 29
B.6 Tanoey et al.[9] 30
Table S21. Replication of Table 1 (general and task-specific synthesis) 30

Table S22. Replication of Table 2 . . . 33
Table S23. Replication of univariable Cox regressions - Table 2 (general and task-specific synthesis) 34
Figure S13. Replication of univariable Cox regressions - Table 2 (general and task-specific synthesis) 34
Table S24. Replication of Figure 2 (general and task-specific synthesis) . . . 35
Figure S14. Replication of Figure 2 (general and task-specific synthesis) . . . 35

C Comparative analysis of ARF performance across varying minimum node sizes and alternative synthesizers **36**
C.1 Utility-privacy trade-off . . . 36
Figure S15. Utility (MASD) for downstream descriptive analyses from Schikowski et al.[5] (Figure 1) and Breau et al.[7] (Figure 4) vs. privacy risk MIA/AIA . . . 38
Figure S16. Utility (WD) for downstream multivariate distribution analysis from Fischer et al.[3] (Figure 2) vs. privacy risk MIA/AIA . . . 39
Figure S17. Utility (CIO) for downstream regression analyses from Wienbergen et al.[6] (Figure 3), Berger et al.[8] (Figure 5) and Tanoey et al.[9] (Figure 6) vs. privacy risk MIA/AIA . . . 40
C.2 Generalisation to unseen test data . . . 41
Figure S18. Distributional similarity (WD Syn-Test) vs. privacy risk MIA . . . 42
C.3 Runtime . . . 43
Figure S19. Synthesis runtime for Breau et al.[7] and Berger et al.[8] . . . 43

A Evaluation details

In this section, we provide additional details on the evaluation of synthetic data generated by ARF. The evaluation assesses bias in the reproduction of the original analytical results and examines the extent to which statistical uncertainty was preserved, while also quantifying variability introduced by stochastic model training.

We measured the uncertainty in the original analyses using 95% bootstrap percentile confidence intervals[1] for every estimate based on 2 000 bootstrap resamples. These were reported alongside the original estimates. For synthetic data, we repeated ARF training 100 times, sampled 20 synthetic datasets of the same size as the original data from each trained ARF model, and reported the median of the estimates across all 2 000 synthetic datasets, together with the corresponding 95% percentile confidence intervals.

Summary measures were reported to capture deviations between the original and synthetic estimates across all reported statistics within a table or plot. Bias was quantified using the mean absolute standardized difference (MASD)[2] between the original estimates and the median synthetic estimates:

$$\text{MASD} = \frac{1}{n_{\text{stat}}} \sum_{i=1}^{n_{\text{stat}}} \frac{\left|\text{median}\left(\widehat{\text{stat}}^i_{\text{synthetic}}\right) - \widehat{\text{stat}}^i_{\text{original}}\right|}{\text{SE}\left(\widehat{\text{stat}}^i_{\text{original}}\right)} \in \mathbb{R}_0^+$$

The standard error of the original estimates were obtained from the bootstrap distribution, ensuring a uniform and comparable measure of uncertainty across all statistics, which might be heterogeneous and not amenable to simple analytical variance formulas. MASD values are non-negative, with an optimum at 0.

Agreement in uncertainty was assessed using the mean confidence interval overlap (CIO)[2]:

$$\text{CIO} = \frac{1}{n_{\text{stat}}} \sum_{i=1}^{n_{\text{stat}}} \frac{1}{2} \left(\frac{\|\bigcap\{\text{CI}^i_{\text{original}}, \text{CI}^i_{\text{synthetic}}\}\|}{\|\text{CI}^i_{\text{original}}\|} + \frac{\|\bigcap\{\text{CI}^i_{\text{original}}, \text{CI}^i_{\text{synthetic}}\}\|}{\|\text{CI}^i_{\text{synthetic}}\|} \right) \in [0,1]$$

,

This metric measures the overlap between two confidence intervals as the average proportion of their intersection relative to each interval. Non-overlapping intervals yield $\text{CIO} = 0$, identical intervals $\text{CIO} = 1$.

MASD and CIO were reported for all replicated tables and plots. An exception is the subgroup distribution analysis in Figure 2 (Fischer et al.[3]), where deviations between original and synthetic distributions were quantified using the Wasserstein distance (WD)[4]. Following normalisation to the original data ranges, WD values range from 0 to 1, with lower values indicating higher distributional similarity.

B Complete set of replication results

B.1 Schikowski et al.[5]

Table S1. Replication of Figure 2

Sex	Age group	Blood pressure measure	Avg. meas. 1/2 - meas. 2 (mean)
Male	20-24	Systolic	1.72 (1.51-1.91)
			1.61 (1.34-1.88)
		Diastolic	0.87 (0.73-1.02)
			0.87 (0.68-1.07)
	25-34	Systolic	1.26 (1.17-1.35)
			1.32 (1.21-1.45)
		Diastolic	0.73 (0.67-0.80)
			0.71 (0.62-0.80)
	35-44	Systolic	1.25 (1.17-1.33)
			1.29 (1.18-1.41)
		Diastolic	0.60 (0.55-0.65)
			0.61 (0.54-0.68)
	45-54	Systolic	1.41 (1.35-1.46)
			1.46 (1.37-1.55)
		Diastolic	0.61 (0.58-0.65)
			0.63 (0.59-0.68)
	55-64	Systolic	1.85 (1.79-1.91)
			1.85 (1.77-1.94)
		Diastolic	0.73 (0.70-0.77)
			0.73 (0.68-0.78)
	65-69	Systolic	2.15 (2.07-2.24)
			2.17 (2.04-2.29)
		Diastolic	0.79 (0.74-0.84)
			0.79 (0.72-0.86)
Female	20-24	Systolic	1.19 (1.03-1.35)
			1.22 (1.02-1.42)
		Diastolic	0.95 (0.85-1.05)
			0.91 (0.74-1.05)
	25-34	Systolic	1.24 (1.16-1.31)
			1.22 (1.12-1.33)
		Diastolic	0.71 (0.66-0.76)
			0.72 (0.65-0.79)
	35-44	Systolic	1.27 (1.19-1.34)
			1.29 (1.19-1.42)
		Diastolic	0.69 (0.65-0.74)
			0.68 (0.62-0.75)
	45-54	Systolic	1.48 (1.42-1.53)
			1.50 (1.43-1.59)
		Diastolic	0.69 (0.66-0.72)
			0.70 (0.66-0.75)
	55-64	Systolic	1.80 (1.74-1.87)
			1.84 (1.75-1.92)
		Diastolic	0.76 (0.73-0.79)
			0.76 (0.71-0.80)
	65-69	Systolic	2.31 (2.22-2.40)
			2.29 (2.14-2.42)
		Diastolic	0.83 (0.78-0.88)
			0.82 (0.75-0.88)
**MASD:** 0.579; **CIO:** 0.824			

Differences of mean blood pressure values (in mmHg) using the mean of first and second measurement or the second measurement only by sex and age group (in years). Percentile-based 95% bootstrap confidence intervals are reported for original data results. Median and percentile-based 95% confidence intervals of synthetic data results are printed in orange. avg., average; meas., measurement; MASD, mean absolute standardised difference; CIO, confidence interval overlap

Figure S1. Replication of Figure 2

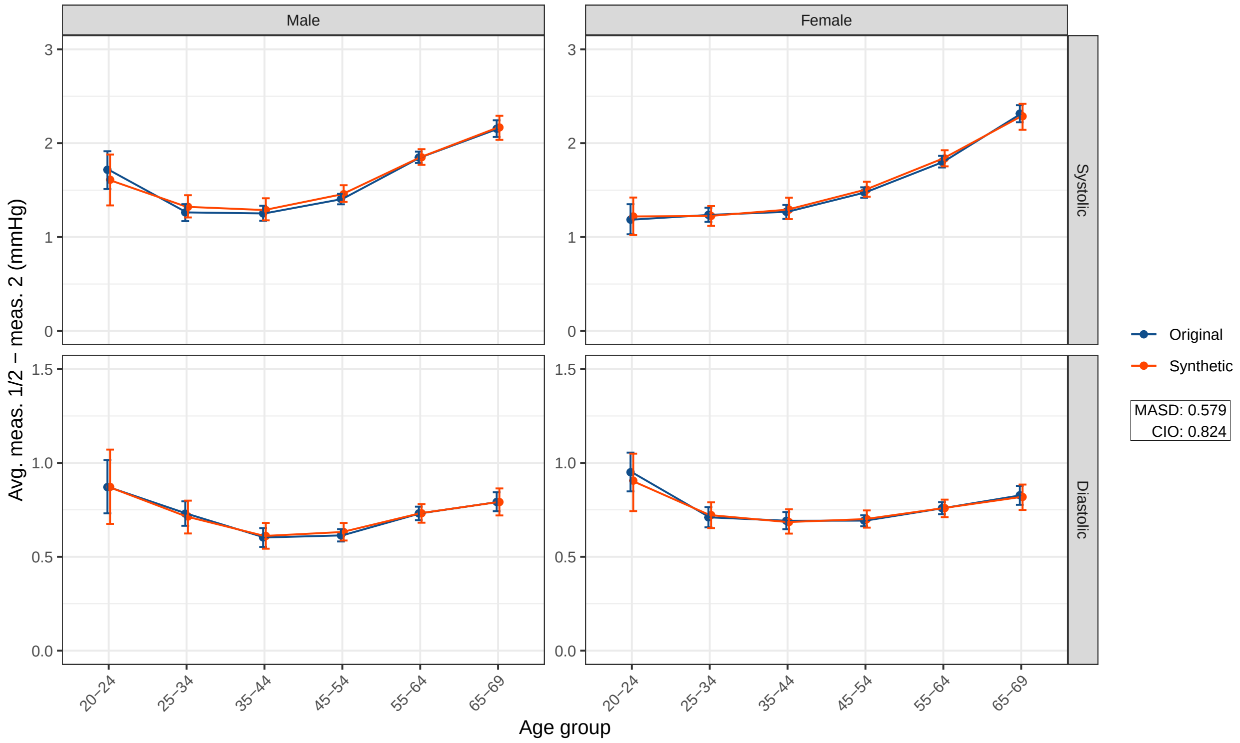

Differences of mean blood pressure values (in mmHg) using the mean of first and second measurement or the second measurement only by sex and age group (in years). Percentile-based 95% bootstrap confidence intervals are reported for original data results. Median and percentile-based 95% confidence intervals of synthetic data results are reported. avg., average; meas., measurement; MASD, mean absolute standardised difference; CIO, confidence interval overlap

Table S2. Replication of Figures 3 and 4

Sex	Age group	Blood pressure measure	Aggregation	Value
Male	20-24	Systolic	Mean	126.22 (125.50-126.91)<br>126.01 (125.09-126.97)
			SD	11.47 (10.91-12.00)<br>11.22 (10.59-11.90)
		Diastolic	Mean	72.14 (71.64-72.65)<br>72.39 (71.69-73.24)
			SD	8.10 (7.69-8.48)<br>7.93 (7.44-8.42)
	25-34	Systolic	Mean	127.29 (126.96-127.64)<br>127.09 (126.68-127.55)
			SD	11.61 (11.32-11.91)<br>11.57 (11.25-11.98)
		Diastolic	Mean	75.49 (75.24-75.75)<br>75.63 (75.23-76.21)
			SD	8.42 (8.21-8.63)<br>8.50 (8.24-8.83)
	35-44	Systolic	Mean	128.61 (128.29-128.94)<br>128.67 (128.12-129.29)
			SD	12.39 (12.12-12.69)<br>12.64 (12.28-13.06)
		Diastolic	Mean	80.30 (80.05-80.53)<br>80.00 (79.56-80.41)
			SD	9.27 (9.08-9.48)<br>9.26 (9.04-9.50)
	45-54	Systolic	Mean	131.83 (131.59-132.07)<br>132.04 (131.70-132.52)
			SD	14.40 (14.17-14.62)<br>14.62 (14.33-15.04)
		Diastolic	Mean	82.96 (82.79-83.12)<br>82.81 (82.51-83.08)
			SD	9.64 (9.51-9.76)<br>9.74 (9.57-9.90)
	55-64	Systolic	Mean	135.47 (135.19-135.75)<br>135.76 (135.32-136.50)
			SD	16.68 (16.43-16.91)<br>16.84 (16.52-17.28)
		Diastolic	Mean	82.51 (82.34-82.67)<br>82.56 (82.32-82.92)
			SD	9.96 (9.83-10.09)<br>10.06 (9.89-10.26)
	65-69	Systolic	Mean	138.60 (138.22-139.01)<br>138.57 (137.80-139.17)
			SD	17.85 (17.51-18.20)<br>17.59 (17.13-18.03)
		Diastolic	Mean	80.59 (80.35-80.80)<br>80.63 (80.14-80.98)
			SD	10.13 (9.95-10.31)<br>10.00 (9.76-10.27)
Female	20-24	Systolic	Mean	114.78 (114.23-115.30)<br>114.78 (113.93-115.61)
			SD	10.22 (9.76-10.70)<br>10.04 (9.47-10.65)
		Diastolic	Mean	70.97 (70.59-71.37)<br>71.05 (70.50-71.62)
			SD	7.64 (7.33-7.98)<br>7.50 (7.10-7.93)
	25-34	Systolic	Mean	114.74 (114.44-115.02)<br>114.92 (114.47-115.49)
			SD	10.72 (10.43-11.02)<br>10.84 (10.55-11.39)
		Diastolic	Mean	72.70 (72.46-72.92)<br>72.70 (72.35-73.04)
			SD	8.53 (8.32-8.73)<br>8.43 (8.21-8.68)
	35-44	Systolic	Mean	117.41 (117.10-117.69)<br>117.67 (116.79-118.62)
			SD	12.83 (12.57-13.11)<br>12.95 (12.50-13.44)
		Diastolic	Mean	75.64 (75.42-75.85)<br>75.47 (74.86-75.94)
			SD	9.41 (9.23-9.58)<br>9.32 (9.07-9.54)
	45-54	Systolic	Mean	123.17 (122.93-123.41)<br>123.57 (123.21-124.06)
			SD	15.44 (15.23-15.65)<br>15.64 (15.35-16.03)
		Diastolic	Mean	78.60 (78.45-78.75)<br>78.60 (78.37-78.89)
			SD	9.82 (9.69-9.94)<br>9.88 (9.74-10.03)
	55-64	Systolic	Mean	129.91 (129.64-130.19)<br>130.15 (129.67-131.01)
			SD	17.53 (17.29-17.75)<br>17.73 (17.40-18.14)
		Diastolic	Mean	79.50 (79.34-79.65)<br>79.61 (79.37-80.00)
			SD	9.76 (9.64-9.88)<br>9.87 (9.71-10.11)
	65-69	Systolic	Mean	135.72 (135.32-136.14)<br>135.65 (134.69-136.35)
			SD	18.59 (18.25-18.93)<br>18.41 (17.99-18.88)
		Diastolic	Mean	78.84 (78.63-79.08)<br>78.89 (78.33-79.26)
			SD	9.86 (9.69-10.03)<br>9.77 (9.56-9.99)

MASD: 1.095; **CIO:** 0.729

Distribution of systolic and diastolic blood pressure (second measurement; in mmHg) by sex and age group (in years). Percentile-based 95% bootstrap confidence intervals are reported for original data results. Median and percentile-based 95% confidence intervals of synthetic data results are printed in orange. SD, standard deviation; MASD, mean absolute standardised difference; CIO, confidence interval overlap

Figure S2. Replication of Figures 3 and 4

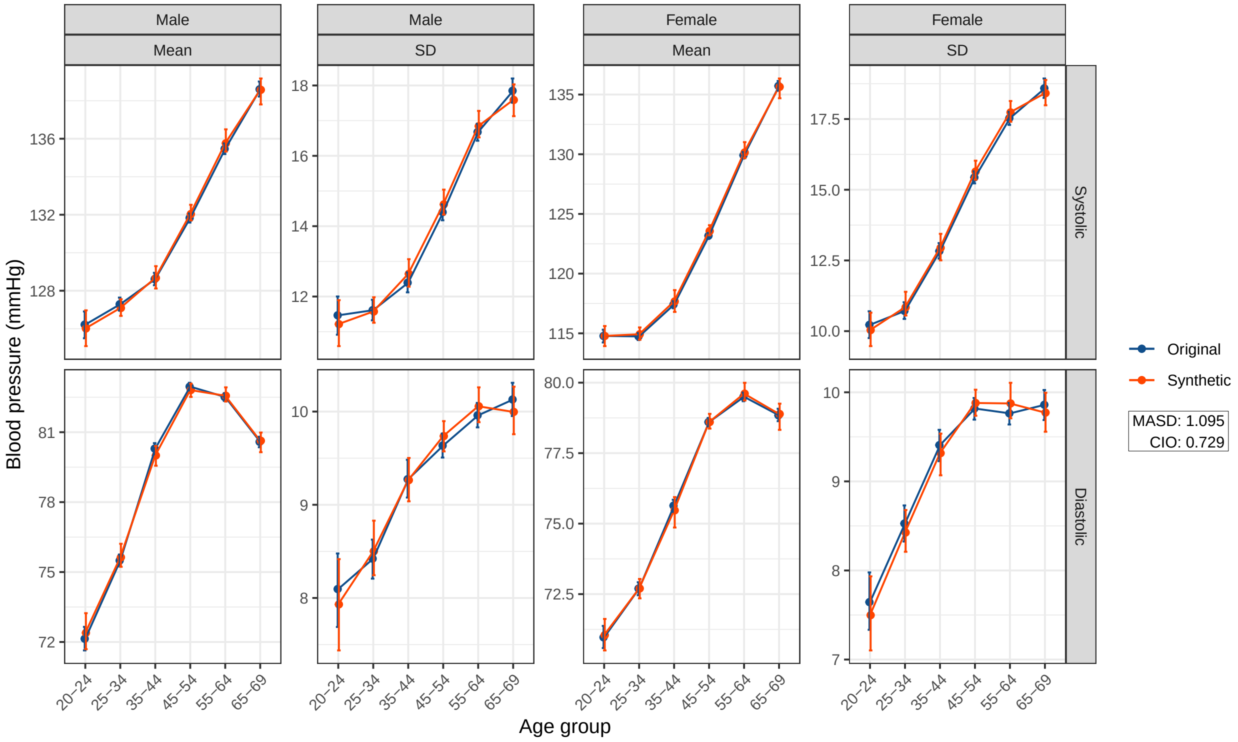

Distribution of systolic and diastolic blood pressure (second measurement; in mmHg) by sex and age group (in years). Percentile-based 95% bootstrap confidence intervals are reported for original data results. Median and percentile-based 95% confidence intervals of synthetic data results are reported. SD, standard deviation; MASD, mean absolute standardised difference; CIO, confidence interval overlap

Table S3. Replication of Figures 5 and 6

Sex	Age group	Hypertension status	Proportion using avg. meas. 1/2	Proportion using meas. 2
Male	20-24	Normotension	0.82 (0.79-0.84)	0.83 (0.81-0.85)
			0.83 (0.80-0.86)	0.84 (0.81-0.87)
		Hypertension known controlled	0.03 (0.02-0.04)	0.03 (0.02-0.04)
			0.03 (0.02-0.04)	0.03 (0.02-0.04)
		Hypertension known uncontrolled	0.02 (0.01-0.02)	0.01 (0.01-0.02)
			0.01 (0.01-0.02)	0.01 (0.01-0.02)
		Hypertension unknown uncontrolled	0.14 (0.12-0.16)	0.12 (0.10-0.14)
			0.13 (0.10-0.16)	0.11 (0.09-0.14)
	25-34	Normotension	0.78 (0.77-0.79)	0.79 (0.78-0.81)
			0.78 (0.76-0.80)	0.79 (0.77-0.81)
		Hypertension known controlled	0.04 (0.04-0.05)	0.05 (0.04-0.05)
			0.05 (0.04-0.05)	0.05 (0.04-0.06)
		Hypertension known uncontrolled	0.03 (0.03-0.04)	0.03 (0.02-0.03)
			0.03 (0.02-0.04)	0.03 (0.02-0.03)
		Hypertension unknown uncontrolled	0.14 (0.13-0.15)	0.13 (0.12-0.14)
			0.14 (0.13-0.16)	0.13 (0.12-0.15)
	35-44	Normotension	0.68 (0.66-0.69)	0.69 (0.67-0.70)
			0.67 (0.65-0.70)	0.68 (0.65-0.70)
		Hypertension known controlled	0.08 (0.07-0.09)	0.08 (0.08-0.09)
			0.08 (0.07-0.09)	0.09 (0.08-0.09)
		Hypertension known uncontrolled	0.07 (0.07-0.08)	0.07 (0.06-0.08)
			0.07 (0.06-0.08)	0.07 (0.06-0.08)
		Hypertension unknown uncontrolled	0.17 (0.16-0.18)	0.16 (0.15-0.17)
			0.17 (0.16-0.19)	0.17 (0.15-0.19)
	45-54	Normotension	0.52 (0.51-0.53)	0.53 (0.52-0.54)
			0.51 (0.49-0.52)	0.52 (0.50-0.53)
		Hypertension known controlled	0.13 (0.13-0.14)	0.14 (0.13-0.14)
			0.14 (0.13-0.15)	0.14 (0.13-0.15)
		Hypertension known uncontrolled	0.14 (0.13-0.14)	0.13 (0.12-0.14)
			0.14 (0.13-0.15)	0.13 (0.13-0.14)
		Hypertension unknown uncontrolled	0.22 (0.21-0.22)	0.20 (0.20-0.21)
			0.22 (0.20-0.23)	0.21 (0.20-0.22)
	55-64	Normotension	0.33 (0.32-0.34)	0.34 (0.34-0.35)
			0.33 (0.32-0.35)	0.34 (0.33-0.36)
		Hypertension known controlled	0.23 (0.22-0.24)	0.25 (0.24-0.25)
			0.22 (0.21-0.23)	0.24 (0.23-0.25)
		Hypertension known uncontrolled	0.24 (0.23-0.24)	0.22 (0.21-0.23)
			0.24 (0.23-0.26)	0.23 (0.22-0.24)
		Hypertension unknown uncontrolled	0.20 (0.19-0.21)	0.19 (0.18-0.19)
			0.20 (0.19-0.22)	0.19 (0.18-0.20)
	65-69	Normotension	0.25 (0.24-0.26)	0.26 (0.25-0.27)
			0.25 (0.23-0.26)	0.26 (0.25-0.28)
		Hypertension known controlled	0.25 (0.24-0.26)	0.27 (0.26-0.28)
			0.25 (0.24-0.27)	0.27 (0.26-0.29)
		Hypertension known uncontrolled	0.30 (0.29-0.32)	0.28 (0.27-0.30)
			0.31 (0.29-0.32)	0.28 (0.27-0.30)
		Hypertension unknown uncontrolled	0.20 (0.19-0.21)	0.18 (0.17-0.19)
			0.20 (0.18-0.21)	0.18 (0.17-0.20)
Female	20-24	Normotension	0.95 (0.94-0.96)	0.95 (0.94-0.96)
			0.96 (0.94-0.97)	0.96 (0.94-0.97)
		Hypertension known controlled	0.02 (0.01-0.03)	0.02 (0.01-0.03)
			0.02 (0.01-0.03)	0.02 (0.01-0.03)
		Hypertension known uncontrolled	0.01 (0.00-0.01)	0.01 (0.00-0.01)
			0.01 (0.00-0.01)	0.01 (0.00-0.01)
		Hypertension unknown uncontrolled	0.02 (0.01-0.03)	0.02 (0.01-0.03)
			0.02 (0.01-0.03)	0.02 (0.01-0.03)
	25-34	Normotension	0.92 (0.91-0.93)	0.92 (0.92-0.93)
			0.92 (0.91-0.93)	0.92 (0.91-0.93)
		Hypertension known controlled	0.03 (0.03-0.04)	0.03 (0.03-0.04)
			0.03 (0.03-0.04)	0.03 (0.03-0.04)
		Hypertension known uncontrolled	0.01 (0.01-0.01)	0.01 (0.01-0.01)
			0.01 (0.01-0.01)	0.01 (0.01-0.01)
		Hypertension unknown uncontrolled	0.04 (0.03-0.04)	0.03 (0.03-0.04)
			0.04 (0.03-0.04)	0.04 (0.03-0.05)
	35-44	Normotension	0.83 (0.82-0.84)	0.83 (0.83-0.84)
			0.83 (0.82-0.85)	0.83 (0.82-0.85)
		Hypertension known controlled	0.06 (0.06-0.07)	0.07 (0.06-0.07)
			0.07 (0.06-0.07)	0.07 (0.06-0.07)
		Hypertension known uncontrolled	0.03 (0.03-0.04)	0.03 (0.03-0.04)
			0.03 (0.03-0.04)	0.03 (0.03-0.04)
		Hypertension unknown uncontrolled	0.07 (0.06-0.07)	0.07 (0.06-0.07)
			0.07 (0.06-0.08)	0.07 (0.06-0.09)
	45-54	Normotension	0.68 (0.67-0.69)	0.69 (0.68-0.69)
			0.67 (0.65-0.68)	0.67 (0.66-0.68)
		Hypertension known controlled	0.12 (0.11-0.12)	0.12 (0.12-0.13)
			0.12 (0.12-0.13)	0.13 (0.12-0.13)
		Hypertension known uncontrolled	0.08 (0.08-0.09)	0.08 (0.07-0.08)
			0.09 (0.08-0.09)	0.08 (0.08-0.09)
		Hypertension unknown uncontrolled	0.12 (0.11-0.12)	0.11 (0.11-0.12)
			0.12 (0.11-0.13)	0.12 (0.11-0.13)
	55-64	Normotension	0.46 (0.46-0.47)	0.48 (0.47-0.48)
			0.46 (0.44-0.48)	0.47 (0.45-0.48)
		Hypertension known controlled	0.21 (0.21-0.22)	0.23 (0.22-0.23)
			0.21 (0.20-0.22)	0.22 (0.21-0.23)
		Hypertension known uncontrolled	0.17 (0.16-0.18)	0.16 (0.15-0.16)
			0.18 (0.16-0.19)	0.16 (0.15-0.18)
		Hypertension unknown uncontrolled	0.15 (0.15-0.16)	0.14 (0.14-0.15)
			0.15 (0.14-0.17)	0.15 (0.14-0.16)
	65-69	Normotension	0.32 (0.31-0.33)	0.33 (0.32-0.34)
			0.32 (0.30-0.34)	0.33 (0.32-0.35)
		Hypertension known controlled	0.24 (0.23-0.25)	0.27 (0.26-0.28)
			0.24 (0.23-0.26)	0.26 (0.25-0.28)
		Hypertension known uncontrolled	0.26 (0.25-0.27)	0.24 (0.23-0.25)
			0.26 (0.25-0.28)	0.24 (0.22-0.25)
		Hypertension unknown uncontrolled	0.18 (0.17-0.19)	0.16 (0.16-0.17)
			0.18 (0.16-0.19)	0.16 (0.15-0.17)
**MASD:** 0.837; **CIO:** 0.768				

Hypertension status by sex and age group using different definitions for the blood pressure values for scientific evaluations. Percentile-based 95% bootstrap confidence intervals are reported for original data results. Median and percentile-based 95% confidence intervals of synthetic data results are printed in orange. avg., average; meas., measurement; MASD, mean absolute standardised difference; CIO, confidence interval overlap

B.2 Fischer et al.[3]

Table S4. Replication of Table 1

Variable	Sex	Age group	n	Mean	SD	10th percentile	50th perecentile	90th percentile
Height	Male	20-29	2899 (2798-2999)	180.75 (180.50-181.01)	7.14 (6.90-7.38)	171.90 (171.50-172.40)	180.70 (180.40-181.10)	189.90 (189.50-190.20)
			2210 (2044-2462)	180.19 (179.58-180.71)	7.31 (6.96-7.76)	170.98 (169.76-171.80)	180.30 (179.70-180.90)	189.38 (188.70-189.95)
		30-39	4168 (4048-4296)	180.66 (180.45-180.88)	7.15 (7.00-7.30)	171.50 (171.10-171.80)	180.60 (180.30-180.80)	189.60 (189.21-190.00)
			4933 (4572-5155)	179.95 (179.16-180.40)	7.47 (7.18-8.05)	170.40 (168.90-171.20)	180.00 (179.40-180.50)	189.30 (188.90-189.70)
		40-49	9431 (9241-9621)	179.83 (179.69-179.97)	7.14 (7.01-7.29)	170.80 (170.60-171.10)	179.90 (179.70-180.00)	188.80 (188.50-189.00)
			9799 (93312-10253)	179.17 (178.34-179.62)	7.45 (7.17-8.01)	169.70 (168.10-170.50)	179.30 (178.60-179.70)	188.50 (188.20-188.90)
		50-59	12750 (12541-12961)	178.46 (178.34-178.59)	7.11 (6.99-7.28)	169.60 (169.44-169.90)	178.30 (178.20-178.40)	187.50 (187.30-187.70)
			13790 (13094-14303)	177.75 (177.14-178.10)	7.36 (7.12-7.79)	168.50 (167.30-169.10)	177.80 (177.30-178.10)	187.10 (186.80-187.40)
		60-69	14097 (13883-14312)	175.76 (175.65-175.87)	6.71 (6.63-6.79)	167.40 (167.10-167.50)	175.70 (175.50-175.80)	184.40 (184.20-184.50)
			13083 (12426-13694)	175.51 (175.23-175.75)	7.02 (6.83-7.31)	166.70 (166.00-167.10)	175.50 (175.20-175.70)	184.46 (184.20-184.70)
		70+	1449 (1375-1524)	174.17 (173.84-174.51)	6.25 (6.01-6.49)	166.38 (165.90-166.95)	174.20 (173.70-174.50)	181.92 (181.40-182.60)
			1007 (889-1122)	173.94 (173.36-174.59)	6.67 (6.24-7.21)	165.56 (164.32-166.56)	174.00 (173.40-174.70)	182.30 (181.50-183.20)
	Female	20-29	3853 (3736-3978)	167.37 (167.15-167.57)	6.67 (6.40-7.01)	159.12 (158.70-159.60)	167.20 (167.00-167.50)	175.88 (175.30-176.20)
			2956 (2721-3246)	167.76 (167.43-168.11)	6.75 (6.48-7.09)	159.40 (158.90-159.80)	167.60 (167.20-167.90)	176.40 (175.71-177.00)
		30-39	4937 (4802-5073)	167.13 (166.96-167.31)	6.50 (6.38-6.63)	158.80 (158.50-159.20)	167.00 (166.80-167.30)	175.60 (175.40-175.90)
			6045 (5647-6358)	167.46 (167.19-167.77)	6.83 (6.61-7.15)	158.90 (158.60-159.29)	167.30 (167.00-167.60)	176.20 (175.80-176.90)
		40-49	11605 (11409-11800)	166.77 (166.66-166.88)	6.56 (6.47-6.65)	158.50 (158.40-158.72)	166.70 (166.60-166.90)	175.10 (174.90-175.30)
			11797 (11251-12286)	166.90 (166.67-167.23)	6.84 (6.62-7.15)	158.40 (158.10-158.60)	166.75 (166.50-167.00)	175.60 (175.20-176.40)
		50-59	15180 (14956-15400)	165.33 (165.22-165.43)	6.46 (6.39-6.53)	157.20 (157.00-157.30)	165.20 (165.10-165.30)	173.60 (173.40-173.80)
			15956 (15366-16604)	165.46 (165.20-165.96)	6.82 (6.59-7.24)	157.00 (156.70-157.20)	165.30 (165.00-165.60)	174.20 (173.70-175.30)
		60-69	14666 (14444-14884)	162.39 (162.29-162.49)	6.19 (6.11-6.26)	154.70 (154.50-154.80)	162.30 (162.10-162.40)	170.30 (170.10-170.50)
			13916 (13387-14361)	163.05 (162.69-163.76)	6.68 (6.35-7.27)	154.82 (154.60-155.10)	162.80 (162.40-163.30)	171.60 (170.90-173.00)
		70+	1335 (1264-1405)	161.27 (160.93-161.60)	6.23 (5.90-6.68)	153.54 (153.00-154.00)	161.10 (160.70-161.50)	169.00 (168.59-169.60)
			949 (836-1076)	162.23 (161.47-163.45)	6.61 (5.96-7.72)	154.21 (153.50-155.00)	161.90 (161.15-162.80)	170.60 (169.10-173.54)
Weight	Male	20-29	2899 (2798-2999)	81.02 (80.50-81.54)	14.68 (14.06-15.32)	65.30 (64.60-65.90)	78.80 (78.30-79.50)	99.70 (98.10-101.04)
			2209 (2044-2463)	80.18 (79.43-80.93)	13.96 (13.26-14.67)	64.72 (63.76-65.59)	78.28 (77.51-79.07)	97.90 (96.24-99.68)
		30-39	4168 (4048-4296)	84.77 (84.34-85.24)	14.64 (14.19-15.12)	68.40 (67.90-68.80)	82.70 (82.30-83.20)	103.90 (102.80-104.90)
			4934 (4572-5153)	84.00 (83.25-84.62)	14.85 (14.35-15.39)	67.34 (66.22-68.09)	82.13 (81.43-82.75)	103.03 (101.87-104.34)
		40-49	9431 (9241-9621)	87.60 (87.31-87.91)	15.02 (14.68-15.37)	70.90 (70.60-71.30)	85.70 (85.30-85.90)	106.20 (105.40-106.90)
			9799 (9334-10254)	86.84 (86.01-87.36)	15.21 (14.81-15.66)	69.67 (68.35-70.44)	85.00 (84.33-85.53)	105.97 (105.08-106.90)
		50-59	12750 (12541-12961)	88.06 (87.79-88.32)	15.15 (14.87-15.42)	71.00 (70.70-71.40)	86.10 (85.80-86.30)	107.31 (106.80-107.94)
			13791 (13093-14306)	87.82 (87.13-88.30)	15.34 (15.00-15.71)	70.46 (69.34-71.09)	85.94 (85.38-86.40)	107.47 (106.65-108.32)
		60-69	14097 (13883-14312)	87.78 (87.53-88.02)	14.78 (14.54-15.03)	71.10 (70.80-71.40)	85.90 (85.60-86.20)	107.00 (106.30-107.60)
			13081 (12428-13693)	87.71 (87.23-88.11)	14.81 (14.52-15.19)	70.69 (69.80-71.26)	85.96 (85.53-86.43)	106.98 (106.26-107.68)
		70+	1449 (1375-1524)	85.44 (84.72-86.13)	13.66 (12.88-14.53)	69.56 (68.40-70.60)	84.20 (83.40-84.80)	101.92 (100.12-103.80)
			1007 (889-1124)	85.60 (84.42-86.67)	13.46 (12.56-14.51)	69.86 (67.99-71.30)	84.38 (83.22-85.48)	102.35 (100.18-104.83)
	Female	20-29	3853 (3736-3978)	66.15 (65.73-66.58)	13.56 (13.04-14.07)	52.60 (52.00-53.00)	63.40 (63.00-63.80)	83.30 (81.86-84.90)
			2956 (2721-3251)	66.20 (65.45-67.05)	13.26 (12.54-14.03)	52.67 (52.10-53.19)	63.60 (62.91-64.47)	83.08 (81.10-85.35)
		30-39	4937 (4802-5073)	68.79 (68.37-69.21)	14.93 (14.45-15.42)	53.80 (53.50-54.11)	65.60 (65.10-65.90)	87.64 (86.40-89.24)
			6047 (5647-6353)	68.99 (68.44-69.70)	14.78 (14.25-15.29)	53.86 (53.48-54.23)	65.91 (65.32-66.72)	88.23 (86.82-89.92)
		40-49	11605 (11409-11800)	71.27 (70.98-71.54)	15.26 (14.94-15.56)	55.50 (55.30-55.80)	67.90 (67.70-68.30)	91.60 (90.70-92.40)
			11796 (11250-12295)	71.54 (71.09-72.16)	15.35 (15.01-15.72)	55.46 (55.14-55.78)	68.38 (67.83-69.18)	91.97 (90.95-93.43)
		50-59	15180 (14956-15400)	72.49 (72.26-72.74)	15.49 (15.22-15.76)	56.00 (55.80-56.30)	69.30 (69.10-69.60)	92.80 (92.10-93.50)
			15958 (15367-16608)	73.07 (72.63-73.73)	15.57 (15.28-15.91)	56.26 (55.95-56.57)	70.12 (69.55-70.98)	93.75 (92.87-94.88)
		60-69	14667 (14446-14884)	72.70 (72.46-72.93)	14.55 (14.34-14.75)	56.20 (55.90-56.40)	70.50 (70.20-70.80)	91.90 (91.40-92.40)
			13915 (13391-14359)	73.42 (72.92-74.02)	14.77 (14.46-15.12)	56.64 (56.26-57.00)	71.18 (70.58-71.91)	93.02 (92.07-94.21)
		70+	1334 (1263-1404)	72.22 (71.48-72.94)	13.82 (13.08-14.53)	56.10 (55.08-56.96)	70.60 (69.90-71.40)	89.74 (88.17-91.75)
			949 (837-1078)	73.05 (71.91-74.49)	13.59 (12.70-14.60)	57.19 (55.99-58.35)	71.34 (70.06-72.88)	90.83 (88.66-93.78)
BMI	Male	20-29	2899 (2798-2999)	24.77 (24.63-24.91)	4.11 (3.91-4.31)	20.48 (20.30-20.62)	24.13 (23.97-24.28)	29.89 (29.52-30.35)
			2193 (2025-2444)	24.69 (24.47-24.91)	4.06 (3.81-4.70)	20.34 (20.10-20.56)	24.08 (23.87-24.29)	29.75 (29.24-30.32)
		30-39	4168 (4048-4296)	25.95 (25.83-26.08)	4.07 (3.94-4.21)	21.57 (21.43-21.68)	25.27 (25.14-25.43)	31.27 (30.92-31.50)
			4893 (4534-5115)	25.94 (25.77-26.12)	4.33 (4.14-4.61)	21.26 (21.02-21.44)	25.28 (25.12-25.45)	31.40 (31.00-31.89)
		40-49	9431 (9241-9621)	27.08 (26.99-27.17)	4.38 (4.24-4.56)	22.50 (22.41-22.58)	26.40 (26.31-26.51)	32.32 (32.14-32.54)
			9723 (9258-10153)	27.05 (26.92-27.18)	4.53 (4.37-4.78)	22.15 (21.89-22.33)	26.39 (26.27-26.52)	32.70 (32.41-33.14)
		50-59	12750 (12541-12961)	27.64 (27.56-27.73)	4.58 (4.34-4.98)	22.78 (22.68-22.86)	26.99 (26.92-27.08)	33.26 (33.04-33.42)
			13660 (12987-14159)	27.80 (27.69-27.93)	4.66 (4.53-4.89)	22.68 (22.43-22.83)	27.15 (27.03-27.28)	33.71 (33.46-34.10)
		60-69	14097 (13883-14312)	28.40 (28.33-28.47)	4.43 (4.36-4.51)	23.47 (23.37-23.54)	27.80 (27.71-27.89)	34.15 (33.97-34.34)
			12941 (12262-13577)	28.48 (28.36-28.59)	4.61 (4.48-4.82)	23.32 (23.06-23.48)	27.88 (27.76-28.02)	34.42 (34.17-34.70)
		70+	1449 (1375-1524)	28.16 (27.93-28.37)	4.23 (3.97-4.54)	23.51 (23.27-23.85)	27.54 (27.25-27.82)	33.49 (33.06-34.04)
			993 (877-1107)	28.30 (27.97-28.63)	4.30 (3.98-4.71)	23.47 (22.94-23.92)	27.78 (27.43-28.14)	33.79 (33.08-34.58)
	Female	20-29	3853 (3736-3978)	23.64 (23.46-23.83)	5.74 (4.41-7.66)	19.23 (19.13-19.32)	22.51 (22.36-22.66)	29.42 (29.03-30.02)
			2927 (2692-3213)	23.52 (23.28-23.80)	4.51 (4.22-5.38)	19.15 (18.99-19.29)	22.51 (22.29-22.76)	29.18 (28.51-29.94)
		30-39	4937 (4802-5073)	24.62 (24.47-24.76)	5.17 (5.00-5.35)	19.67 (19.59-19.78)	23.31 (23.15-23.44)	31.36 (30.90-31.87)
			5985 (5582-6280)	24.60 (24.42-24.81)	5.11 (4.91-5.47)	19.60 (19.47-19.71)	23.41 (23.23-23.64)	31.26 (30.76-31.80)
		40-49	11605 (11409-11800)	25.63 (25.53-25.73)	5.36 (5.25-5.47)	20.30 (20.24-20.38)	24.33 (24.24-24.43)	32.80 (32.50-33.06)
			11681 (11147-12158)	25.69 (25.55-25.88)	5.42 (5.28-5.62)	20.20 (20.09-20.30)	24.47 (24.32-24.69)	32.91 (32.55-33.35)
		50-59	15180 (14956-15400)	26.53 (26.45-26.62)	5.54 (5.44-5.63)	20.81 (20.74-20.88)	25.30 (25.20-25.41)	33.89 (33.65-34.10)
			15799 (15232-16382)	26.71 (26.57-26.86)	5.63 (5.52-5.79)	20.77 (20.65-20.88)	25.56 (25.39-25.76)	34.22 (33.92-34.56)
		60-69	14666 (14444-14884)	27.59 (27.50-27.67)	5.45 (5.37-5.54)	21.53 (21.44-21.61)	26.70 (26.59-26.80)	34.87 (34.66-35.10)
			13771 (13187-14245)	27.64 (27.52-27.77)	5.51 (5.40-5.66)	21.49 (21.34-21.61)	26.75 (26.59-26.94)	34.99 (34.72-35.32)

		70+	1334 (1263-1404)	27.78 (27.49-28.08)	5.30 (5.01-5.59)	21.84 (21.56-22.12)	26.95 (26.60-27.22)	34.74 (34.01-35.36)
			935 (826-1064)	27.78 (27.38-28.21)	5.16 (4.82-5.53)	21.89 (21.41-22.35)	27.08 (26.61-27.57)	34.55 (33.75-35.45)
Waist circumference	Male	20-29	2986 (2881-3089)	86.22 (85.85-86.61)	10.94 (10.49-11.36)	75.10 (74.60-75.30)	84.10 (83.70-84.50)	100.50 (99.25-101.32)
			2271 (2103-2536)	86.13 (85.57-86.74)	10.63 (10.06-11.17)	74.87 (74.30-75.40)	84.20 (83.60-84.90)	100.00 (98.60-101.40)
		30-39	4289 (4168-4417)	90.82 (90.48-91.17)	11.14 (10.81-11.49)	78.50 (78.10-78.85)	89.00 (88.70-89.40)	105.40 (104.50-106.20)
			5074 (4705-5297)	90.94 (90.49-91.45)	11.61 (11.23-12.01)	77.90 (77.40-78.40)	89.30 (88.80-89.90)	106.20 (105.10-107.20)
		40-49	9719 (9529-9907)	95.70 (95.46-95.93)	11.76 (11.54-11.99)	82.30 (82.00-82.60)	94.20 (94.00-94.50)	110.92 (110.30-111.30)
			10117 (9638-10587)	95.48 (95.04-95.87)	12.14 (11.82-12.50)	81.40 (80.70-82.00)	94.20 (93.70-94.60)	111.20 (110.50-112.00)
		50-59	13208 (12993-13424)	98.94 (98.72-99.14)	12.32 (12.09-12.55)	84.60 (84.40-85.00)	97.70 (97.40-98.00)	114.90 (114.40-115.20)
			14314 (13594-14855)	98.93 (98.39-99.33)	12.59 (12.32-12.92)	84.10 (83.20-84.70)	97.70 (97.30-98.10)	115.20 (114.61-115.90)
		60-69	14807 (14590-15025)	102.44 (102.23-102.64)	12.35 (12.15-12.56)	88.00 (87.70-88.20)	101.30 (101.10-101.50)	118.54 (118.20-119.00)
			13732 (13053-14353)	101.95 (101.28-102.36)	12.42 (12.15-12.82)	87.10 (85.90-87.70)	101.00 (100.40-101.40)	118.10 (117.50-118.70)
		70+	1531 (1457-1609)	102.33 (101.76-102.89)	11.66 (11.17-12.17)	88.50 (87.50-89.47)	101.30 (100.60-102.00)	117.50 (115.73-119.02)
			1066 (946-1186)	101.81 (100.70-102.69)	11.53 (10.83-12.38)	88.00 (85.76-89.42)	101.00 (99.90-102.00)	116.67 (115.00-118.40)
	Female	20-29	3966 (3846-4089)	77.12 (76.78-77.49)	11.01 (10.62-11.39)	66.00 (65.60-66.20)	74.60 (74.30-75.00)	91.50 (90.30-92.44)
			3047 (2808-3349)	77.59 (76.99-78.39)	10.82 (10.26-11.43)	66.46 (66.00-66.90)	75.30 (74.70-76.10)	91.80 (90.24-93.80)
		30-39	5126 (4988-5264)	80.96 (80.63-81.30)	12.10 (11.77-12.45)	68.40 (68.20-68.70)	78.30 (78.00-78.70)	97.30 (96.40-98.10)
			6262 (5853-6579)	81.31 (80.76-82.18)	12.23 (11.83-12.70)	68.40 (68.10-68.80)	78.80 (78.20-79.70)	98.00 (96.70-99.70)
		40-49	11982 (11781-12175)	84.11 (83.88-84.34)	12.81 (12.59-13.03)	70.30 (70.10-70.42)	81.70 (81.30-81.90)	101.40 (100.86-102.00)
			12209 (11641-12728)	84.84 (84.39-85.60)	13.08 (12.77-13.44)	70.50 (70.20-70.80)	82.40 (81.80-83.40)	102.70 (101.80-104.20)
		50-59	15740 (15514-15962)	87.82 (87.60-88.04)	13.66 (13.44-13.87)	72.40 (72.20-72.60)	85.70 (85.40-86.00)	106.50 (106.00-107.00)
			16552 (15958-17212)	88.40 (88.03-88.96)	13.70 (13.48-13.94)	72.60 (72.30-72.90)	86.40 (85.90-87.10)	107.10 (106.40-107.90)
		60-69	15358 (15141-15582)	91.20 (90.99-91.40)	13.31 (13.15-13.47)	75.00 (74.80-75.22)	89.80 (89.50-90.10)	109.10 (108.70-109.50)
			14541 (13981-15018)	91.43 (91.07-91.80)	13.37 (13.16-13.61)	75.20 (74.80-75.60)	90.10 (89.70-90.50)	109.40 (108.80-110.00)
		70+	1410 (1337-1483)	92.27 (91.60-92.93)	12.68 (12.15-13.14)	76.50 (75.80-77.64)	91.20 (90.30-92.10)	108.50 (107.02-110.00)
			1004 (891-1137)	92.45 (91.47-93.41)	12.43 (11.78-13.13)	77.02 (75.77-78.30)	91.60 (90.40-92.80)	108.60 (106.90-110.46)
Hip circumference	Male	20-29	818 (764-875)	98.93 (98.36-99.52)	8.60 (7.79-9.49)	89.44 (88.90-90.38)	98.00 (97.40-98.70)	108.93 (107.40-110.80)
			611 (546-695)	98.87 (98.14-99.54)	7.90 (7.19-8.78)	90.01 (89.09-90.92)	98.10 (97.30-98.90)	108.30 (106.71-110.00)
		30-39	1155 (1087-1223)	100.55 (100.10-101.03)	7.76 (7.33-8.22)	91.60 (91.01-92.30)	99.80 (99.20-100.20)	110.30 (109.40-111.40)
			1345 (1237-1437)	100.51 (99.99-101.05)	8.04 (7.51-8.87)	91.60 (91.00-92.19)	99.60 (99.10-100.20)	110.25 (109.10-111.60)
		40-49	2444 (2347-2539)	101.41 (101.08-101.72)	7.87 (7.38-8.32)	93.00 (92.60-93.30)	100.50 (100.20-100.90)	110.87 (109.90-111.56)
			2604 (2444-2775)	101.54 (101.15-101.95)	8.11 (7.66-8.96)	92.80 (92.30-93.20)	100.60 (100.20-101.10)	111.20 (110.30-112.10)
		50-59	3447 (3332-3563)	101.39 (101.10-101.68)	8.41 (7.44-9.85)	93.00 (92.60-93.20)	100.40 (100.10-100.80)	110.44 (110.00-111.10)
			3751 (3548-3929)	101.91 (101.58-102.26)	8.27 (7.79-9.13)	93.10 (92.80-93.50)	100.90 (100.50-101.20)	111.65 (110.90-112.50)
		60-69	3875 (3753-4001)	101.97 (101.71-102.23)	8.11 (7.76-8.47)	93.60 (93.20-93.90)	101.00 (100.70-101.20)	111.70 (111.10-112.20)
			3552 (3365-3758)	102.28 (101.96-102.60)	8.16 (7.76-8.85)	93.60 (93.30-94.00)	101.20 (100.80-101.50)	112.10 (111.40-112.90)
		70+	343 (309-381)	101.13 (100.43-101.88)	7.03 (6.19-7.90)	93.02 (92.20-94.46)	100.20 (99.60-100.90)	109.76 (108.00-111.94)
			240 (198-285)	101.69 (100.75-102.71)	7.14 (6.18-8.43)	93.80 (92.45-95.10)	100.80 (99.80-101.90)	110.42 (108.39-112.90)
	Female	20-29	1099 (1034-1166)	98.70 (98.20-99.20)	8.71 (8.12-9.31)	89.40 (89.00-90.00)	97.70 (97.00-98.15)	109.10 (108.10-110.90)
			828 (738-933)	98.51 (97.85-99.20)	8.47 (7.80-9.25)	89.40 (88.76-90.07)	97.35 (96.70-98.10)	108.90 (107.48-110.70)
		30-39	1380 (1314-1451)	100.98 (100.40-101.54)	10.55 (9.92-11.18)	90.00 (89.70-90.40)	99.00 (98.30-99.60)	114.00 (112.60-115.20)
			1693 (1551-1810)	100.34 (99.84-100.88)	9.80 (9.26-10.41)	89.90 (89.40-90.40)	98.70 (98.20-99.30)	112.60 (111.40-114.00)
		40-49	3183 (3077-3291)	102.22 (101.84-102.59)	10.46 (10.04-10.90)	90.90 (90.50-91.20)	100.40 (100.00-100.90)	115.80 (114.60-116.60)
			3304 (3097-3474)	101.97 (101.57-102.38)	10.33 (9.90-10.82)	90.80 (90.40-91.23)	100.30 (99.90-100.80)	115.20 (114.16-116.23)
		50-59	4431 (4307-4561)	102.80 (102.48-103.11)	10.70 (10.37-11.03)	91.20 (90.90-91.53)	101.10 (100.80-101.40)	116.40 (115.70-117.30)
			4626 (4414-4823)	102.90 (102.51-103.28)	10.59 (10.19-11.10)	91.40 (91.00-91.80)	101.20 (100.80-101.60)	116.60 (115.60-117.50)
		60-69	4324 (4200-4456)	103.55 (103.23-103.85)	10.58 (10.30-10.86)	91.60 (91.40-92.09)	101.80 (101.40-102.20)	117.60 (116.97-118.30)
			4086 (3885-4263)	103.51 (103.15-103.89)	10.43 (10.10-10.84)	91.90 (91.50-92.23)	101.80 (101.40-102.30)	117.30 (116.50-118.20)
		70+	391 (354-430)	102.99 (101.94-104.12)	10.66 (9.69-11.64)	91.30 (90.50-92.72)	101.20 (100.00-102.30)	117.50 (114.80-120.00)
			263 (221-314)	103.28 (101.97-104.63)	9.96 (8.81-11.17)	92.20 (90.70-93.66)	101.70 (100.30-103.30)	116.20 (113.28-119.58)
**MASD:** 3.320; **CIO:** 0.512								

Distribution of anthropometric measurement parameters by sex and age group (in years). Percentile-based 95% bootstrap confidence intervals are reported for original data results. Median and percentile-based 95% confidence intervals of synthetic data results are printed in orange. n, number of participants; SD, standard deviation; BMI, body mass index; MASD, mean absolute standardised difference; CIO, confidence interval overlap

Table S5. Replication of Figure 2

Study centre	Height men (mean)	Height women (mean)
Augsburg	176.83 (176.63-177.03)	163.66 (163.48-163.84)
	176.64 (176.19-176.96)	164.34 (164.03-164.90)
Berlin Mitte	178.17 (177.90-178.45)	165.25 (165.02-165.47)
	177.67 (177.05-178.12)	165.53 (165.19-166.06)
Berlin Nord	177.60 (177.30-177.89)	164.70 (164.47-164.92)
	177.34 (176.70-177.77)	165.20 (164.84-165.86)
Berlin Süd	178.53 (178.24-178.84)	165.58 (165.32-165.83)
	177.98 (177.26-178.43)	165.83 (165.48-166.41)
Bremen	179.62 (179.34-179.92)	166.15 (165.91-166.39)
	178.93 (178.00-179.38)	166.40 (166.04-166.94)
Düsseldorf	177.99 (177.53-178.42)	164.94 (164.60-165.29)
	177.54 (176.77-178.13)	165.36 (164.90-166.05)
Essen	177.82 (177.48-178.15)	165.06 (164.77-165.34)
	177.41 (176.66-177.86)	165.35 (164.95-166.02)
Freiburg	177.59 (177.31-177.89)	164.99 (164.73-165.25)
	177.30 (176.62-177.74)	165.47 (165.08-166.07)
Halle	177.34 (177.06-177.63)	164.33 (164.10-164.58)
	176.99 (176.15-177.43)	164.84 (164.44-165.64)
Hamburg	179.21 (178.83-179.60)	165.76 (165.42-166.10)
	178.35 (177.70-178.86)	166.12 (165.62-166.70)
Hannover	179.26 (178.99-179.55)	166.06 (165.79-166.32)
	178.55 (177.77-179.02)	166.21 (165.82-166.72)
Kiel	179.41 (179.07-179.75)	166.24 (165.94-166.53)
	178.68 (177.97-179.21)	166.37 (165.97-166.90)
Leipzig	177.51 (177.27-177.75)	164.98 (164.76-165.18)
	177.18 (176.59-177.58)	165.31 (164.97-165.85)
Mannheim	177.70 (177.32-178.03)	164.52 (164.21-164.82)
	177.23 (176.70-177.68)	165.10 (164.68-165.62)
Münster	180.56 (180.30-180.85)	167.11 (166.84-167.37)
	179.78 (178.85-180.27)	167.24 (166.81-167.80)
Neubrandenburg	178.12 (177.94-178.29)	164.86 (164.71-165.03)
	177.76 (177.23-178.14)	165.34 (165.03-165.87)
Regensburg	177.51 (177.21-177.83)	164.07 (163.78-164.34)
	177.34 (176.66-177.79)	164.78 (164.36-165.51)
Saarbrücken	176.98 (176.65-177.29)	163.77 (163.52-164.02)
	176.82 (176.08-177.27)	164.32 (163.92-165.03)
**MASD:** 3.090; **CIO:** 0.378		

Mean height (in cm) of participants by study centre and sex. Percentile-based 95% bootstrap confidence intervals are reported for original data results. Median and percentile-based 95% confidence intervals of synthetic data results are printed in orange; MASD, mean absolute standardised difference; CIO, confidence interval overlap

Table S6. Replication of Figure 3

Sex	Age group	BMI group	Proportion
Male	20-29	Overweight	0.30 (0.28-0.31)
			0.30 (0.28-0.32)
		Obese	0.10 (0.09-0.11)
			0.09 (0.08-0.11)
	30-39	Overweight	0.39 (0.38-0.41)
			0.38 (0.37-0.40)
		Obese	0.14 (0.13-0.15)
			0.15 (0.13-0.16)
	40-49	Overweight	0.47 (0.46-0.48)
			0.44 (0.42-0.46)
		Obese	0.20 (0.19-0.21)
			0.21 (0.20-0.22)
	50-59	Overweight	0.47 (0.47-0.48)
			0.46 (0.44-0.47)
		Obese	0.24 (0.23-0.25)
			0.26 (0.25-0.28)
	60-69	Overweight	0.49 (0.48-0.50)
			0.47 (0.45-0.48)
		Obese	0.30 (0.29-0.31)
			0.32 (0.30-0.33)
	70+	Overweight	0.52 (0.49-0.54)
			0.50 (0.46-0.53)
		Obese	0.27 (0.25-0.30)
			0.29 (0.26-0.33)
Female	20-29	Overweight	0.18 (0.17-0.19)
			0.19 (0.17-0.21)
		Obese	0.09 (0.08-0.10)
			0.08 (0.07-0.10)
	30-39	Overweight	0.22 (0.21-0.23)
			0.23 (0.22-0.25)
		Obese	0.13 (0.12-0.14)
			0.13 (0.12-0.14)
	40-49	Overweight	0.27 (0.26-0.28)
			0.28 (0.27-0.29)
		Obese	0.17 (0.17-0.18)
			0.18 (0.17-0.19)
	50-59	Overweight	0.31 (0.30-0.32)
			0.31 (0.31-0.32)
		Obese	0.22 (0.21-0.22)
			0.23 (0.22-0.24)
	60-69	Overweight	0.35 (0.34-0.36)
			0.35 (0.34-0.36)
		Obese	0.28 (0.28-0.29)
			0.29 (0.28-0.30)
	70+	Overweight	0.38 (0.36-0.41)
			0.39 (0.35-0.42)
		Obese	0.29 (0.26-0.31)
			0.29 (0.26-0.33)
**MASD:** 2.052; **CIO:** 0.569			

Proportion of overweight and obese study participants by sex and age group (in years). Percentile-based 95% bootstrap confidence intervals are reported for original data results. Median and percentile-based 95% confidence intervals of synthetic data results are printed in orange. BMI, body mass index; MASD, mean absolute standardised difference; CIO, confidence interval overlap

Figure S3. Replication of Figure 3

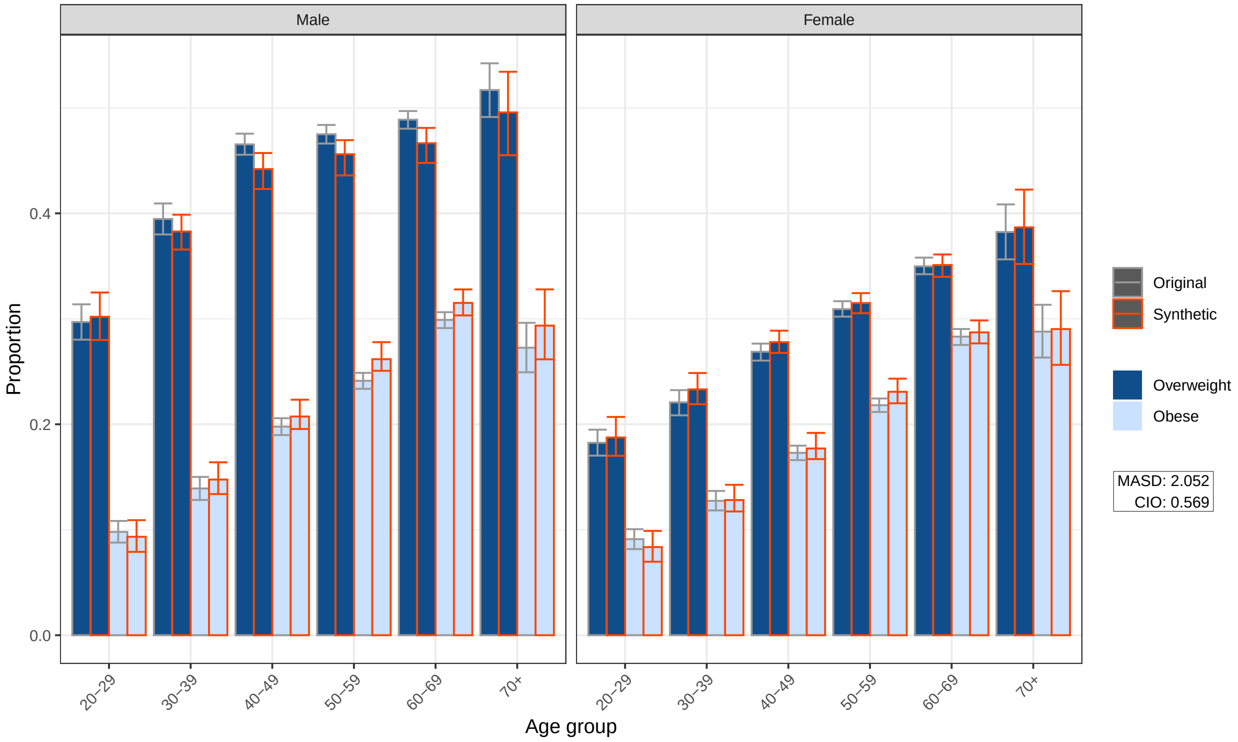

Proportion of overweight and obese study participants by sex and age group (in years). Percentile-based 95% bootstrap confidence intervals are reported for original data results. Median and percentile-based 95% confidence intervals of synthetic data results are reported. BMI, body mass index; MASD, mean absolute standardised difference; CIO, confidence interval overlap

Table S7. Replication of Figure 4

Study centre	BMI men (mean)	BMI women (mean)
Augsburg	27.91 (27.78-28.05)	26.81 (26.66-26.97)
	27.93 (27.77-28.09)	26.84 (26.66-27.03)
Berlin Mitte	26.74 (26.59-26.90)	25.45 (25.27-25.62)
	26.90 (26.70-27.12)	25.57 (25.36-25.81)
Berlin Nord	27.71 (27.53-27.90)	26.41 (26.23-26.60)
	27.65 (27.45-27.86)	26.44 (26.23-26.66)
Berlin Süd	26.93 (26.75-27.11)	25.46 (25.29-25.65)
	27.03 (26.80-27.25)	25.61 (25.41-25.85)
Bremen	26.74 (26.58-26.92)	25.62 (25.44-25.80)
	26.82 (26.63-27.02)	25.72 (25.51-25.94)
Düsseldorf	27.21 (26.92-27.56)	26.13 (25.86-26.41)
	27.21 (26.93-27.50)	26.20 (25.89-26.51)
Essen	27.95 (27.75-28.16)	26.68 (26.43-26.93)
	27.99 (27.74-28.24)	26.77 (26.50-27.05)
Freiburg	26.66 (26.48-26.83)	25.16 (24.95-25.37)
	26.67 (26.47-26.91)	25.21 (24.95-25.49)
Halle	27.53 (27.34-27.70)	26.68 (26.44-26.88)
	27.64 (27.43-27.85)	26.75 (26.50-27.01)
Hamburg	27.04 (26.80-27.27)	26.06 (25.78-26.35)
	27.21 (26.93-27.49)	26.17 (25.88-26.47)
Hannover	26.75 (26.59-26.93)	25.75 (25.52-26.00)
	26.92 (26.72-27.16)	25.81 (25.59-26.06)
Kiel	27.54 (27.34-27.74)	26.51 (26.26-26.75)
	27.68 (27.42-27.93)	26.50 (26.22-26.78)
Leipzig	27.36 (27.22-27.51)	26.48 (26.31-26.65)
	27.47 (27.29-27.66)	26.57 (26.36-26.78)
Mannheim	27.33 (27.12-27.56)	26.26 (26.01-26.50)
	27.44 (27.20-27.69)	26.28 (26.02-26.54)
Münster	26.65 (26.48-26.81)	25.28 (25.08-25.47)
	26.74 (26.55-26.94)	25.47 (25.23-25.73)
Neubrandenburg	28.34 (28.23-28.47)	27.36 (27.21-27.51)
	28.32 (28.14-28.48)	27.32 (27.14-27.51)
Regensburg	27.84 (27.64-28.03)	26.44 (26.21-26.69)
	27.73 (27.51-27.98)	26.47 (26.21-26.73)
Saarbrücken	27.86 (27.67-28.06)	26.58 (26.36-26.81)
	27.82 (27.56-28.06)	26.57 (26.32-26.83)
**MASD:** 0.818; **CIO:** 0.801		

Mean BMI (in kg/m²) of participants by study centre and sex. Percentile-based 95% bootstrap confidence intervals are reported for original data results. Median and percentile-based 95% confidence intervals of synthetic data results are printed in orange. BMI, body mass index; MASD, mean absolute standardised difference; CIO, confidence interval overlap

Figure S4. Replication of Figure 5

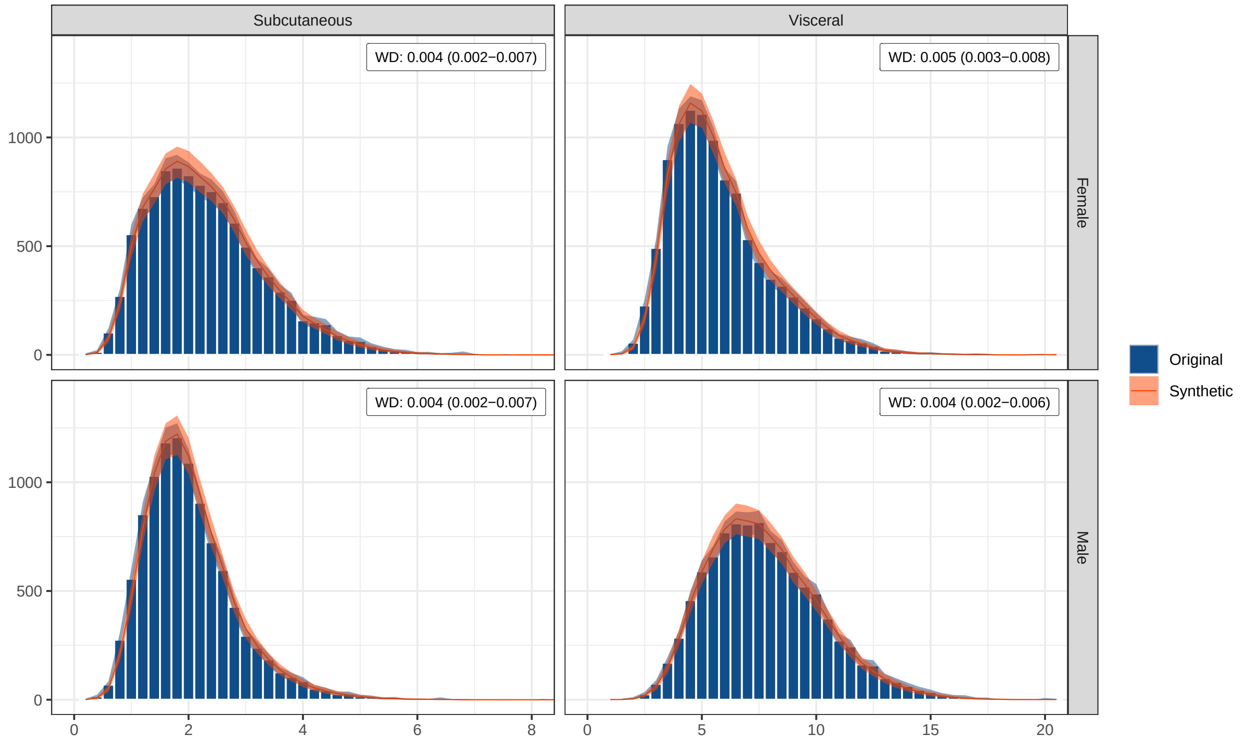

Subcutaneous and visceral abdominal adipose tissue thickness by sex. Percentile-based 95% bootstrap confidence intervals are reported for original data results. Median and percentile-based 95% confidence intervals of synthetic data results are reported. In order to provide a clear comparability of the distributions despite the variability in the distribution of missing values in the synthetic data, the counts for the synthetic results were rescaled to match the total count of the real ones; WD, Wasserstein distance

B.3 Wienbergen et al.[6]

Table S8. Replication of Table 1

Variable	Value cases	Proportion cases	Value controls	Proportion controls
Number of participants (n)	522 (485-560)		1191 (1153-1228)	
	522 (486-560)		1191 (1153-1227)	
Age (years) (median)	42 (42-43)		42 (41-42)	
	41 (41-42)		41 (40-41)	
Age (years) (1st quartile)	39 (38-39)		38 (37-38)	
	38 (38-39)		37 (37-38)	
Age (years) (3rd quartile)	44 (44-44)		44 (43-44)	
	43 (43-44)		43 (43-43)	
Sex: Male (n)	434 (399-470)	0.83 (0.80-0.86)	927 (888-967)	0.78 (0.75-0.80)
	435 (401-469)	0.83 (0.80-0.86)	927 (889-965)	0.78 (0.76-0.80)
Sex: Female (n)	88 (71-106)	0.17 (0.14-0.20)	264 (235-295)	0.22 (0.20-0.25)
	87 (71-105)	0.17 (0.14-0.20)	264 (236-292)	0.22 (0.20-0.24)
Country of birth: Other countries (n)	102 (83-122)	0.20 (0.16-0.23)	230 (204-258)	0.19 (0.17-0.22)
	104 (84-124)	0.20 (0.16-0.23)	229 (203-257)	0.19 (0.17-0.21)
Country of birth: Germany (n)	420 (386-455)	0.80 (0.77-0.84)	961 (920-1001)	0.81 (0.78-0.83)
	419 (384-455)	0.80 (0.77-0.84)	961 (921-1000)	0.81 (0.79-0.83)
School education (years): <10 (n)	38 (27-51)	0.07 (0.05-0.10)	76 (60-94)	0.06 (0.05-0.08)
	38 (27-52)	0.07 (0.05-0.10)	75 (59-93)	0.06 (0.05-0.08)
School education (years): 10-11 (n)	382 (348-414)	0.73 (0.69-0.77)	241 (212-268)	0.20 (0.18-0.22)
	371 (337-408)	0.71 (0.67-0.76)	251 (223-282)	0.21 (0.19-0.24)
School education (years): 12+ (n)	102 (82-121)	0.20 (0.16-0.23)	874 (835-918)	0.73 (0.71-0.76)
	113 (92-134)	0.21 (0.18-0.25)	864 (820-902)	0.73 (0.70-0.75)
Smoking status: Never (n)	53 (40-67)	0.10 (0.08-0.13)	580 (543-617)	0.49 (0.46-0.52)
	61 (47-78)	0.12 (0.09-0.15)	572 (532-612)	0.48 (0.45-0.51)
Smoking status: Former (n)	39 (27-51)	0.07 (0.05-0.10)	324 (294-357)	0.27 (0.25-0.30)
	46 (33-60)	0.09 (0.06-0.11)	317 (285-349)	0.27 (0.24-0.29)
Smoking status: Current (n)	430 (395-465)	0.82 (0.79-0.86)	287 (257-318)	0.24 (0.22-0.27)
	415 (382-452)	0.80 (0.76-0.83)	302 (271-334)	0.25 (0.23-0.28)
Alcohol consumption: Never (n)	112 (92-133)	0.21 (0.18-0.25)	121 (101-142)	0.10 (0.08-0.12)
	110 (91-130)	0.21 (0.18-0.25)	124 (103-144)	0.10 (0.09-0.12)
Alcohol consumption: Once a month (n)	176 (153-201)	0.34 (0.30-0.38)	193 (169-219)	0.16 (0.14-0.18)
	171 (147-197)	0.33 (0.29-0.37)	197 (172-224)	0.17 (0.15-0.19)
Alcohol consumption: 2-4 times a month (n)	130 (108-151)	0.25 (0.21-0.29)	441 (405-477)	0.37 (0.34-0.40)
	133 (113-155)	0.26 (0.22-0.29)	437 (404-473)	0.37 (0.34-0.39)
Alcohol consumption: 2-3 times a week (n)	67 (52-84)	0.13 (0.10-0.16)	302 (273-332)	0.25 (0.23-0.28)
	70 (54-86)	0.13 (0.10-0.16)	299 (269-329)	0.25 (0.23-0.28)
Alcohol consumption: 4+ times a week (n)	37 (26-49)	0.07 (0.05-0.09)	134 (112-156)	0.11 (0.09-0.13)
	38 (26-50)	0.07 (0.05-0.09)	133 (112-155)	0.11 (0.09-0.13)
Body mass index (kg/m^2) (median)	28.40 (27.76-28.88)		25.50 (25.30-25.80)	
	28.62 (28.07-29.20)		25.68 (25.38-25.97)	
Body mass index (kg/m^2) (1st quartile)	25.10 (24.84-25.51)		23.00 (22.80-23.50)	
	25.46 (24.98-25.96)		23.25 (22.98-23.55)	
Body mass index (kg/m^2) (3rd quartile)	31.79 (31.23-32.51)		28.35 (28.00-28.70)	
	32.17 (31.52-32.86)		28.63 (28.23-29.03)	
Body mass index: <25.0 (n)	123 (103-144)	0.24 (0.20-0.27)	514 (475-549)	0.43 (0.40-0.46)
	111 (92-133)	0.21 (0.18-0.25)	511 (471-549)	0.43 (0.40-0.46)
Body mass index: 25.0-29.9 (n)	208 (180-233)	0.40 (0.36-0.44)	482 (447-517)	0.40 (0.38-0.43)
	205 (178-234)	0.39 (0.35-0.44)	472 (432-511)	0.40 (0.37-0.43)
Body mass index: 30.0+ (n)	191 (165-217)	0.37 (0.33-0.41)	195 (170-220)	0.16 (0.14-0.18)
	205 (178-234)	0.39 (0.35-0.44)	209 (180-236)	0.18 (0.15-0.20)
Waist-to-hip ratio (median)	0.98 (0.97-0.99)		0.90 (0.89-0.90)	
	0.98 (0.97-0.99)		0.90 (0.89-0.90)	
Waist-to-hip ratio (1st quartile)	0.93 (0.91-0.95)		0.85 (0.84-0.86)	
	0.92 (0.91-0.94)		0.85 (0.84-0.85)	
Waist-to-hip ratio (3rd quartile)	1.03 (1.02-1.04)		0.95 (0.94-0.96)	
	1.03 (1.02-1.04)		0.95 (0.94-0.96)	
Hypertension: No (n)	391 (358-425)	0.75 (0.71-0.79)	1185 (1147-1223)	0.99 (0.99-1.00)
	398 (365-433)	0.76 (0.73-0.80)	1178 (1140-1214)	0.99 (0.98-0.99)
Hypertension: Yes (n)	131 (111-153)	0.25 (0.21-0.29)	6 (2-11)	0.01 (0.00-0.01)
	124 (103-146)	0.24 (0.20-0.27)	13 (6-21)	0.01 (0.01-0.02)
Diabetes mellitus: No (n)	461 (425-497)	0.88 (0.85-0.91)	1171 (1133-1209)	0.98 (0.98-0.99)
	464 (429-500)	0.89 (0.86-0.92)	1168 (1131-1204)	0.98 (0.97-0.99)
Diabetes mellitus: Yes (n)	61 (46-77)	0.12 (0.09-0.15)	20 (12-29)	0.02 (0.01-0.02)
	58 (44-74)	0.11 (0.08-0.14)	22 (13-32)	0.02 (0.01-0.03)
Family history of premature MI: No (n)	358 (326-391)	0.69 (0.65-0.72)	1012 (970-1052)	0.85 (0.83-0.87)
	361 (328-393)	0.69 (0.65-0.73)	1008 (968-1048)	0.85 (0.83-0.87)
Family history of premature MI: Yes (n)	144 (122-166)	0.28 (0.24-0.32)	97 (79-116)	0.08 (0.07-0.10)
	138 (116-162)	0.27 (0.23-0.30)	103 (83-124)	0.09 (0.07-0.10)
Family history of premature MI: Unknown (n)	20 (12-29)	0.04 (0.02-0.06)	82 (65-100)	0.07 (0.05-0.08)
	23 (14-32)	0.04 (0.03-0.06)	79 (63-97)	0.07 (0.05-0.08)
**MASD:** 0.664; **CIO:** 0.863				

Distribution of sociodemographic, lifestyle, and metabolic factors and family history of premature myocardial infarction according to cases and controls. Percentile-based 95% bootstrap confidence intervals are reported for original data results. Median and percentile-based 95% confidence intervals of synthetic data results are printed in orange. n, number of participants; MI, myocaridal infarction; MASD, mean absolute standardised difference; CIO, confidence interval overlap

Table S9. Replication of Table 2

Adj. level	Variable	Odds ratio	2.5%	97.5%
1	Smoking status: Former	1.00 (0.62-1.59)	0.63 (0.38-1.00)	1.60 (1.01-2.54)
		1.09 (0.68-1.66)	0.70 (0.43-1.07)	1.68 (1.08-2.58)
	Smoking status: Current	10.75 (7.87-15.80)	7.60 (5.61-10.79)	15.21 (10.98-23.13)
		8.66 (6.14-12.45)	6.25 (4.52-8.75)	12.02 (8.37-17.78)
	Alcohol consumption: 1x/month	1.04 (0.69-1.54)	0.70 (0.46-1.04)	1.53 (1.03-2.30)
		1.03 (0.70-1.55)	0.70 (0.48-1.06)	1.52 (1.02-2.28)
	Alcohol consumption: 2-4x/month	0.40 (0.27-0.59)	0.28 (0.18-0.40)	0.60 (0.40-0.87)
		0.44 (0.30-0.64)	0.30 (0.21-0.44)	0.64 (0.44-0.93)
	Alcohol consumption: 2-3x/week	0.29 (0.19-0.45)	0.19 (0.12-0.29)	0.46 (0.30-0.69)
		0.33 (0.22-0.52)	0.22 (0.14-0.34)	0.51 (0.33-0.79)
	Alcohol consumption: 4+x/week	0.38 (0.22-0.63)	0.22 (0.12-0.37)	0.64 (0.37-1.06)
		0.41 (0.25-0.67)	0.25 (0.14-0.41)	0.69 (0.42-1.12)
	BMI: 25.0-29.9	1.59 (1.19-2.12)	1.18 (0.88-1.56)	2.15 (1.61-2.88)
		1.76 (1.30-2.37)	1.31 (0.96-1.74)	2.38 (1.75-3.22)
	BMI: 30.0+	2.72 (1.94-3.84)	1.96 (1.39-2.75)	3.77 (2.69-5.36)
		3.11 (2.22-4.36)	2.25 (1.61-3.14)	4.30 (3.06-6.10)
	BMI 5 units increase	1.42 (1.25-1.63)	1.26 (1.11-1.43)	1.61 (1.41-1.85)
		1.53 (1.35-1.76)	1.35 (1.19-1.54)	1.74 (1.52-2.00)
	Waist-to-hip ratio: 0.87–0.93	1.82 (1.03-3.27)	1.05 (0.60-1.82)	3.15 (1.76-5.94)
		1.92 (1.17-3.32)	1.17 (0.72-1.95)	3.14 (1.88-5.64)
	Waist-to-hip ratio: 0.93+	9.21 (5.41-16.75)	5.47 (3.36-9.33)	15.50 (8.76-30.19)
		7.39 (4.62-12.83)	4.64 (3.01-7.61)	11.72 (7.09-21.46)
	Hypertension: Yes	65.37 (32.46-218.27)	27.51 (16.16-52.56)	155.34 (65.36-907.00)
		25.26 (12.98-63.28)	13.43 (7.65-26.95)	47.59 (22.13-151.25)
	Diabetes mellitus: Yes	6.20 (3.68-11.19)	3.38 (2.06-5.61)	11.34 (6.47-22.21)
		5.17 (2.94-9.73)	2.89 (1.69-5.13)	9.22 (5.02-18.67)
	Family history premature MI: Yes	4.05 (2.82-6.00)	2.87 (2.00-4.19)	5.71 (3.95-8.57)
		3.46 (2.41-5.00)	2.48 (1.73-3.55)	4.83 (3.32-7.05)
	Family history premature MI: Unknown	0.41 (0.21-0.67)	0.23 (0.11-0.40)	0.71 (0.41-1.14)
		0.53 (0.29-0.91)	0.31 (0.16-0.55)	0.91 (0.53-1.51)
2	Smoking status: Former	0.97 (0.57-1.63)	0.60 (0.34-1.00)	1.59 (0.96-2.67)
		1.05 (0.65-1.66)	0.67 (0.40-1.05)	1.65 (1.04-2.61)
	Smoking status: Current	12.09 (8.75-18.45)	8.38 (6.17-12.34)	17.44 (12.41-27.48)
		9.08 (6.34-13.59)	6.44 (4.56-9.33)	12.79 (8.77-19.76)
	Alcohol consumption: 1x/month	0.99 (0.63-1.57)	0.62 (0.39-0.98)	1.56 (1.01-2.50)
		1.03 (0.65-1.62)	0.66 (0.42-1.04)	1.59 (1.01-2.53)
	Alcohol consumption: 2-4x/month	0.35 (0.22-0.55)	0.22 (0.14-0.35)	0.55 (0.35-0.87)
		0.44 (0.28-0.67)	0.28 (0.18-0.44)	0.67 (0.44-1.02)
	Alcohol consumption: 2-3x/week	0.29 (0.16-0.47)	0.17 (0.10-0.28)	0.48 (0.28-0.79)
		0.36 (0.22-0.59)	0.22 (0.13-0.37)	0.58 (0.36-0.95)
	Alcohol consumption: 4+x/week	0.27 (0.14-0.48)	0.15 (0.07-0.26)	0.49 (0.26-0.89)
		0.35 (0.20-0.63)	0.20 (0.11-0.36)	0.63 (0.36-1.09)
	BMI: 25.0-29.9	1.65 (1.16-2.32)	1.16 (0.81-1.62)	2.35 (1.66-3.34)
		1.79 (1.28-2.50)	1.27 (0.91-1.76)	2.51 (1.79-3.55)
	BMI: 30.0+	3.05 (2.05-4.59)	2.07 (1.39-3.09)	4.49 (3.03-6.88)
		3.20 (2.17-4.73)	2.22 (1.51-3.23)	4.64 (3.13-6.95)
	BMI 5 units increase	1.47 (1.27-1.74)	1.28 (1.10-1.49)	1.70 (1.47-2.02)
		1.54 (1.32-1.81)	1.34 (1.15-1.55)	1.78 (1.52-2.09)
	Waist-to-hip ratio: 0.87–0.93	1.49 (0.80-2.89)	0.81 (0.44-1.50)	2.75 (1.48-5.60)
		1.61 (0.93-3.00)	0.94 (0.56-1.71)	2.77 (1.58-5.30)
	Waist-to-hip ratio: 0.93+	7.27 (4.31-14.96)	4.08 (2.49-7.60)	12.97 (7.53-28.98)
		5.79 (3.48-10.40)	3.50 (2.19-5.96)	9.60 (5.51-18.19)
	Hypertension: Yes	82.75 (35.58-365.81)	31.26 (15.29-77.73)	219.10 (80.73-1730.39)
		21.54 (9.66-59.86)	10.58 (5.32-23.97)	43.16 (17.44-151.58)
	Diabetes mellitus: Yes	5.66 (3.12-11.54)	2.73 (1.54-5.13)	11.73 (6.30-26.64)
		4.39 (2.19-9.20)	2.24 (1.15-4.33)	8.67 (4.17-20.01)
	Family history premature MI: Yes	2.92 (1.88-4.70)	1.96 (1.26-3.09)	4.36 (2.79-7.15)
		2.58 (1.73-3.95)	1.76 (1.19-2.67)	3.77 (2.51-5.83)
	Family history premature MI: Unknown	0.24 (0.11-0.45)	0.13 (0.05-0.24)	0.46 (0.22-0.83)
		0.39 (0.19-0.74)	0.21 (0.09-0.42)	0.71 (0.37-1.31)
3	Smoking status: Former	0.70 (0.37-1.25)	0.40 (0.20-0.73)	1.21 (0.69-2.15)
		0.90 (0.54-1.48)	0.55 (0.32-0.92)	1.46 (0.91-2.40)
	Smoking status: Current	11.76 (8.28-18.42)	7.94 (5.69-11.97)	17.40 (12.00-28.20)
		8.45 (5.79-12.89)	5.90 (4.14-8.69)	12.13 (8.17-19.16)
	Alcohol consumption: 1x/month	1.13 (0.70-1.82)	0.69 (0.42-1.11)	1.84 (1.15-3.04)
		1.08 (0.67-1.74)	0.68 (0.42-1.09)	1.71 (1.07-2.74)
	Alcohol consumption: 2-4x/month	0.38 (0.23-0.62)	0.24 (0.14-0.38)	0.62 (0.38-1.01)
		0.46 (0.29-0.73)	0.30 (0.19-0.47)	0.72 (0.46-1.14)
	Alcohol consumption: 2-3x/week	0.33 (0.19-0.57)	0.19 (0.10-0.33)	0.58 (0.33-0.98)
		0.38 (0.23-0.65)	0.23 (0.14-0.40)	0.64 (0.39-1.08)
	Alcohol consumption: 4+x/week	0.27 (0.13-0.50)	0.14 (0.07-0.26)	0.52 (0.27-0.97)
		0.36 (0.19-0.66)	0.20 (0.10-0.37)	0.67 (0.37-1.20)
	BMI: 25.0-29.9	1.50 (1.05-2.15)	1.03 (0.72-1.47)	2.17 (1.52-3.15)
		1.68 (1.18-2.38)	1.18 (0.83-1.66)	2.40 (1.68-3.43)
	BMI: 30.0+	2.43 (1.54-3.82)	1.60 (1.01-2.49)	3.69 (2.34-5.93)
		2.58 (1.72-3.84)	1.74 (1.17-2.58)	3.81 (2.52-5.75)
	BMI 5 units increase	1.34 (1.13-1.60)	1.15 (0.97-1.36)	1.57 (1.32-1.89)
		1.41 (1.20-1.67)	1.21 (1.04-1.42)	1.64 (1.40-1.95)
	Waist-to-hip ratio: 0.87–0.93	1.56 (0.80-3.13)	0.82 (0.41-1.55)	2.99 (1.50-6.31)
		1.57 (0.90-2.96)	0.89 (0.52-1.66)	2.75 (1.54-5.30)
	Waist-to-hip ratio: 0.93+	6.27 (3.54-13.23)	3.40 (1.93-6.69)	11.54 (6.27-26.50)
		4.96 (2.92-9.32)	2.96 (1.78-5.13)	8.35 (4.74-16.41)
	Hypertension: Yes	82.75 (35.58-365.81)	31.26 (15.29-77.73)	219.10 (80.73-1730.39)
		21.54 (9.66-59.86)	10.58 (5.32-23.97)	43.16 (17.44-151.58)
	Diabetes mellitus: Yes	5.66 (3.12-11.54)	2.73 (1.54-5.13)	11.73 (6.30-26.64)
		4.39 (2.19-9.20)	2.24 (1.15-4.33)	8.67 (4.17-20.01)
	Family history premature MI: Yes	2.76 (1.74-4.50)	1.80 (1.14-2.90)	4.22 (2.67-7.10)
		2.44 (1.61-3.81)	1.64 (1.07-2.53)	3.64 (2.38-5.73)
	Family history premature MI: Unknown	0.22 (0.10-0.42)	0.11 (0.04-0.22)	0.45 (0.22-0.83)
		0.39 (0.18-0.75)	0.20 (0.09-0.42)	0.74 (0.37-1.38)
**MASD:** 0.786; **CIO:** 0.721				

Associations between lifestyle and metabolic factors, as well as family history of premature myocardial infarction and risk of early–onset myocardial infarction. Adj. level 1: adjusted for age, sex, country of birth, and years of school education; Adj. level 2: additionally adjusted for body mass index, smoking, and frequency of alcohol consumption; Adj. level 3: additionally adjusted for self–reported diabetes mellitus and hypertension. Percentile-based 95% bootstrap confidence intervals are reported for original data results. Median and percentile-based 95% confidence intervals of synthetic data results are printed in orange. adj., adjustment; 2.5% and 97.5%, regression 95% confidence interval limits; BMI, body mass index; MI, myocardial infarction; MASD, mean absolute standardised difference; CIO, confidence interval overlap

Figure S5. Replication of separate logistic regressions per variable, each adjusted for age, sex, country of birth, and years of school education - Table 2

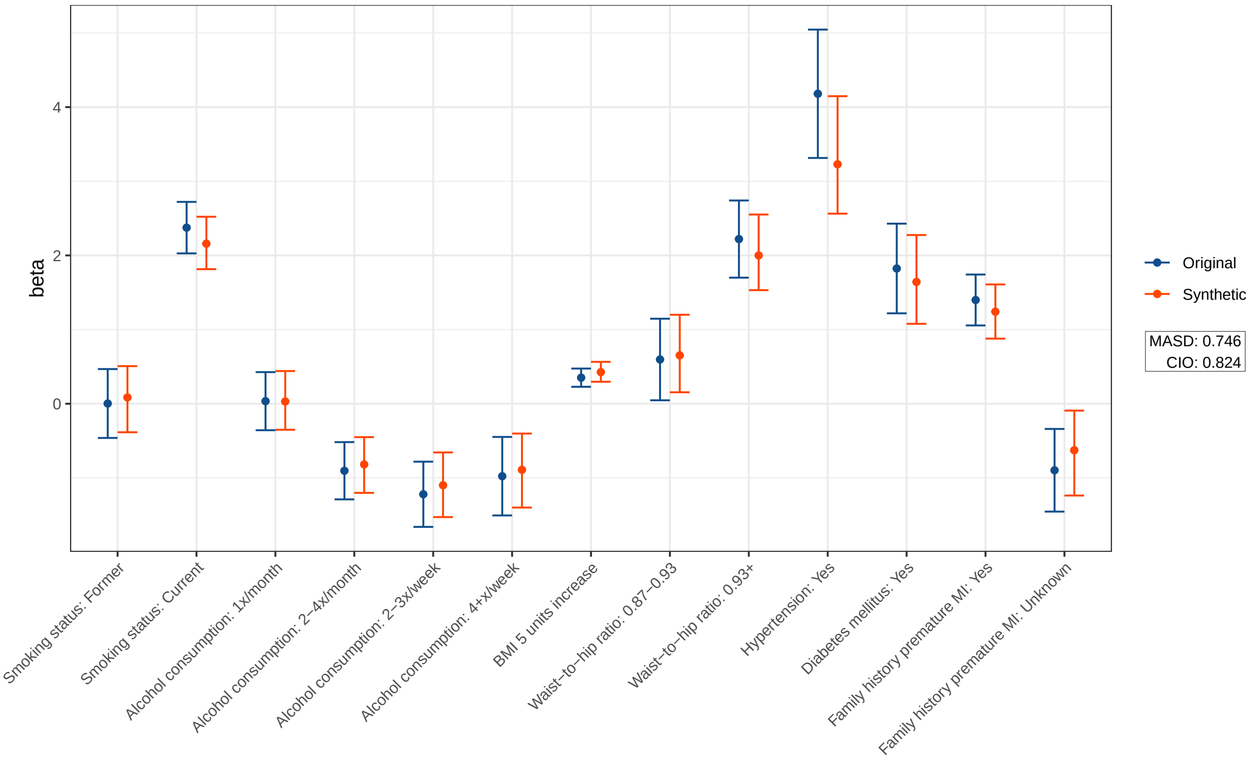

Associations between lifestyle and metabolic factors, as well as family history of premature MI and risk of early–onset MI. Median beta estimates and percentile-based 95% confidence intervals computed from the beta coefficient distribution across synthesis repetitions are reported for synthetic data. BMI, body mass index; MI, myocardial infarction; MASD, mean absolute standardised difference; CIO, confidence interval overlap

Table S10. Replication of Table 3

Subset	Adj. level	No. risk factors	Odds ratio	2.5%	97.5%
All	1	1	5.40 (3.08-12.42)	2.88 (1.80-5.20)	10.11 (5.28-29.45)
			4.40 (2.58-8.95)	2.48 (1.57-4.28)	7.81 (4.20-19.06)
		2+	43.81 (25.52-97.73)	23.27 (14.85-41.49)	82.48 (43.44-229.52)
			30.91 (17.28-64.17)	17.44 (10.68-30.29)	54.41 (28.09-139.38)
		1 unit increase	6.41 (5.25-8.16)	5.12 (4.26-6.40)	8.01 (6.47-10.39)
			5.32 (4.18-6.81)	4.33 (3.47-5.44)	6.53 (5.05-8.52)
	2	1	23.04 (9.57-74.53)	9.14 (3.95-24.88)	58.09 (22.42-227.19)
			11.50 (4.82-31.41)	4.90 (2.16-12.22)	26.92 (10.67-85.09)
		2+	582.31 (140.81-3606.48)	136.45 (34.21-664.72)	2485.08 (563.87-19064.33)
			141.81 (35.23-672.94)	37.54 (10.03-157.69)	533.45 (119.85-2921.45)
		1 unit increase	21.14 (12.35-44.31)	11.71 (7.18-22.41)	38.16 (21.17-89.43)
			9.92 (5.59-18.28)	6.09 (3.66-10.48)	16.12 (8.54-32.03)
Family history of premature MI: No	1	1	5.49 (3.02-14.18)	2.74 (1.66-5.23)	11.00 (5.39-38.91)
			4.53 (2.45-10.30)	2.37 (1.42-4.30)	8.58 (4.24-25.40)
		2+	43.08 (23.96-108.52)	21.25 (13.02-40.77)	87.35 (43.95-293.87)
			31.43 (16.57-72.03)	16.41 (9.50-30.48)	60.11 (28.62-176.70)
		1 unit increase	6.24 (4.96-8.30)	4.80 (3.89-6.21)	8.10 (6.32-11.15)
			5.38 (4.12-7.01)	4.24 (3.30-5.38)	6.84 (5.14-9.13)
	2	1	5.49 (3.02-14.18)	2.74 (1.66-5.23)	11.00 (5.39-38.91)
			4.53 (2.45-10.30)	2.37 (1.42-4.30)	8.58 (4.24-25.40)
		2+	43.08 (23.96-108.52)	21.25 (13.02-40.77)	87.35 (43.95-293.87)
			31.43 (16.57-72.03)	16.41 (9.50-30.48)	60.11 (28.62-176.70)
		1 unit increase	6.24 (4.96-8.30)	4.80 (3.89-6.21)	8.10 (6.32-11.15)
			5.38 (4.12-7.01)	4.24 (3.30-5.38)	6.84 (5.14-9.13)
Family history of premature MI: Yes	1	1	4.14 (1.23-44652545.64)	0.84 (0.00-1.75)	20.41 (4.65-Inf)
			4.03 (1.06-36378208.19)	0.72 (0.00-1.76)	19.36 (3.65-Inf)
		2+	42.88 (13.83-542617242.56)	8.59 (0.00-21.15)	214.19 (49.90-Inf)
			30.00 (7.48-302773299.34)	5.28 (0.00-13.71)	145.45 (25.38-Inf)
		1 unit increase	7.49 (4.63-16.75)	4.15 (2.72-7.64)	13.49 (7.85-37.15)
			5.29 (3.11-9.76)	3.14 (2.00-5.16)	8.94 (4.80-18.64)
	2	1	4.14 (1.23-44652545.64)	0.84 (0.00-1.75)	20.41 (4.65-Inf)
			4.03 (1.06-36378208.19)	0.72 (0.00-1.76)	19.36 (3.65-Inf)
		2+	42.88 (13.83-542617242.56)	8.59 (0.00-21.15)	214.19 (49.90-Inf)
			30.00 (7.48-302773299.34)	5.28 (0.00-13.71)	145.45 (25.38-Inf)
		1 unit increase	7.49 (4.63-16.75)	4.15 (2.72-7.64)	13.49 (7.85-37.15)
			5.29 (3.11-9.76)	3.14 (2.00-5.16)	8.94 (4.80-18.64)
Sex: Male	1	1	4.13 (2.22-11.14)	2.01 (1.20-3.80)	8.50 (4.05-32.30)
			3.89 (2.07-9.56)	1.97 (1.18-3.67)	7.65 (3.58-25.53)
		2+	34.68 (19.14-91.16)	16.92 (10.47-31.86)	71.08 (35.07-275.98)
			27.99 (14.97-67.03)	14.31 (8.59-26.36)	54.50 (25.97-173.90)
		1 unit increase	6.38 (5.07-8.39)	4.98 (4.03-6.39)	8.17 (6.40-10.98)
			5.39 (4.15-7.10)	4.28 (3.38-5.51)	6.79 (5.14-9.15)
	2	1	4.21 (2.25-11.05)	2.02 (1.20-3.82)	8.75 (4.19-32.93)
			3.78 (1.97-9.53)	1.91 (1.12-3.64)	7.49 (3.48-24.91)
		2+	34.38 (19.01-92.57)	16.53 (10.08-31.56)	71.50 (34.85-279.20)
			26.43 (13.88-64.72)	13.39 (7.83-24.79)	51.85 (24.40-168.07)
		1 unit increase	6.24 (4.94-8.40)	4.84 (3.90-6.31)	8.06 (6.27-11.17)
			5.22 (4.01-6.91)	4.13 (3.24-5.36)	6.61 (4.97-8.97)
Sex: Female	1	1	11.36 (3.96-148177227.37)	3.12 (0.00-7.01)	41.32 (11.11-Inf)
			6.32 (2.25-37.78)	2.04 (0.69-4.53)	19.03 (5.58-297.28)
		2+	78.34 (25.92-1049261545.60)	19.59 (0.00-52.43)	313.30 (79.69-Inf)
			40.33 (13.80-255.58)	12.13 (4.52-28.78)	130.47 (35.30-2095.27)
		1 unit increase	6.69 (4.50-13.35)	3.85 (2.74-6.43)	11.64 (7.28-27.97)
			5.26 (3.35-9.17)	3.29 (2.23-5.12)	8.46 (5.01-16.58)
	2	1	10.38 (3.60-152846697.04)	2.82 (0.00-6.78)	38.17 (10.25-Inf)
			6.14 (2.17-36.91)	1.95 (0.65-4.40)	18.71 (5.43-286.80)
		2+	71.82 (24.41-1198973535.71)	17.77 (0.00-50.01)	290.20 (77.07-Inf)
			39.15 (13.24-254.66)	11.52 (4.22-28.10)	128.01 (34.30-2133.37)
		1 unit increase	6.73 (4.48-14.32)	3.80 (2.66-6.59)	11.91 (7.49-31.43)
			5.19 (3.29-9.50)	3.19 (2.14-5.12)	8.43 (4.93-17.53)
**MASD:** 0.576; **CIO:** 0.671					

Joint associations of lifestyle and metabolic factors with risk of early–onset myocardial infarction, overall and by family history of premature myocardial infarction and sex. Percentile-based 95% bootstrap confidence intervals are reported for original data results. Median and percentile-based 95% confidence intervals of synthetic data results are printed in orange. adj., adjustment; No., number; 2.5% and 97.5%, regression 95% confidence interval limits; BMI, body mass index; MI, myocardial infarction; MASD, mean absolute standardised difference; CIO, confidence interval overlap

B.4 Breau et al.[7]

Table S11. Replication of Table 3

Variable	Everyone	Toddlers 1.5-2 yrs	Preschoolers 3-4 yrs	Preschoolers 3-6 yrs	School aged 5-6 yrs
Number of participants (n)	262 (262-262)	96 (80-111)	121 (105-136)	166 (151-182)	45 (34-57)
	262 (262-262)	84 (69-100)	143 (126-159.02)	178 (162-193)	35 (24-48)
Age (years) (mean)	3.62 (3.46-3.77)	2.26 (2.17-2.34)	3.96 (3.86-4.06)	4.40 (4.26-4.54)	5.58 (5.49-5.68)
	3.64 (3.50-3.78)	2.39 (2.30-2.47)	3.94 (3.84-4.03)	4.23 (4.12-4.36)	5.44 (5.34-5.54)
Age (years) (SD)	1.28 (1.20-1.35)	0.42 (0.38-0.45)	0.59 (0.54-0.64)	0.90 (0.83-0.96)	0.33 (0.27-0.37)
	1.10 (1.03-1.18)	0.38 (0.33-0.42)	0.56 (0.52-0.60)	0.79 (0.72-0.86)	0.30 (0.23-0.36)
Height (cm) (mean)	99.29 (97.99-100.61)	88.29 (87.24-89.31)	101.76 (100.75-102.79)	104.75 (103.64-105.92)	112.73 (111.30-114.14)
	99.37 (98.18-100.44)	92.74 (90.76-94.73)	100.80 (99.36-102.33)	102.22 (100.81-103.57)	107.72 (104.53-110.78)
Height (cm) (SD)	10.22 (9.49-10.92)	4.99 (4.30-5.57)	5.72 (4.99-6.46)	7.34 (6.58-8.06)	4.75 (3.78-5.50)
	9.11 (8.44-9.81)	7.70 (6.41-9.10)	7.64 (6.80-8.45)	8.11 (7.28-8.94)	7.40 (5.36-9.62)
Weight (kg) (mean)	15.92 (15.52-16.32)	13.02 (12.68-13.35)	16.59 (16.21-16.99)	17.58 (17.16-18.02)	20.21 (19.44-21.06)
	16.04 (15.66-16.42)	14.11 (13.57-14.68)	16.48 (16.01-16.98)	16.95 (16.49-17.41)	18.81 (17.71-19.90)
Weight (kg) (SD)	3.32 (3.00-3.64)	1.67 (1.40-1.93)	2.17 (1.87-2.48)	2.86 (2.49-3.23)	2.83 (2.12-3.41)
	3.00 (2.74-3.27)	2.29 (1.85-2.82)	2.63 (2.28-2.99)	2.86 (2.54-3.20)	2.94 (2.23-3.68)
BMI z score (mean)	0.56 (0.44-0.68)	0.86 (0.63-1.11)	0.44 (0.28-0.59)	0.40 (0.27-0.54)	0.31 (0.02-0.62)
	0.54 (0.29-0.79)	0.54 (0.09-1.00)	0.55 (0.22-0.90)	0.54 (0.24-0.85)	0.49 (-0.14-1.13)
BMI z score (SD)	0.99 (0.87-1.13)	1.08 (0.84-1.34)	0.85 (0.73-0.95)	0.91 (0.79-1.03)	1.07 (0.77-1.38)
	1.92 (1.64-2.19)	1.94 (1.53-2.42)	1.94 (1.61-2.31)	1.90 (1.60-2.22)	1.66 (1.17-2.31)
Valid days (mean)	7.65 (7.51-7.79)	7.53 (7.28-7.76)	7.66 (7.46-7.84)	7.72 (7.55-7.88)	7.87 (7.53-8.18)
	7.56 (7.42-7.68)	7.40 (7.15-7.64)	7.61 (7.43-7.77)	7.63 (7.47-7.78)	7.72 (7.35-8.03)
Valid days (SD)	1.14 (0.98-1.30)	1.28 (0.99-1.53)	1.03 (0.79-1.26)	1.06 (0.86-1.24)	1.12 (0.74-1.44)
	1.06 (0.95-1.18)	1.15 (0.93-1.37)	1.01 (0.86-1.17)	1.01 (0.88-1.16)	0.97 (0.68-1.31)
Valid min/day (mean)	714.24 (705.47-722.91)	681.41 (666.11-696.45)	727.65 (716.75-737.52)	733.23 (723.60-742.74)	748.24 (724.64-769.14)
	713.16 (704.72-721.34)	688.42 (671.78-703.21)	721.33 (710.82-731.14)	725.11 (715.75-734.04)	740.30 (717.66-761.75)
Valid min/day (SD)	72.16 (64.43-79.98)	75.64 (62.81-87.18)	57.16 (47.29-67.62)	62.86 (54.52-71.89)	74.78 (56.23-89.77)
	66.32 (59.03-73.58)	70.28 (56.97-83.04)	59.46 (51.02-69.04)	60.75 (53.18-69.14)	61.12 (46.42-80.15)
Sex: Male (n)	126 (110-142)	40 (29-52)	60 (47-73)	86 (71-101)	26 (17-36)
	126 (110-142)	35 (24-46)	71 (58-86)	91 (76-106)	20 (12-30)
Sex: Female (n)	136 (120-152)	56 (43-70)	61 (47-74)	80 (65-94)	19 (11-27)
	136 (120-152)	49 (37-62)	71 (57-86)	86 (72-102)	15 (8-23)
Ethnicity: White (n)	192 (178-205)	78 (63-93)	85 (71-100)	114 (98-129)	29 (20-39)
	192 (178-206)	64 (50-78)	103 (87-118)	128 (111-143)	24 (16-35)
Ethnicity: Other (n)	70 (57-84)	18 (10-26)	36 (26-47)	52 (40-65)	16 (9-24)
	70 (56-84)	20 (12-28)	40 (28-51)	50 (38-63)	11 (5-18)
HHI: No answer (n)	10 (4-17)	2 (0-5)	6 (2-11)	8 (3-14)	2 (0-5)
	10 (4-16)	3 (0-7)	6 (2-11)	7 (2-12)	1 (0-4)
HHI: <$100,000 (n)	122 (106-138)	45 (33-57)	53 (41-66)	77 (63-91)	24 (15-34)
	122 (106-137)	38 (28-50)	67 (52-81)	84 (69-99)	16 (9-25)
HHI: $100,000+ (n)	130 (114-146)	49 (37-62)	62 (49-75)	81 (66-96)	19 (11-27)
	130 (115-146)	43 (31-55)	70 (56-84)	87 (72-102)	17 (10-26)
**MASD:** 2.132; **CIO:** 0.627					

Characteristics of study population for the entire sample population and by age group. Percentile-based 95% bootstrap confidence intervals are reported for original data results. Median and percentile-based 95% confidence intervals of synthetic data results are printed in orange. n, number of participants; SD, standard deviation; BMI, body mass index; HHI, household income; MASD, mean absolute standardised difference; CIO, confidence interval overlap

Table S12. Replication of Figure 1

Set of cutpoints	SED	LPA	MVPA
van Cauwenberghe	615.34 (607.77-623.08)	31.81 (30.92-32.68)	67.09 (64.70-69.44)
	614.10 (606.94-620.96)	31.88 (30.99-32.73)	67.22 (64.97-69.54)
Pate	574.95 (567.58-582.27)	48.54 (47.33-49.75)	90.75 (87.77-93.69)
	573.58 (566.89-579.93)	48.67 (47.47-49.84)	90.92 (88.07-93.83)
Puyau	574.95 (567.58-582.27)	93.60 (91.17-96.06)	45.69 (43.94-47.51)
	573.61 (566.97-580.11)	93.73 (91.37-96.15)	45.82 (44.07-47.61)
Trost	515.14 (508.14-522.02)	108.35 (105.93-110.76)	90.75 (87.77-93.69)
	513.81 (507.48-520.02)	108.42 (106.15-110.82)	90.91 (87.97-93.86)
Butte (VA)	515.14 (508.14-522.02)	126.38 (123.55-129.24)	72.73 (70.22-75.23)
	513.77 (507.34-520.01)	126.45 (123.65-129.21)	72.89 (70.40-75.34)
Pate & Pfeiffer	509.42 (502.33-516.36)	114.14 (111.68-116.66)	90.69 (87.68-93.61)
	508.20 (501.82-514.43)	114.14 (111.77-116.55)	90.83 (87.98-93.71)
Evenson	501.33 (494.38-508.17)	145.78 (142.58-149.04)	67.13 (64.80-69.51)
	499.98 (493.70-506.25)	145.88 (142.72-148.93)	67.29 (65.00-69.66)
Costa (VA)	501.50 (494.46-508.43)	133.90 (131.02-136.82)	78.84 (76.14-81.47)
	500.24 (493.91-506.69)	133.89 (131.08-136.73)	79.01 (76.40-81.63)
Costa (VM)	463.98 (457.37-470.51)	163.44 (159.97-166.80)	86.82 (83.68-89.73)
	463.08 (456.92-468.87)	163.34 (160.04-166.62)	86.81 (83.95-89.85)
Butte (VM)	437.20 (430.77-443.48)	176.36 (172.73-179.96)	100.68 (97.21-103.91)
	436.05 (430.09-442.23)	176.33 (172.97-179.87)	100.65 (97.42-103.98)
**MASD:** 0.172; **CIO:** 0.950			

Calculated average valid wear time minutes per day spent in SED, LPA, and MVPA according to the 10 different sets of ActiGraph cutpoints examined. Cutpoints were applied to the entire cohort. Percentile-based 95% bootstrap confidence intervals are reported for original data results. Median and percentile-based 95% confidence intervals of synthetic data results are printed in orange. SED, sedentary behaviour; LPA, light physical activity; MVPA, moderate to vigorous physical activity; VA, vertical axis; VM, vector magnitude; MASD, mean absolute standardised difference; CIO, confidence interval overlap

Figure S6. Replication of Figure 1

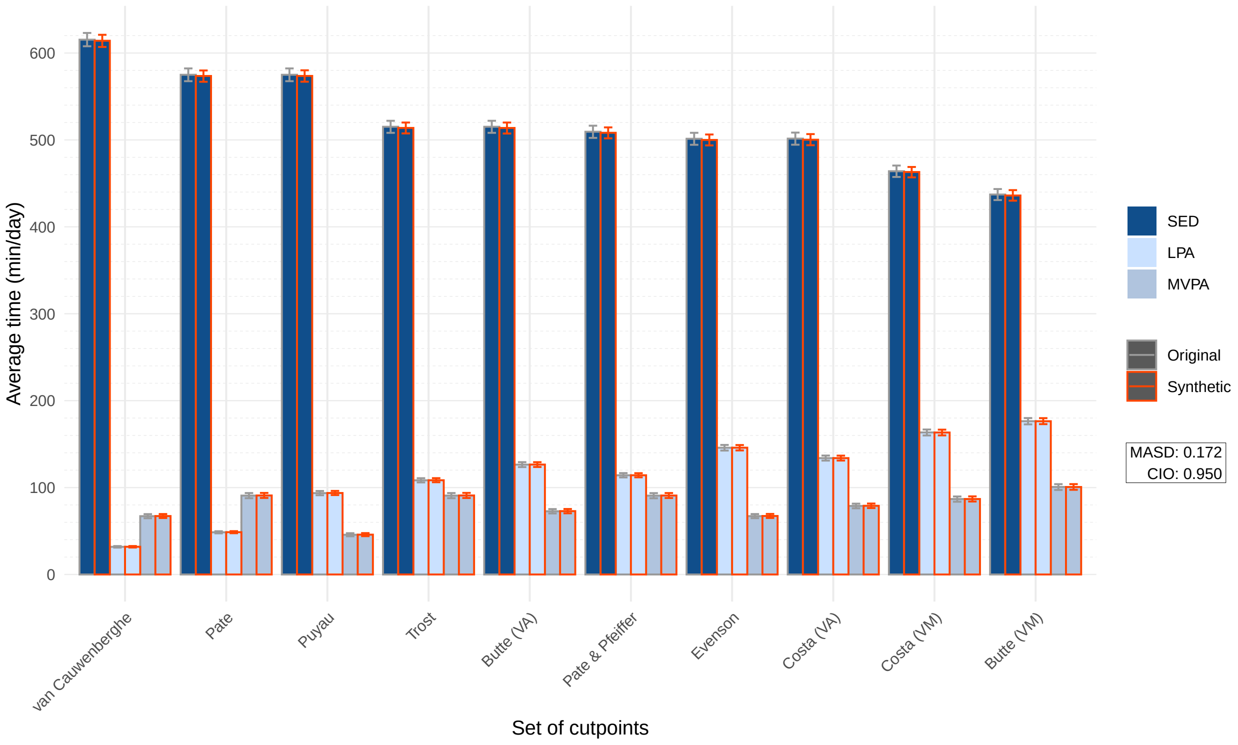

Calculated average valid wear time minutes per day spent in SED, LPA, and MVPA according to the 10 different sets of ActiGraph cutpoints examined. Cutpoints were applied to the entire cohort. Percentile-based 95% bootstrap confidence intervals are reported for original data results. Median and percentile-based 95% confidence intervals of synthetic data results are reported. SED, sedentary behaviour; LPA, light physical activity; MVPA, moderate to vigorous physical activity; VA, vertical axis; VM, vector magnitude; MASD, mean absolute standardised difference; CIO, confidence interval overlap

Table S13. Replication of Figure 2

Age group	Set of cutpoints	SED	LPA	MVPA
Toddlers (1.5-2 years)	Trost	491.66 (479.62-503.62)	107.73 (103.48-111.87)	82.01 (77.21-86.77)
		501.96 (488.56-513.23)	105.80 (101.54-110.26)	80.77 (76.03-85.99)
	Costa (VA)	477.97 (465.90-489.90)	132.69 (127.62-137.62)	70.75 (66.49-75.13)
		488.53 (475.31-499.88)	130.24 (125.12-135.73)	69.67 (65.49-74.12)
	Costa (VM)	449.33 (438.15-461.16)	157.79 (151.94-163.57)	74.28 (69.63-78.73)
		456.76 (443.80-468.22)	157.03 (151.06-163.17)	74.46 (69.94-79.37)
Preschoolers (3-6 years)	van Cauwenberghe	628.95 (620.51-637.51)	32.99 (32.00-34.02)	71.29 (68.60-74.05)
		620.87 (612.79-628.62)	33.01 (32.00-34.04)	71.13 (68.36-73.84)
	Pate	588.03 (579.86-596.40)	49.40 (47.98-50.86)	95.81 (92.42-99.29)
		579.69 (572.13-587.05)	49.68 (48.26-51.14)	95.71 (92.43-99.02)
	Butte (VA)	528.71 (520.88-536.86)	127.41 (124.16-130.84)	77.11 (74.29-80.05)
		519.72 (511.95-526.81)	128.38 (125.16-131.66)	77.00 (74.20-79.89)
	Pate & Pfeiffer	522.89 (515.09-531.00)	114.54 (111.73-117.57)	95.81 (92.42-99.29)
		513.94 (506.50-521.20)	115.45 (112.75-118.21)	95.61 (92.34-98.93)
	Butte (VM)	445.84 (438.62-453.26)	178.91 (174.60-183.16)	108.48 (104.78-112.35)
		439.11 (431.86-445.84)	178.87 (174.77-182.94)	107.00 (103.16-110.65)
School aged (5-6 years)	Puyau	598.02 (579.82-614.92)	97.55 (91.59-103.70)	52.66 (48.23-57.20)
		588.02 (570.17-604.99)	99.42 (92.55-106.31)	52.78 (47.91-57.90)
	Evenson	526.41 (509.72-541.64)	145.98 (138.04-154.08)	75.84 (69.98-81.77)
		514.18 (497.56-530.53)	149.58 (140.40-158.77)	76.33 (69.70-82.95)
**MASD:** 0.834; **CIO:** 0.788				

Calculated average valid wear time minutes per day spent in SED, LPA, and MVPA according to age-appropriate ActiGraph cutpoint sets by age group. Percentile-based 95% bootstrap confidence intervals are reported for original data results. Median and percentile-based 95% confidence intervals of synthetic data results are printed in orange. SED, sedentary behaviour; LPA, light physical activity; MVPA, moderate to vigorous physical activity; VA, vertical axis; VM, vector magnitude; MASD, mean absolute standardised difference; CIO, confidence interval overlap

Figure S7. Replication of Figure 2

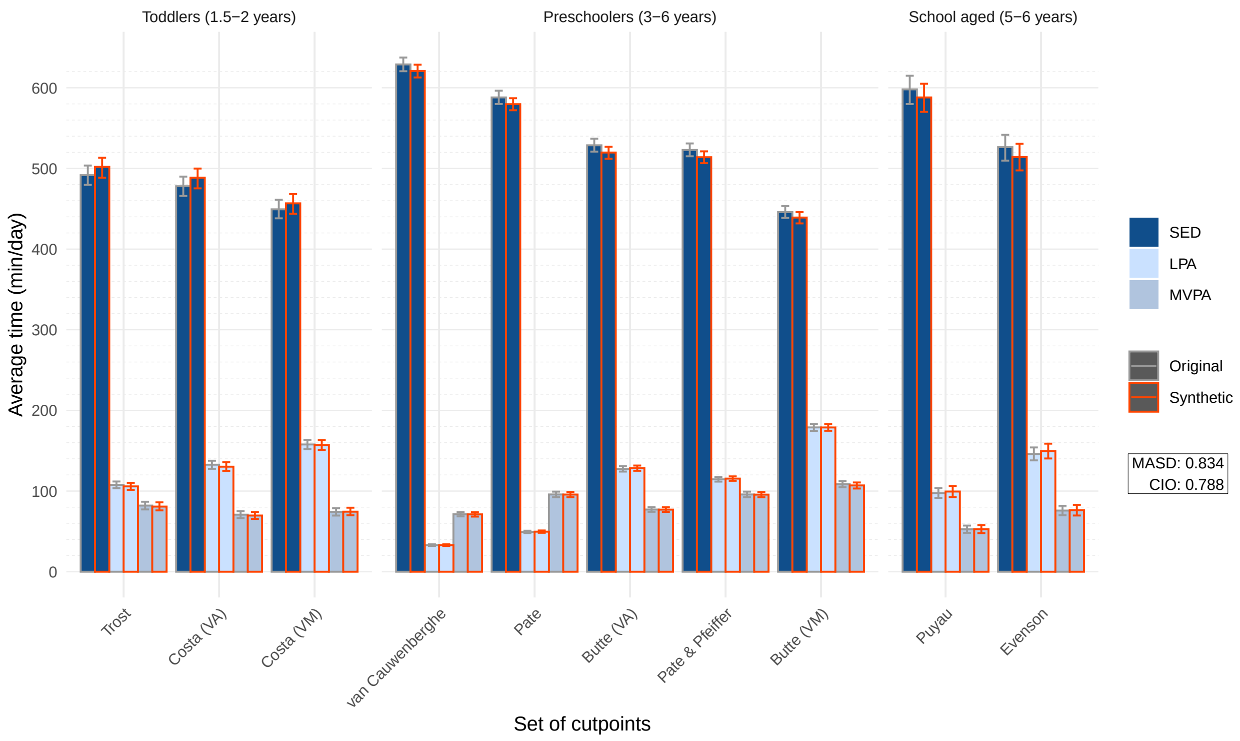

Calculated average valid wear time minutes per day spent in SED, LPA, and MVPA according to age-appropriate ActiGraph cutpoint sets by age group. Percentile-based 95% bootstrap confidence intervals are reported for original data results. Median and percentile-based 95% confidence intervals of synthetic data results are reported. SED, sedentary behaviour; LPA, light physical activity; MVPA, moderate to vigorous physical activity; VA, vertical axis; VM, vector magnitude; MASD, mean absolute standardised difference; CIO, confidence interval overlap

Table S14. Replication of Figure 3

Age group	Set of cutpoints	Proportion meeting PA guideline
Toddlers (1.5-2 years)	Trost	0.62 (0.53-0.72)
		0.59 (0.48-0.71)
	Costa (VA)	0.72 (0.62-0.80)
		0.70 (0.60-0.80)
	Costa (VM)	0.90 (0.83-0.95)
		0.88 (0.80-0.95)
Preschoolers (3-6 years)	van Cauwenberghe	0.22 (0.16-0.28)
		0.15 (0.10-0.21)
	Pate	0.37 (0.30-0.45)
		0.30 (0.24-0.38)
	Butte (VA)	0.75 (0.69-0.82)
		0.73 (0.67-0.80)
	Pate & Pfeiffer	0.84 (0.78-0.89)
		0.82 (0.76-0.87)
	Butte (VM)	0.98 (0.96-1.00)
		0.98 (0.96-1.00)
School aged (5-6 years)	Puyau	0.31 (0.17-0.45)
		0.32 (0.17-0.49)
	Evenson	0.80 (0.68-0.91)
		0.79 (0.64-0.92)
**MASD:** 0.700; **CIO:** 0.814		

Percentage of sample meeting age-appropriate guidelines for physical activity according to age-appropriate ActiGraph cutpoints by age group. Percentile-based 95% bootstrap confidence intervals are reported for original data results. Median and percentile-based 95% confidence intervals of synthetic data results are printed in orange. PA, physical activity; VA, vertical axis; VM, vector magnitude; MASD, mean absolute standardised difference; CIO, confidence interval overlap

Figure S8. Replication of Figure 3

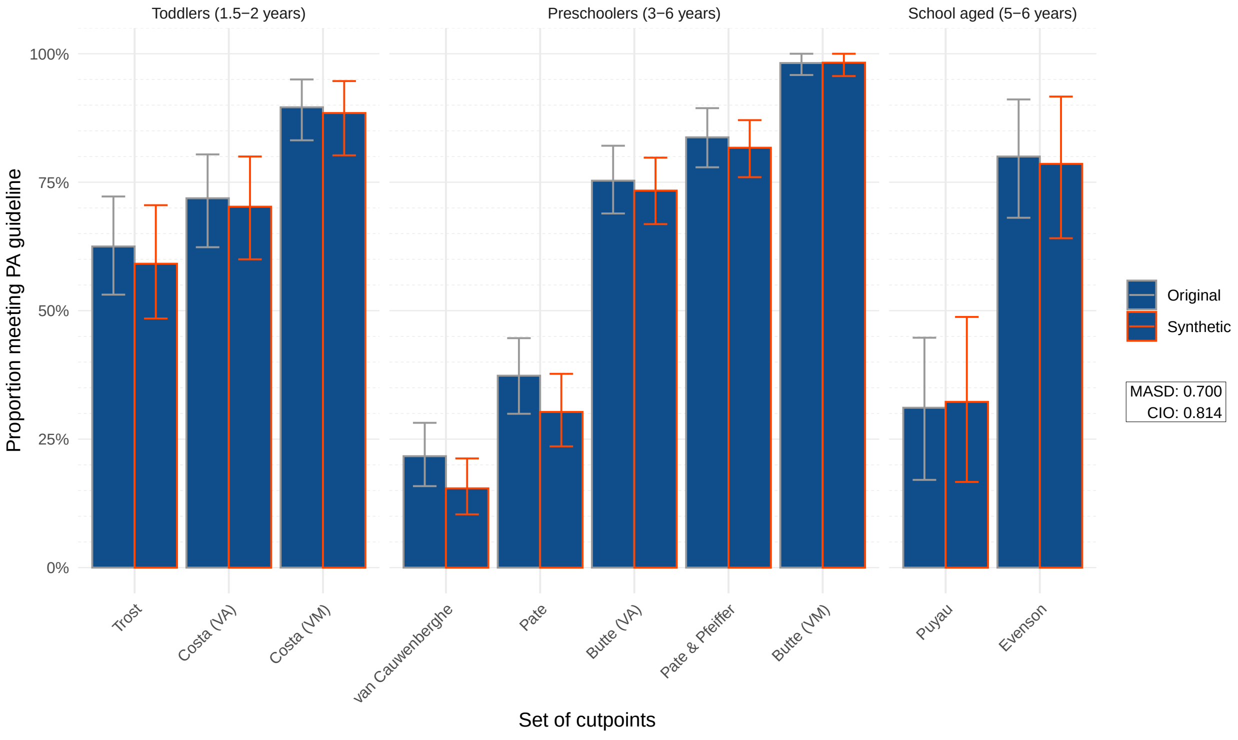

Percentage of sample meeting age-appropriate guidelines for physical activity according to age-appropriate ActiGraph cutpoints by age group. Percentile-based 95% bootstrap confidence intervals are reported for original data results. Median and percentile-based 95% confidence intervals of synthetic data results are reported. PA, physical activity; VA, vertical axis; VM, vector magnitude; MASD, mean absolute standardised difference; CIO, confidence interval overlap

B.5 Berger et al.[8]

Table S15. Replication of Table 1 (general and task-specific synthesis)

Variable	Value
Number of participants (n)	113527 (113527-113527)
	113527 (113527-113527)
	113527 (113527-113527)
Sex: Female (%)	51.75 (51.47-52.03)
	51.76 (51.45-52.05)
	51.76 (51.46-52.05)
Age (mean years)	49.99 (49.92-50.07)
	49.69 (49.53-49.84)
	49.84 (49.74-49.94)
Age: 20-29 years (%)	9.08 (8.91-9.25)
	5.37 (4.98-6.08)
	7.98 (7.56-8.32)
Age: 60+ years (%)	26.76 (26.49-27.01)
	22.02 (21.36-22.83)
	25.50 (24.86-26.17)
Follow up time (mean years)	2.66 (2.65-2.67)
	2.68 (2.66-2.70)
	2.68 (2.67-2.69)
Education: Hauptschule (%)	11.23 (11.04-11.42)
	11.23 (11.05-11.42)
	11.23 (11.04-11.41)
Education: Realschule (%)	28.62 (28.36-28.89)
	28.61 (28.34-28.89)
	28.62 (28.36-28.88)
Education: (Fach-)Abitur (%)	58.69 (58.41-58.98)
	58.69 (58.39-58.98)
	58.69 (58.41-58.97)
Education: none/other (%)	1.46 (1.39-1.53)
	1.46 (1.39-1.53)
	1.46 (1.38-1.52)
Living in relationship: Yes (%)	76.80 (76.55-77.04)
	76.80 (76.54-77.04)
	76.80 (76.55-77.04)
PHQ-9 score baseline examination woman (mean)	4.14 (4.11-4.17)
	4.07 (4.02-4.12)
	4.13 (4.10-4.16)
PHQ-9 score baseline examination woman (SD)	3.64 (3.60-3.67)
	3.28 (3.19-3.37)
	3.64 (3.60-3.68)
GAD-7 score baseline examination woman (mean)	3.44 (3.41-3.47)
	3.37 (3.33-3.42)
	3.43 (3.40-3.46)
GAD-7 score baseline examination woman (SD)	3.23 (3.20-3.26)
	2.89 (2.81-2.98)
	3.23 (3.19-3.26)
PHQ-9 score baseline examination man (mean)	3.24 (3.21-3.27)
	3.27 (3.23-3.32)
	3.24 (3.21-3.27)
PHQ-9 score baseline examination man (SD)	3.32 (3.27-3.36)
	3.02 (2.94-3.10)
	3.32 (3.28-3.36)
GAD-7 score baseline examination man (mean)	2.58 (2.56-2.61)
	2.62 (2.59-2.66)
	2.59 (2.57-2.62)
GAD-7 score baseline examination man (SD)	2.81 (2.78-2.85)
	2.59 (2.53-2.65)
	2.82 (2.79-2.86)
**General:** **MASD:** 9.183; **CIO:** 0.438	
**Task-specific:** **MASD:** 1.780; **CIO:** 0.747	

Characteristics of German National Cohort (NAKO Gesundheitsstude) participants taking part in the COVID survey in May 2020. Percentile-based 95% bootstrap confidence intervals are reported for original data results. Median and percentile-based 95% confidence intervals of synthetic data results are reported, general synthesis replication results are printed in orange, task-specific synthesis replication results in violet. PHQ-9, nine-item Patient Health Questionnaire; GAD-7, Generalised Anxiety Disorder seven-item scale; SD, standard deviation; MASD, mean absolute standardised difference; CIO, confidence interval overlap

Table S16. Replication of Table 2

Variable	Value	Value woman	Value men
Feeling of missing company of others (%)	79.88 (79.64-80.11)	83.49 (83.17-83.79)	76.00 (75.63-76.36)
	79.87 (79.63-80.10)	82.31 (81.91-82.72)	77.24 (76.79-77.69)
Feeling of being left out (%)	29.88 (29.62-30.16)	35.09 (34.70-35.47)	24.29 (23.92-24.67)
	29.88 (29.61-30.14)	34.11 (33.67-34.55)	25.34 (24.89-25.77)
Feeling of being socially isolated (%)	34.87 (34.61-35.15)	40.48 (40.07-40.89)	28.85 (28.47-29.22)
	34.87 (34.58-35.17)	39.28 (38.79-39.79)	30.14 (29.62-30.65)
Sum score loneliness (mean)	4.93 (4.92-4.94)	5.17 (5.15-5.18)	4.68 (4.67-4.69)
	4.93 (4.92-4.94)	5.11 (5.09-5.13)	4.74 (4.72-4.76)
Sum score loneliness (SD)	1.61 (1.60-1.61)	1.67 (1.67-1.68)	1.49 (1.48-1.50)
	1.44 (1.41-1.48)	1.49 (1.45-1.53)	1.37 (1.34-1.41)
Proportion of lonely participants (%)	31.67 (31.40-31.95)	37.43 (37.04-37.83)	25.49 (25.14-25.85)
	30.77 (30.15-31.32)	35.60 (34.81-36.35)	25.56 (24.93-26.16)
Subjective increase of loneliness during pandemic (%)	47.03 (46.74-47.34)	51.00 (50.61-51.42)	42.78 (42.36-43.21)
	47.04 (46.73-47.33)	50.48 (50.03-50.92)	43.35 (42.89-43.81)
Fear of natural disasters (%)	11.88 (11.69-12.07)	15.76 (15.46-16.08)	7.71 (7.49-7.93)
	11.88 (11.69-12.07)	14.39 (14.02-14.82)	9.19 (8.79-9.55)
Fear of cancer (%)	53.10 (52.81-53.39)	58.91 (58.51-59.32)	46.86 (46.41-47.27)
	53.10 (52.81-53.38)	57.29 (56.79-57.85)	48.60 (48.00-49.10)
Fear of cardiac infarction (%)	36.57 (36.28-36.85)	36.91 (36.51-37.32)	36.21 (35.80-36.62)
	36.57 (36.29-36.84)	37.69 (37.26-38.11)	35.36 (34.93-35.79)
Fear of COVID infection (%)	34.52 (34.24-34.80)	38.56 (38.15-38.97)	30.17 (29.79-30.56)
	34.51 (34.25-34.79)	37.63 (37.20-38.09)	31.17 (30.73-31.64)
PHQ-9 score COVID survey (mean)	4.06 (4.04-4.09)	4.58 (4.55-4.62)	3.50 (3.47-3.54)
	4.04 (4.01-4.07)	4.52 (4.48-4.57)	3.53 (3.49-3.56)
PHQ-9 score COVID survey (SD)	3.93 (3.90-3.95)	4.07 (4.03-4.11)	3.69 (3.65-3.73)
	3.59 (3.51-3.68)	3.71 (3.62-3.81)	3.38 (3.31-3.46)
GAD-7 score COVID survey (mean)	3.37 (3.35-3.39)	3.91 (3.88-3.94)	2.78 (2.76-2.81)
	3.35 (3.32-3.38)	3.83 (3.79-3.87)	2.84 (2.81-2.87)
GAD-7 score COVID survey (SD)	3.44 (3.41-3.46)	3.62 (3.59-3.65)	3.12 (3.09-3.16)
	3.14 (3.07-3.22)	3.27 (3.19-3.37)	2.91 (2.85-2.97)
**MASD:** 8.491; **CIO:** 0.281			

Relative frequency of loneliness and anxiety as well as depression and anxiety scores during pandemic measures in May 2020. Percentile-based 95% bootstrap confidence intervals are reported for original data results. Median and percentile-based 95% confidence intervals of synthetic data results are printed in orange. PHQ-9, nine-item Patient Health Questionnaire; GAD-7, Generalised Anxiety Disorder seven-item scale; SD, standard deviation; MASD, mean absolute standardised difference; CIO, confidence interval overlap

Table S17. Replication of multivariable linear regression - Table 3 (general and task-specific synthesis)

Variable	beta coefficient	2.5%	97.5%
(Intercept)	4.34 (4.27-4.41)	4.27 (4.20-4.34)	4.41 (4.34-4.48)
	4.30 (4.23-4.37)	4.24 (4.16-4.31)	4.37 (4.29-4.44)
	4.40 (4.32-4.49)	4.33 (4.25-4.41)	4.47 (4.39-4.56)
Age	0.00 (-0.00-0.00)	-0.00 (-0.00-0.00)	0.00 (0.00-0.00)
	-0.00 (-0.00-0.00)	-0.00 (-0.00–0.00)	0.00 (-0.00-0.00)
	0.00 (-0.00-0.00)	-0.00 (-0.00-0.00)	0.00 (-0.00-0.00)
Sex: Female	0.28 (0.26-0.30)	0.27 (0.25-0.28)	0.30 (0.28-0.32)
	0.18 (0.16-0.21)	0.17 (0.15-0.20)	0.20 (0.18-0.23)
	0.27 (0.25-0.29)	0.25 (0.23-0.27)	0.28 (0.26-0.30)
Education level	-0.04 (-0.05–0.03)	-0.05 (-0.07–0.04)	-0.03 (-0.04–0.02)
	-0.02 (-0.04–0.01)	-0.03 (-0.05–0.02)	-0.01 (-0.02-0.00)
	-0.04 (-0.06–0.03)	-0.06 (-0.07–0.04)	-0.03 (-0.04–0.02)
In relationship: Yes	-0.13 (-0.15–0.11)	-0.15 (-0.18–0.13)	-0.11 (-0.13–0.09)
	-0.09 (-0.11–0.06)	-0.11 (-0.13–0.08)	-0.07 (-0.09–0.04)
	-0.13 (-0.16–0.11)	-0.15 (-0.18–0.13)	-0.11 (-0.14–0.09)
PHP-9 score COVID survey	0.11 (0.10-0.11)	0.10 (0.10-0.11)	0.11 (0.11-0.11)
	0.10 (0.09-0.10)	0.09 (0.09-0.10)	0.10 (0.10-0.11)
	0.09 (0.08-0.10)	0.09 (0.08-0.09)	0.09 (0.09-0.10)
GAD-7 score COVID survey	0.06 (0.06-0.07)	0.06 (0.06-0.07)	0.07 (0.06-0.07)
	0.08 (0.07-0.09)	0.08 (0.07-0.08)	0.08 (0.08-0.09)
	0.08 (0.07-0.08)	0.07 (0.07-0.08)	0.08 (0.08-0.09)
Fear of COVID infection: Yes	0.10 (0.08-0.12)	0.08 (0.06-0.10)	0.12 (0.10-0.14)
	0.09 (0.07-0.11)	0.07 (0.05-0.09)	0.11 (0.09-0.13)
	0.10 (0.08-0.12)	0.08 (0.06-0.10)	0.12 (0.10-0.14)
Study center	-0.00 (-0.00–0.00)	-0.00 (-0.01–0.00)	-0.00 (-0.00-0.00)
	-0.00 (-0.00-0.00)	-0.00 (-0.00–0.00)	-0.00 (-0.00-0.00)
	-0.00 (-0.00–0.00)	-0.00 (-0.01–0.00)	-0.00 (-0.00-0.00)
**General:** **MASD:** 9.623; **CIO:** 0.276			
**Task-specific:** **MASD:** 4.708; **CIO:** 0.518			

Relationship between perceived loneliness and sociodemographic factors as well as symptoms of depression and anxiety among German National Cohort (NAKO Gesundheitsstudie) participants in May 2020. Percentile-based 95% bootstrap confidence intervals are reported for original data results. Median and percentile-based 95% confidence intervals of synthetic data results are reported, general synthesis replication results are printed in orange, task-specific synthesis replication results in violet. 2.5% and 97.5%, regression 95% confidence interval limits; PHQ-9, nine-item Patient Health Questionnaire; GAD-7, Generalised Anxiety Disorder seven-item scale; MASD, mean absolute standardised difference; CIO, confidence interval overlap

Figure S9. Replication of multivariable linear regression - Table 3 (general and task-specific synthesis)

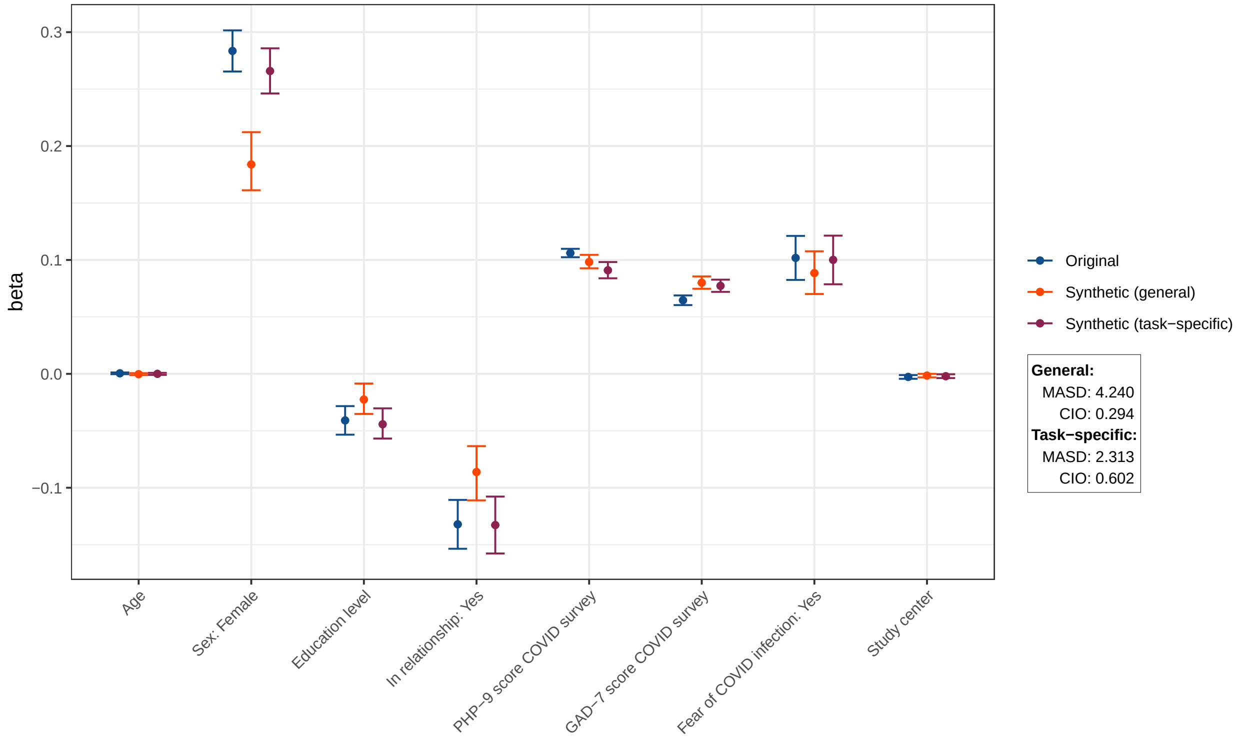

Relationship between perceived loneliness and sociodemographic factors as well as symptoms of depression and anxiety among German National Cohort (NAKO Gesundheitsstudie) participants in May 2020. Median beta estimates and percentile-based 95% confidence intervals computed from the beta coefficient distribution across synthesis repetitions are reported for synthetic data. PHQ-9, nine-item Patient Health Questionnaire; GAD-7, Generalised Anxiety Disorder seven-item scale; MASD, mean absolute standardised difference; CIO, confidence interval overlap

Table S18. Replication of Figure 1

Sex	Age group	Lonely participants (proportion)	Participants with increased loneliness (proportion)
Male	20-29	0.33 (0.32-0.35)	0.58 (0.57-0.60)
		0.33 (0.31-0.35)	0.57 (0.55-0.59)
	30-39	0.29 (0.28-0.30)	0.50 (0.48-0.51)
		0.30 (0.29-0.31)	0.51 (0.50-0.53)
	40-59	0.26 (0.25-0.26)	0.45 (0.44-0.46)
		0.26 (0.25-0.27)	0.46 (0.45-0.47)
	50-59	0.24 (0.23-0.25)	0.41 (0.40-0.42)
		0.24 (0.24-0.25)	0.41 (0.41-0.42)
	60-69	0.23 (0.23-0.24)	0.36 (0.35-0.36)
		0.23 (0.22-0.23)	0.36 (0.35-0.37)
	70+	0.23 (0.21-0.26)	0.32 (0.29-0.34)
		0.20 (0.16-0.24)	0.30 (0.25-0.34)
Female	20-29	0.47 (0.45-0.48)	0.65 (0.64-0.66)
		0.43 (0.41-0.45)	0.63 (0.61-0.65)
	30-39	0.44 (0.43-0.45)	0.59 (0.58-0.61)
		0.40 (0.39-0.42)	0.58 (0.57-0.59)
	40-59	0.35 (0.34-0.36)	0.51 (0.50-0.51)
		0.36 (0.35-0.37)	0.52 (0.51-0.53)
	50-59	0.35 (0.34-0.35)	0.48 (0.47-0.49)
		0.34 (0.33-0.35)	0.48 (0.47-0.49)
	60-69	0.37 (0.36-0.38)	0.46 (0.45-0.47)
		0.33 (0.31-0.34)	0.44 (0.43-0.45)
	70+	0.35 (0.32-0.38)	0.40 (0.37-0.44)
		0.29 (0.24-0.35)	0.37 (0.31-0.42)
**MASD:** 2.607; **CIO:** 0.499			

Proportion of lonely participants and of those, who felt more lonely during the pandemic than before, by sex and age group (in years). Percentile-based 95% bootstrap confidence intervals are reported for original data results. Median and percentile-based 95% confidence intervals of synthetic data results are printed in orange; MASD, mean absolute standardised difference; CIO, confidence interval overlap

Figure S10. Replication of Figure 1

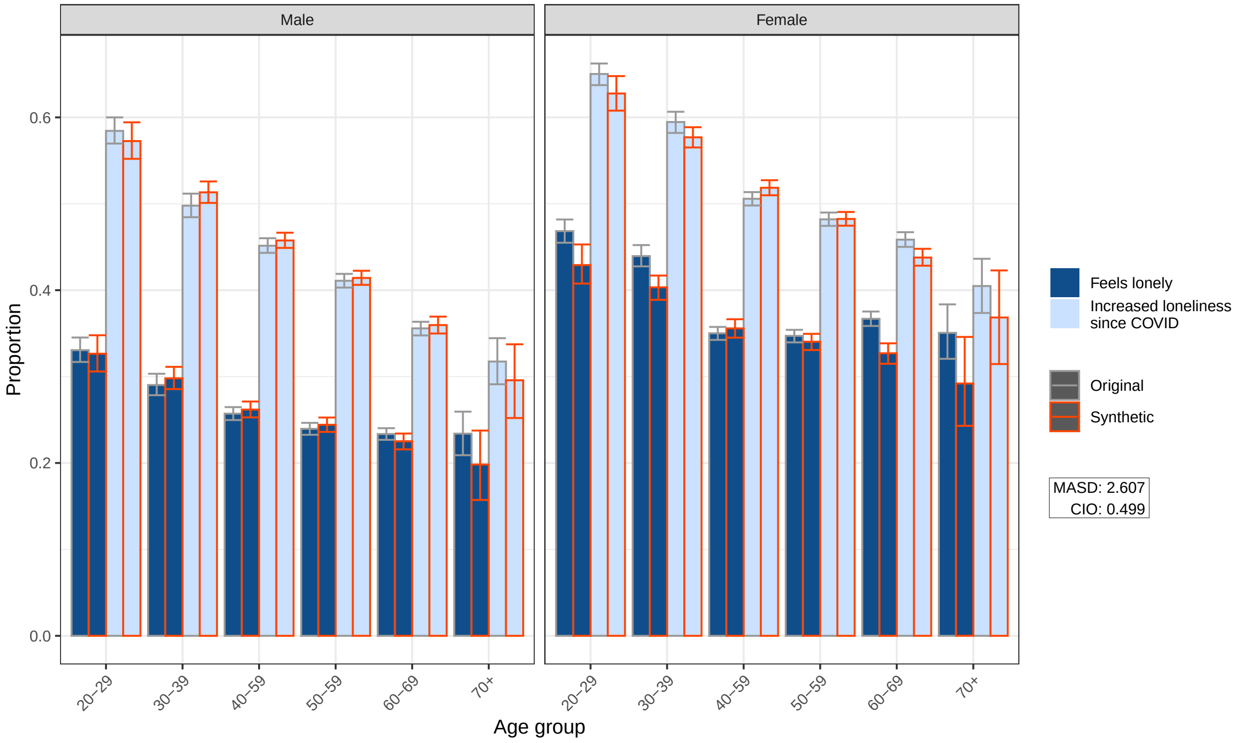

Proportion of lonely participants and of those, who felt more lonely during the pandemic than before, by sex and age group (years). Percentile-based 95% bootstrap confidence intervals are reported for original data results. Median and percentile-based 95% confidence intervals of synthetic data results are reported; MASD, mean absolute standardised difference; CIO, confidence interval overlap

Table S19. Replication of Figure 2

Sex	Loneliness score	PHQ-9 score COVID survey (mean)	GAD-7 score COVID survey (mean)
Male	3	2.56 (2.51-2.61)	2.01 (1.96-2.05)
		2.38 (2.28-2.46)	1.87 (1.79-1.94)
	4	2.63 (2.59-2.67)	2.09 (2.06-2.13)
		2.65 (2.59-2.70)	2.11 (2.06-2.15)
	5	3.46 (3.39-3.53)	2.77 (2.71-2.83)
		3.62 (3.54-3.70)	2.93 (2.87-3.00)
	6	4.77 (4.68-4.88)	3.82 (3.74-3.90)
		4.69 (4.60-4.81)	3.83 (3.73-3.92)
	7	5.44 (5.31-5.58)	4.31 (4.21-4.43)
		5.69 (5.52-5.89)	4.63 (4.49-4.78)
	8	6.39 (6.17-6.64)	5.09 (4.90-5.28)
		6.82 (6.52-7.20)	5.54 (5.29-5.85)
	9	9.03 (8.66-9.42)	7.15 (6.84-7.47)
		8.49 (7.93-9.11)	6.87 (6.43-7.36)
Female	3	3.34 (3.26-3.41)	2.89 (2.82-2.96)
		3.03 (2.93-3.13)	2.55 (2.45-2.64)
	4	3.23 (3.18-3.27)	2.80 (2.76-2.84)
		3.27 (3.21-3.33)	2.76 (2.71-2.81)
	5	4.22 (4.15-4.29)	3.62 (3.55-3.68)
		4.32 (4.24-4.40)	3.67 (3.60-3.73)
	6	5.24 (5.15-5.32)	4.47 (4.39-4.54)
		5.35 (5.25-5.45)	4.54 (4.45-4.63)
	7	6.12 (6.02-6.23)	5.14 (5.04-5.23)
		6.33 (6.19-6.51)	5.35 (5.23-5.50)
	8	7.24 (7.08-7.40)	6.11 (5.97-6.26)
		7.51 (7.28-7.79)	6.31 (6.11-6.56)
	9	9.22 (9.01-9.42)	7.62 (7.44-7.80)
		8.94 (8.53-9.39)	7.45 (7.09-7.82)
**MASD:** 3.464; **CIO:** 0.368			

Relationship of depression and anxiety symptoms with loneliness. Percentile-based 95% bootstrap confidence intervals are reported for original data results. Median and percentile-based 95% confidence intervals of synthetic data results are printed in orange. PHQ-9, nine-item Patient Health Questionnaire; GAD-7, Generalised Anxiety Disorder seven-item scale; MASD, mean absolute standardised difference; CIO, confidence interval overlap

Figure S11. Replication of Figure 2

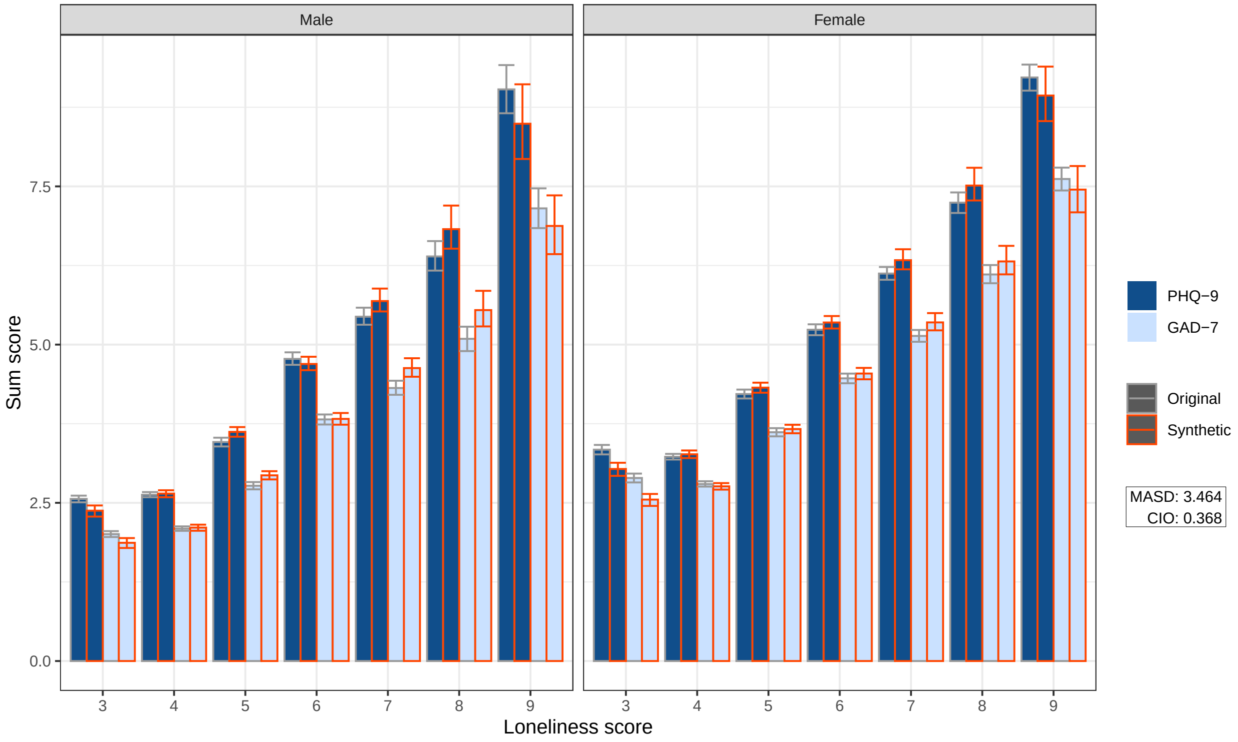

Relationship of depression and anxiety symptoms with loneliness. Percentile-based 95% bootstrap confidence intervals are reported for original data results. Median and percentile-based 95% confidence intervals of synthetic data results are reported. PHQ-9, 9-item Patient Health Questionnaire; GAD-7, Generalised Anxiety Disorder seven-item scale; MASD, mean absolute standardised difference; CIO, confidence interval overlap

Table S20. Replication of Figure 3

Sex	Lonely	PHQ-9 score baseline examination (mean)	GAD-7 score baseline examination (mean)
Male	Yes	4.17 (4.10-4.24)	3.37 (3.31-3.42)
		4.19 (4.11-4.28)	3.42 (3.35-3.49)
	No	2.90 (2.87-2.93)	2.30 (2.27-2.32)
		2.93 (2.89-2.97)	2.33 (2.30-2.36)
Female	Yes	4.79 (4.73-4.84)	4.01 (3.96-4.06)
		4.76 (4.68-4.85)	3.98 (3.91-4.06)
	No	3.73 (3.69-3.76)	3.08 (3.05-3.11)
		3.66 (3.61-3.71)	3.01 (2.96-3.05)
**MASD:** 2.157; **CIO:** 0.542			

Baseline PHQ-9 and GAD-7 sum scores for lonely and non-lonely participants during the pandemic. Percentile-based 95% bootstrap confidence intervals are reported for original data results. Median and percentile-based 95% confidence intervals of synthetic data results are printed in orange. PHQ-9, nine-item Patient Health Questionnaire; GAD-7, Generalised Anxiety Disorder seven-item scale; MASD, mean absolute standardised difference; CIO, confidence interval overlap

Figure S12. Replication of Figure 3

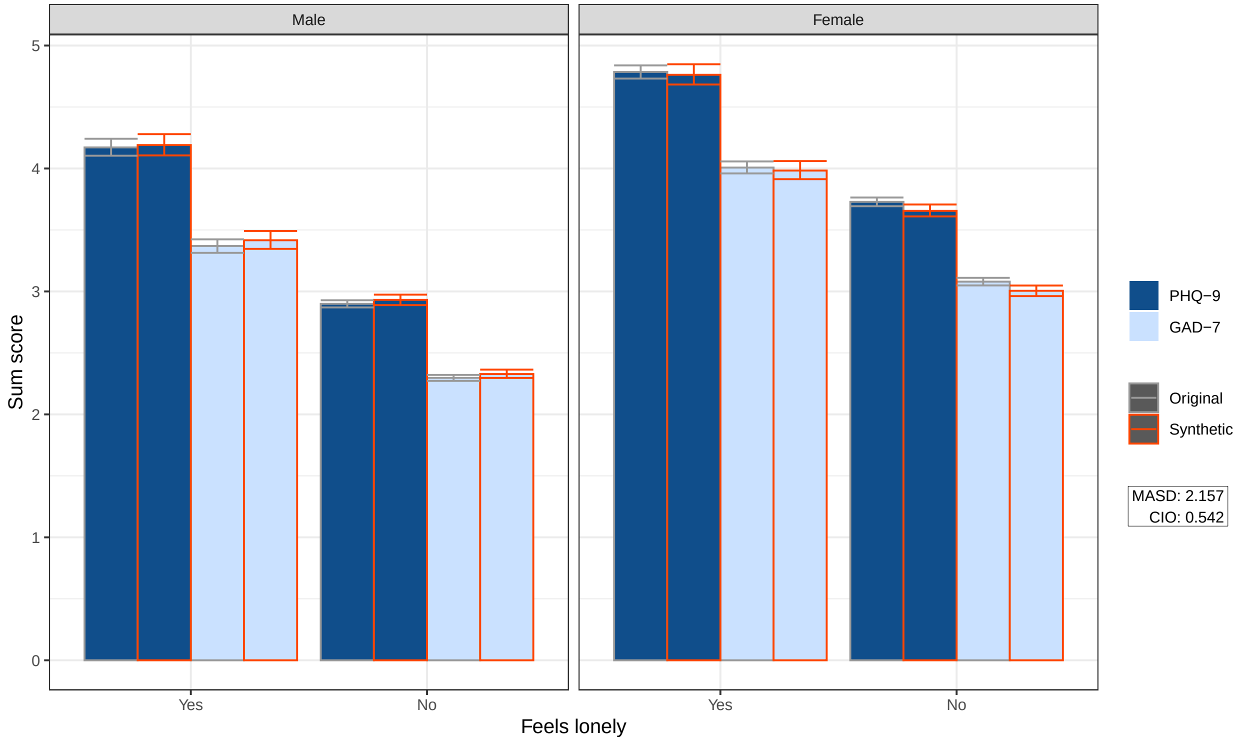

Baseline PHQ-9 and GAD-7 sum scores for lonely and non-lonely participants during the pandemic. Percentile-based 95% bootstrap confidence intervals are reported for original data results. Median and percentile-based 95% confidence intervals of synthetic data results are reported. PHQ-9, nine-item Patient Health Questionnaire; GAD-7, Generalised Anxiety Disorder seven-item scale; MASD, mean absolute standardised difference; CIO, confidence interval overlap

B.6 Tanoey et al.[9]

Table S21. Replication of Table 1 (general and task-specific synthesis)

Variable	Agg.	No diabetes	Diabetes type 1	Diabetes type 2	Total
Number of participants (n)		94408 (94252-94569)	371 (331-410)	6574 (6420-6724)	101353 (101324-101381)
		94407 (94250-94562)	311 (270-351)	5847 (5477-6188)	100576 (100217-100884)
		94411 (94252-94563)	371 (333-412)	6571 (6429-6727)	101353 (101353-101353)
Sex: Male	n	43244 (42932-43555)	204 (175-233)	3602 (3489-3719)	47050 (46735-47362)
		43267 (42956-43586)	156 (130-183)	3164 (2942-3397)	46587 (46222-46949)
		43264 (42953-43555)	199 (171-227)	3588 (3477-3707)	47046 (46737-47346)
	Prop.	0.46 (0.45-0.46)	0.55 (0.50-0.60)	0.55 (0.54-0.56)	0.46 (0.46-0.47)
		0.46 (0.46-0.46)	0.50 (0.44-0.56)	0.54 (0.53-0.56)	0.46 (0.46-0.47)
		0.46 (0.46-0.46)	0.54 (0.49-0.59)	0.55 (0.53-0.56)	0.46 (0.46-0.47)
Sex: Female	n	51164 (50840-51477)	167 (142-193)	2972 (2867-3078)	54303 (53988-54614)
		51143 (50819-51446)	155 (126-183)	2683 (2494-2843)	53972 (53619-54321)
		51151 (50858-51473)	172 (147-198)	2983 (2874-3090)	54307 (54007-54616)
	Prop.	0.54 (0.54-0.55)	0.45 (0.40-0.50)	0.45 (0.44-0.46)	0.54 (0.53-0.54)
		0.54 (0.54-0.54)	0.50 (0.44-0.56)	0.46 (0.44-0.47)	0.54 (0.53-0.54)
		0.54 (0.54-0.54)	0.46 (0.41-0.51)	0.45 (0.44-0.47)	0.54 (0.53-0.54)
Caesarean delivery: No	n	72773 (72491-73049)	259 (226-292)	4290 (4167-4413)	77322 (77051-77582)
		72764 (72508-73046)	221 (188-254)	3760 (3521-4011)	76748 (76410-77083)
		72778 (7244-73048)	260 (231-292)	4288 (4166-4415)	77325 (77058-77591)
	Prop.	0.77 (0.77-0.77)	0.70 (0.65-0.74)	0.65 (0.64-0.66)	0.76 (0.76-0.77)
		0.77 (0.77-0.77)	0.71 (0.65-0.76)	0.64 (0.63-0.66)	0.76 (0.76-0.77)
		0.77 (0.77-0.77)	0.70 (0.66-0.75)	0.65 (0.64-0.66)	0.76 (0.76-0.77)
Caesarean delivery: Yes	n	3485 (3374-3600)	21 (13-30)	105 (86-125)	3611 (3498-3732)
		3481 (3376-3591)	14 (7-22)	90 (70-111)	3585 (3476-3695)
		3480 (3370-3595)	19 (11-29)	109 (89-130)	3609 (3497-3728)
	Prop.	0.04 (0.04-0.04)	0.06 (0.03-0.08)	0.02 (0.01-0.02)	0.04 (0.03-0.04)
		0.04 (0.04-0.04)	0.05 (0.02-0.07)	0.02 (0.01-0.02)	0.04 (0.03-0.04)
		0.04 (0.04-0.04)	0.05 (0.03-0.08)	0.02 (0.01-0.02)	0.04 (0.03-0.04)
Caesarean delivery: Unknown	n	18150 (17906-18390)	91 (73-111)	2179 (2093-2268)	20420 (20168-20671)
		18164 (17923-18379)	76 (58-95)	1999 (1809-2123)	20224 (19950-20491)
		18156 (17918-18382)	90 (72-109)	2174 (2082-2269)	20418 (20178-20659)
	Prop.	0.19 (0.19-0.19)	0.25 (0.20-0.29)	0.33 (0.32-0.34)	0.20 (0.20-0.20)
		0.19 (0.19-0.19)	0.24 (0.19-0.30)	0.34 (0.32-0.36)	0.20 (0.20-0.20)
		0.19 (0.19-0.19)	0.24 (0.20-0.29)	0.33 (0.32-0.34)	0.20 (0.20-0.20)
Birth order: Only child	n	14305 (14085-14523)	69 (52-86)	917 (860-975)	15291 (15056-15511)
		11534 (10969-12209)	36 (24-49)	560 (493-637)	12126 (11551-12846)
		14308 (14089-14522)	67 (51-84)	917 (856-977)	15290 (15078-15514)
	Prop.	0.15 (0.15-0.15)	0.19 (0.15-0.23)	0.14 (0.13-0.15)	0.15 (0.15-0.15)
		0.12 (0.12-0.13)	0.11 (0.08-0.15)	0.10 (0.09-0.11)	0.12 (0.11-0.13)
		0.15 (0.15-0.15)	0.18 (0.14-0.22)	0.14 (0.13-0.15)	0.15 (0.15-0.15)
Birth order: First	n	24751 (24483-25020)	99 (80-119)	1434 (1360-1511)	26284 (26013-26553)
		25218 (24427-25944)	84 (65-103)	1296 (1178-1405)	26592 (25810-27362)
		24753 (24484-25030)	98 (80-117)	1435 (1362-1509)	26287 (26014-26571)
	Prop.	0.26 (0.26-0.27)	0.27 (0.22-0.31)	0.22 (0.21-0.23)	0.26 (0.26-0.26)
		0.27 (0.26-0.27)	0.27 (0.22-0.32)	0.22 (0.21-0.24)	0.26 (0.26-0.27)
		0.26 (0.26-0.27)	0.26 (0.22-0.31)	0.22 (0.21-0.23)	0.26 (0.26-0.26)
Birth order: Second	n	24217 (23942-24490)	81 (64-100)	1299 (1226-1368)	25597 (25325-25867)
		24008 (23307-24459)	73 (55-91)	1184 (1081-1288)	25267 (24533-25752)
		24213 (23955-24476)	82 (65-100)	1300 (1233-1372)	25596 (25337-25858)
	Prop.	0.26 (0.25-0.26)	0.22 (0.18-0.26)	0.20 (0.19-0.21)	0.25 (0.25-0.26)
		0.25 (0.25-0.26)	0.24 (0.19-0.29)	0.20 (0.19-0.22)	0.25 (0.24-0.26)
		0.26 (0.25-0.26)	0.22 (0.18-0.26)	0.20 (0.19-0.21)	0.25 (0.25-0.26)
Birth order: Third or more	n	16105 (15873-16346)	50 (36-65)	1010 (949-1074)	17165 (16924-17408)
		18669 (17847-19291)	56 (41-74)	1030 (922-1130)	19754 (18878-20435)
		16101 (15878-16340)	52 (38-67)	1007 (942-1074)	17158 (16937-17401)
	Prop.	0.17 (0.17-0.17)	0.13 (0.10-0.17)	0.15 (0.14-0.16)	0.17 (0.17-0.17)
		0.20 (0.19-0.20)	0.18 (0.14-0.23)	0.18 (0.16-0.19)	0.20 (0.19-0.20)
		0.17 (0.17-0.17)	0.14 (0.10-0.18)	0.15 (0.14-0.16)	0.17 (0.17-0.17)
Birth order: Missing	n	15030 (14804-15252)	72 (56-89)	1914 (1833-1996)	17016 (16782-17259)
		15032 (14809-15249)	62 (46-79)	1777 (1604-1889)	16865 (16606-17119)
		15031 (14806-15245)	72 (56-90)	1911 (1828-1997)	17015 (16778-17240)
	Prop.	0.16 (0.16-0.16)	0.19 (0.16-0.24)	0.29 (0.28-0.30)	0.17 (0.17-0.17)
		0.16 (0.16-0.16)	0.20 (0.16-0.25)	0.30 (0.28-0.32)	0.17 (0.17-0.17)
		0.16 (0.16-0.16)	0.19 (0.16-0.24)	0.29 (0.28-0.30)	0.17 (0.17-0.17)
Attended daycare: No	n	25396 (25124-25658)	77 (60-94)	2081 (1996-2169)	27554 (27276-27822)
		25400 (25145-25658)	70 (55-88)	1809 (1685-1948)	27281 (26997-27571)
		25402 (25144-25671)	80 (62-97)	2073 (1985-2161)	27554 (27275-27834)
	Prop.	0.27 (0.27-0.27)	0.21 (0.17-0.25)	0.32 (0.31-0.33)	0.27 (0.27-0.27)
		0.27 (0.27-0.27)	0.23 (0.18-0.28)	0.31 (0.30-0.32)	0.27 (0.27-0.27)
		0.27 (0.27-0.27)	0.22 (0.17-0.26)	0.32 (0.30-0.33)	0.27 (0.27-0.27)
Attended daycare: Yes	n	54056 (53735-54376)	222 (191-251)	2596 (2494-2691)	56874 (56555-57192)
		54052 (53757-54365)	179 (151-208)	2270 (2104-2450)	56502 (56177-56842)
		54046 (53756-54355)	219 (191-249)	2603 (2510-2702)	56874 (56573-57183)
	Prop.	0.57 (0.57-0.58)	0.60 (0.55-0.65)	0.39 (0.38-0.41)	0.56 (0.56-0.56)
		0.57 (0.57-0.58)	0.58 (0.52-0.63)	0.39 (0.37-0.40)	0.56 (0.56-0.56)
		0.57 (0.57-0.58)	0.59 (0.54-0.64)	0.40 (0.38-0.41)	0.56 (0.56-0.56)
Attended daycare: Unknown	n	14956 (14734-15182)	72 (56-89)	1897 (1817-1978)	16925 (16697-17165)
		14955 (14733-15167)	61 (45-78)	1766 (1590-1878)	16776 (16519-17027)
		14957 (14742-15169)	72 (55-89)	1895 (1811-1982)	16924 (16695-17150)
	Prop.	0.16 (0.16-0.16)	0.19 (0.16-0.24)	0.29 (0.28-0.30)	0.17 (0.16-0.17)
		0.16 (0.16-0.16)	0.20 (0.15-0.24)	0.30 (0.28-0.32)	0.17 (0.16-0.17)
		0.16 (0.16-0.16)	0.19 (0.16-0.24)	0.29 (0.28-0.30)	0.17 (0.16-0.17)
Birth year group: <=1955	n	28968 (28700-29239)	73 (56-90)	4142 (4017-4266)	33183 (32911-33464)
		27999 (27347-28514)	67 (50-85)	3637 (3373-3849)	31708 (31016-32191)
		29037 (28743-29334)	81 (63-103)	4064 (3912-4207)	33183 (32890-33475)
	Prop.	0.31 (0.30-0.31)	0.20 (0.16-0.24)	0.63 (0.62-0.64)	0.33 (0.32-0.33)
		0.30 (0.29-0.30)	0.22 (0.17-0.27)	0.62 (0.60-0.64)	0.32 (0.31-0.32)
		0.31 (0.30-0.31)	0.22 (0.17-0.28)	0.62 (0.60-0.63)	0.33 (0.32-0.33)
Birth year group: 1956-1965	n	27320 (27049-27597)	118 (97-140)	1611 (1534-1690)	29049 (28768-29340)
		28028 (273767-28731)	100 (79-122)	1498 (1375-1622)	29629 (28952-30373)
		27303 (27036-27588)	117 (95-139)	1624 (1540-1716)	29049 (28774-29336)
	Prop.	0.29 (0.29-0.29)	0.32 (0.27-0.37)	0.25 (0.23-0.26)	0.29 (0.28-0.29)
		0.30 (0.29-0.30)	0.32 (0.27-0.38)	0.26 (0.24-0.27)	0.29 (0.29-0.30)
		0.29 (0.29-0.29)	0.32 (0.27-0.37)	0.25 (0.24-0.26)	0.29 (0.28-0.29)
Birth year group: 1966-1975	n	21574 (21323-21833)	97 (78-117)	619 (571-669)	22290 (22040-22553)
		21517 (21049-21946)	85 (67-105)	535 (476-595)	22133 (21665-22588)
		21548 (21278-21811)	94 (76-113)	648 (593-713)	22288 (22029-22554)
	Prop.	0.23 (0.23-0.23)	0.26 (0.22-0.31)	0.09 (0.09-0.10)	0.22 (0.22-0.22)
		0.23 (0.22-0.23)	0.27 (0.23-0.33)	0.09 (0.08-0.10)	0.22 (0.22-0.22)
		0.23 (0.23-0.23)	0.25 (0.21-0.30)	0.10 (0.09-0.11)	0.22 (0.22-0.22)
Birth year group: 1976-1985	n	9288 (9098-9469)	45 (32-58)	175 (149-202)	9508 (9318-9690)

		10250 (9824-10698)	41 (28-55)	155 (126-185)	10446 (10016-10897)
		9279 (9102-9458)	43 (31-57)	190 (159-225)	9513 (9331-9692)
	Prop.	0.10 (0.10-0.10)	0.12 (0.09-0.16)	0.03 (0.02-0.03)	0.09 (0.09-0.10)
		0.11 (0.10-0.11)	0.13 (0.09-0.17)	0.03 (0.02-0.03)	0.10 (0.10-0.11)
		0.10 (0.10-0.10)	0.12 (0.09-0.15)	0.03 (0.02-0.03)	0.09 (0.09-0.10)
Birth year group: >=1986	n	7258 (7105-7425)	38 (26-50)	27 (18-38)	7323 (7168-7491)
		6612 (6326-6903)	17 (9-26)	23 (14-34)	6654 (6369-6943)
		7248 (7072-7393)	34 (23-47)	42 (27-64)	7324 (7151-7477)
	Prop.	0.08 (0.08-0.08)	0.10 (0.07-0.13)	0.00 (0.00-0.01)	0.07 (0.07-0.07)
		0.07 (0.07-0.07)	0.05 (0.03-0.08)	0.00 (0.00-0.01)	0.07 (0.06-0.07)
		0.08 (0.07-0.08)	0.09 (0.06-0.12)	0.01 (0.00-0.01)	0.07 (0.07-0.07)
Birth year group: Unknown	n	0 (0-0)	0 (0-0)	0 (0-0)	0 (0-0)
		1 (0-4)	0 (0-0)	0 (0-1)	1 (0-4)
		0 (0-0)	0 (0-0)	0 (0-0)	0 (0-0)
	Prop.	0.00 (0.00-0.00)	0.00 (0.00-0.00)	0.00 (0.00-0.00)	0.00 (0.00-0.00)
		0.00 (0.00-0.00)	0.00 (0.00-0.00)	0.00 (0.00-0.00)	0.00 (0.00-0.00)
		0.00 (0.00-0.00)	0.00 (0.00-0.00)	0.00 (0.00-0.00)	0.00 (0.00-0.00)
Paternal diabetes: No	n	60340 (60021-60654)	195 (168-223)	2614 (2513-2719)	63149 (62833-63457)
		60281 (59971-60585)	157 (130-184)	2315 (2165-2479)	62748 (62408-63079)
		60302 (59998-60611)	194 (169-223)	2642 (2541-2748)	63139 (62842-63443)
	Prop.	0.64 (0.64-0.64)	0.53 (0.48-0.58)	0.40 (0.39-0.41)	0.62 (0.62-0.63)
		0.64 (0.64-0.64)	0.50 (0.45-0.56)	0.40 (0.38-0.41)	0.62 (0.62-0.63)
		0.64 (0.64-0.64)	0.53 (0.48-0.58)	0.40 (0.39-0.42)	0.62 (0.62-0.63)
Paternal diabetes: Yes, age <40	n	457 (415-498)	12 (6-19)	63 (48-79)	532 (487-575)
		461 (420-506)	8 (3-15)	55 (41-70)	525 (480-573)
		460 (417-503)	11 (5-18)	61 (47-78)	533 (488-579)
	Prop.	0.00 (0.00-0.01)	0.03 (0.02-0.05)	0.01 (0.01-0.01)	0.01 (0.00-0.01)
		0.00 (0.00-0.01)	0.03 (0.01-0.05)	0.01 (0.01-0.01)	0.01 (0.00-0.01)
		0.00 (0.00-0.01)	0.03 (0.01-0.05)	0.01 (0.01-0.01)	0.01 (0.00-0.01)
Paternal diabetes: Yes, age 40+/NA	n	10630 (10433-10821)	66 (49-82)	1201 (1133-1269)	11897 (11693-12097)
		10672 (10481-10861)	60 (45-77)	1041 (947-1130)	11773 (11565-11965)
		10653 (10450-10847)	66 (51-82)	1180 (1114-1253)	11899 (11694-12109)
	Prop.	0.11 (0.11-0.11)	0.18 (0.14-0.22)	0.18 (0.17-0.19)	0.12 (0.12-0.12)
		0.11 (0.11-0.12)	0.19 (0.15-0.24)	0.18 (0.17-0.19)	0.12 (0.12-0.12)
		0.11 (0.11-0.11)	0.18 (0.14-0.22)	0.18 (0.17-0.19)	0.12 (0.12-0.12)
Paternal diabetes: Unknown	n	22981 (22714-23255)	98 (79-118)	2696 (2593-2792)	25775 (25496-26053)
		22994 (22723-23247)	86 (67-106)	2436 (2226-2589)	25510 (25202-25823)
		22993 (22745-23249)	99 (80-120)	2686 (2589-2789)	25779 (25517-26047)
	Prop.	0.24 (0.24-0.25)	0.26 (0.22-0.31)	0.41 (0.40-0.42)	0.25 (0.25-0.26)
		0.24 (0.24-0.25)	0.28 (0.23-0.33)	0.42 (0.40-0.43)	0.25 (0.25-0.26)
		0.24 (0.24-0.25)	0.27 (0.22-0.31)	0.41 (0.40-0.42)	0.25 (0.25-0.26)
Maternal diabetes: No	n	65979 (65692-66280)	218 (189-247)	2620 (2523-2718)	68817 (68539-69106)
		65901 (65598-66207)	169 (143-198)	2328 (2177-2488)	68398 (68061-68754)
		65939 (65649-66252)	217 (189-247)	2659 (2558-2763)	68822 (68531-69122)
	Prop.	0.70 (0.70-0.70)	0.59 (0.54-0.64)	0.40 (0.39-0.41)	0.68 (0.68-0.68)
		0.70 (0.70-0.70)	0.54 (0.49-0.60)	0.40 (0.38-0.41)	0.68 (0.68-0.68)
		0.70 (0.70-0.70)	0.59 (0.53-0.64)	0.40 (0.39-0.42)	0.68 (0.68-0.68)
Maternal diabetes: Yes, age <40	n	403 (362-443)	13 (6-20)	94 (76-114)	510 (463-556)
		411 (370-451)	10 (5-17)	79 (60-98)	500 (456-544)
		409 (369-447)	12 (5-19)	90 (73-109)	511 (468-552)
	Prop.	0.00 (0.00-0.00)	0.04 (0.02-0.06)	0.01 (0.01-0.02)	0.01 (0.00-0.01)
		0.00 (0.00-0.00)	0.03 (0.02-0.06)	0.01 (0.01-0.02)	0.00 (0.00-0.01)
		0.00 (0.00-0.00)	0.03 (0.01-0.05)	0.01 (0.01-0.02)	0.01 (0.00-0.01)
Maternal diabetes: Yes, age 40+/NA	n	11112 (10921-11306)	63 (48-78)	1679 (1599-1756)	12854 (12652-13055)
		11170 (10969-11375)	64 (48-81)	1448 (1327-1568)	12680 (12465-12897)
		11142 (10934-11333)	64 (49-80)	1647 (1565-1729)	12854 (12634-13062)
	Prop.	0.12 (0.12-0.12)	0.17 (0.13-0.21)	0.26 (0.25-0.27)	0.13 (0.12-0.13)
		0.12 (0.12-0.12)	0.21 (0.16-0.25)	0.25 (0.24-0.26)	0.13 (0.12-0.13)
		0.12 (0.12-0.12)	0.17 (0.13-0.21)	0.25 (0.24-0.26)	0.13 (0.12-0.13)
Maternal diabetes: Unknown	n	16914 (16693-17155)	77 (61-95)	2181 (2090-2270)	19172 (18937-19425)
		16924 (16701-17153)	67 (51-84)	1992 (1810-2116)	18976 (18705-19239)
		16920 (16686-17147)	78 (62-96)	2175 (2083-2267)	19177 (18931-19416)
	Prop.	0.18 (0.18-0.18)	0.21 (0.17-0.25)	0.33 (0.32-0.34)	0.19 (0.19-0.19)
		0.18 (0.18-0.18)	0.22 (0.17-0.26)	0.34 (0.32-0.35)	0.19 (0.19-0.19)
		0.18 (0.18-0.18)	0.21 (0.17-0.25)	0.33 (0.32-0.34)	0.19 (0.19-0.19)
Migration background: No	n	79470 (79208-79721)	330 (295-366)	5374 (5236-5517)	85174 (84943-85406)
		79424 (79159-79673)	265 (229-301)	4792 (4488-5076)	84472 (84096-84816)
		79471 (79219-79714)	327 (292-364)	5376 (5242-5512)	85174 (84952-85394)
	Prop.	0.84 (0.84-0.84)	0.89 (0.86-0.92)	0.82 (0.81-0.83)	0.84 (0.84-0.84)
		0.84 (0.84-0.84)	0.85 (0.81-0.89)	0.82 (0.81-0.83)	0.84 (0.84-0.84)
		0.84 (0.84-0.84)	0.88 (0.85-0.91)	0.82 (0.81-0.83)	0.84 (0.84-0.84)
Migration background: Yes	n	14926 (14704-15153)	41 (29-54)	1199 (1133-1261)	16166 (15934-16398)
		14907 (14686-15132)	45 (31-59)	1040 (946-1128)	15990 (15750-16223)
		14929 (14717-15142)	44 (31-57)	1194 (1130-1261)	16166 (15946-16389)
	Prop.	0.16 (0.16-0.16)	0.11 (0.08-0.14)	0.18 (0.17-0.19)	0.16 (0.16-0.16)
		0.16 (0.16-0.16)	0.14 (0.10-0.18)	0.18 (0.17-0.19)	0.16 (0.16-0.16)
		0.16 (0.16-0.16)	0.12 (0.09-0.15)	0.18 (0.17-0.19)	0.16 (0.16-0.16)
Migration background: Missing	n	12 (6-19)	0 (0-0)	1 (0-3)	13 (7-20)
		81 (44-117)	1 (0-4)	16 (7-28)	98 (61-133)
		12 (6-19)	0 (0-0)	1 (0-3)	13 (7-20)
	Prop.	0.00 (0.00-0.00)	0.00 (0.00-0.00)	0.00 (0.00-0.00)	0.00 (0.00-0.00)
		0.00 (0.00-0.00)	0.00 (0.00-0.01)	0.00 (0.00-0.00)	0.00 (0.00-0.00)
		0.00 (0.00-0.00)	0.00 (0.00-0.00)	0.00 (0.00-0.00)	0.00 (0.00-0.00)
Prematural birth: No	n	72161 (71887-72439)	270 (237-303)	4141 (4021-4261)	76572 (76299-76843)
		72158 (71886-72420)	222 (190-255)	3641 (3412-3888)	76020 (75685-76349)
		72160 (71879-72420)	269 (238-303)	4142 (4024-4267)	76571 (76310-76830)
	Prop.	0.76 (0.76-0.77)	0.73 (0.68-0.77)	0.63 (0.62-0.64)	0.76 (0.75-0.76)
		0.76 (0.76-0.77)	0.72 (0.66-0.77)	0.62 (0.61-0.64)	0.76 (0.75-0.76)
		0.76 (0.76-0.77)	0.73 (0.68-0.77)	0.63 (0.62-0.64)	0.76 (0.75-0.76)
Prematural birth: Yes	n	3177 (3067-3288)	12 (6-19)	183 (158-209)	3372 (3259-3483)
		3174 (3069-3284)	10 (4-17)	150 (122-179)	3335 (3226-3447)
		3177 (3068-3285)	12 (6-19)	183 (158-210)	3373 (3260-3483)
	Prop.	0.03 (0.03-0.03)	0.03 (0.02-0.05)	0.03 (0.02-0.03)	0.03 (0.03-0.03)
		0.03 (0.03-0.03)	0.03 (0.01-0.05)	0.03 (0.02-0.03)	0.03 (0.03-0.03)
		0.03 (0.03-0.03)	0.03 (0.02-0.05)	0.03 (0.02-0.03)	0.03 (0.03-0.03)
Prematural birth: Unknown	n	19070 (18834-19316)	89 (71-107)	2250 (2158-2343)	21409 (21161-21675)
		19076 (18827-19315)	78 (60-96)	2058 (1860-2186)	21205 (20923-21478)
		19077 (18841-19308)	90 (72-109)	2245 (2153-2335)	21409 (21169-21656)
	Prop.	0.20 (0.20-0.20)	0.24 (0.20-0.28)	0.34 (0.33-0.35)	0.21 (0.21-0.21)
		0.20 (0.20-0.20)	0.25 (0.20-0.30)	0.35 (0.33-0.37)	0.21 (0.21-0.21)
		0.20 (0.20-0.20)	0.24 (0.20-0.29)	0.34 (0.33-0.35)	0.21 (0.21-0.21)
Birth weight: Average	n	47915 (47593-48218)	176 (149-202)	2482 (2391-2579)	50573 (50257-50885)
		47903 (47607-48220)	142 (118-167)	2207 (2059-2366)	50256 (49927-50608)
		47908 (47591-48207)	174 (150-200)	2490 (2398-2586)	50574 (50265-50866)
	Prop.	0.51 (0.50-0.51)	0.47 (0.42-0.52)	0.38 (0.37-0.39)	0.50 (0.50-0.50)
		0.51 (0.50-0.51)	0.46 (0.40-0.51)	0.38 (0.36-0.39)	0.50 (0.50-0.50)
		0.51 (0.50-0.51)	0.47 (0.42-0.52)	0.38 (0.37-0.39)	0.50 (0.50-0.50)
Birth weight: Light	n	8014 (7847-8178)	29 (18-40)	510 (465-555)	8553 (8388-8728)

		8020 (7853-8179)	26 (16-36)	432 (380-485)	8477 (8299-8645)
		8021 (7850-8186)	30 (19-41)	506 (462-554)	8557 (8382-8729)
	Prop.	0.08 (0.08-0.09)	0.08 (0.05-0.11)	0.08 (0.07-0.08)	0.08 (0.08-0.09)
		0.08 (0.08-0.09)	0.08 (0.05-0.12)	0.07 (0.07-0.08)	0.08 (0.08-0.09)
		0.08 (0.08-0.09)	0.08 (0.05-0.11)	0.08 (0.07-0.08)	0.08 (0.08-0.09)
Birth weight: Heavy	n	8096 (7927-8265)	34 (23-46)	401 (362-442)	8531 (8353-8701)
		8088 (7920-8260)	27 (17-38)	344 (303-387)	8459 (8288-8636)
		8098 (7924-8267)	33 (22-44)	403 (364-443)	8534 (8357-8713)
	Prop.	0.09 (0.08-0.09)	0.09 (0.06-0.12)	0.06 (0.06-0.07)	0.08 (0.08-0.09)
		0.09 (0.08-0.09)	0.09 (0.06-0.12)	0.06 (0.05-0.07)	0.08 (0.08-0.09)
		0.09 (0.08-0.09)	0.09 (0.06-0.12)	0.06 (0.06-0.07)	0.08 (0.08-0.09)
Birth weight: Unknown	n	30383 (30088-30670)	132 (110-155)	3181 (3072-3289)	33696 (33394-33998)
		30390 (30108-30678)	116 (93-139)	2862 (2635-3036)	33367 (33010-33701)
		30390 (30117-30670)	133 (112-157)	3171 (3063-3283)	33697 (33413-33987)
	Prop.	0.32 (0.32-0.32)	0.36 (0.31-0.40)	0.48 (0.47-0.50)	0.33 (0.33-0.34)
		0.32 (0.32-0.32)	0.37 (0.32-0.43)	0.49 (0.47-0.50)	0.33 (0.33-0.33)
		0.32 (0.32-0.32)	0.36 (0.31-0.41)	0.48 (0.47-0.49)	0.33 (0.33-0.34)
Ever breastfed: No	n	13252 (13054-13467)	60 (46-75)	604 (557-651)	13916 (13709-14131)
		13258 (13041-13460)	51 (38-66)	519 (461-579)	13826 (13607-14037)
		13253 (13043-13463)	60 (44-74)	603 (557-652)	13918 (13705-14126)
	Prop.	0.14 (0.14-0.14)	0.16 (0.12-0.20)	0.09 (0.08-0.10)	0.14 (0.14-0.14)
		0.14 (0.14-0.14)	0.17 (0.13-0.21)	0.09 (0.08-0.10)	0.14 (0.14-0.14)
		0.14 (0.14-0.14)	0.16 (0.12-0.20)	0.09 (0.09-0.10)	0.14 (0.14-0.14)
Ever breastfed: Yes, >4 months	n	18103 (17864-18341)	66 (50-82)	1046 (983-1106)	19215 (18968-19461)
		18101 (17871-18349)	54 (39-68)	911 (836-996)	19066 (18823-19311)
		18110 (17868-18339)	66 (51-82)	1045 (982-1105)	19219 (18976-19454)
	Prop.	0.19 (0.19-0.19)	0.18 (0.14-0.22)	0.16 (0.15-0.17)	0.19 (0.19-0.19)
		0.19 (0.19-0.19)	0.17 (0.13-0.21)	0.16 (0.15-0.17)	0.19 (0.19-0.19)
		0.19 (0.19-0.19)	0.18 (0.14-0.22)	0.16 (0.15-0.17)	0.19 (0.19-0.19)
Ever breastfed: Yes, until 4 months	n	21341 (21093-21597)	79 (62-96)	1200 (1135-1268)	22620 (22363-22890)
		21341 (21091-21593)	64 (48-81)	1055 (966-1148)	22458 (22193-22736)
		21339 (21090-21587)	78 (62-96)	1202 (1136-1268)	22621 (22371-22875)
	Prop.	0.23 (0.22-0.23)	0.21 (0.17-0.26)	0.18 (0.17-0.19)	0.22 (0.22-0.23)
		0.23 (0.22-0.23)	0.21 (0.16-0.25)	0.18 (0.17-0.19)	0.22 (0.22-0.23)
		0.23 (0.22-0.23)	0.21 (0.17-0.25)	0.18 (0.17-0.19)	0.22 (0.22-0.23)
Ever breastfed: Unknown	n	41712 (41413-42016)	166 (141-193)	3724 (3601-3836)	45602 (45291-45914)
		41706 (41424-42018)	142 (116-168)	3357 (3113-3562)	45206 (44857-45557)
		41709 (41414-41997)	167 (143-193)	3722 (3604-3845)	45599 (45305-45893)
	Prop.	0.44 (0.44-0.44)	0.45 (0.40-0.50)	0.57 (0.55-0.58)	0.45 (0.45-0.45)
		0.44 (0.44-0.45)	0.46 (0.40-0.51)	0.57 (0.56-0.59)	0.45 (0.45-0.45)
		0.44 (0.44-0.44)	0.45 (0.40-0.50)	0.57 (0.55-0.58)	0.45 (0.45-0.45)
BMI at age 18: Underweight	n	8847 (8682-9029)	24 (15-34)	400 (360-441)	9271 (9099-9456)
		9449 (9119-9855)	21 (12-32)	349 (304-398)	9823 (9471-10244)
		8838 (8667-9021)	25 (16-35)	403 (364-443)	9267 (9098-9445)
	Prop.	0.09 (0.09-0.10)	0.06 (0.04-0.09)	0.06 (0.05-0.07)	0.09 (0.09-0.09)
		0.10 (0.10-0.10)	0.07 (0.04-0.10)	0.06 (0.05-0.07)	0.10 (0.09-0.10)
		0.09 (0.09-0.10)	0.07 (0.04-0.09)	0.06 (0.06-0.07)	0.09 (0.09-0.09)
BMI at age 18: Normal weight	n	50115 (49800-50429)	173 (147-199)	2519 (2423-2610)	52807 (52475-53126)
		47948 (47165-48571)	127 (104-152)	2138 (1990-2280)	50208 (49483-50844)
		50099 (49785-50399)	172 (146-199)	2536 (2436-2631)	52808 (52493-53118)
	Prop.	0.53 (0.53-0.53)	0.47 (0.41-0.52)	0.38 (0.37-0.39)	0.52 (0.52-0.52)
		0.51 (0.50-0.51)	0.41 (0.35-0.46)	0.37 (0.35-0.38)	0.50 (0.49-0.51)
		0.53 (0.53-0.53)	0.46 (0.41-0.52)	0.39 (0.37-0.40)	0.52 (0.52-0.52)
BMI at age 18: Overweight	n	5663 (5523-5804)	31 (21-42)	567 (520-616)	6261 (6111-6408)
		6920 (6559-7372)	35 (24-47)	550 (488-617)	7503 (7127-7990)
		5677 (5532-5815)	31 (21-43)	556 (511-602)	6265 (6114-6408)
	Prop.	0.06 (0.06-0.06)	0.08 (0.06-0.11)	0.09 (0.08-0.09)	0.06 (0.06-0.06)
		0.07 (0.07-0.08)	0.11 (0.08-0.15)	0.09 (0.09-0.10)	0.07 (0.07-0.08)
		0.06 (0.06-0.06)	0.08 (0.06-0.11)	0.08 (0.08-0.09)	0.06 (0.06-0.06)
BMI at age 18: Obese	n	209 (135-274)	3 (0-7)	25 (15-40)	237 (156-313)
		365 (286-464)	3 (0-7)	39 (24-55)	407 (320-517)
		210 (183-241)	2 (0-6)	24 (15-34)	237 (209-269)
	Prop.	0.00 (0.00-0.00)	0.01 (0.00-0.02)	0.00 (0.00-0.01)	0.00 (0.00-0.00)
		0.00 (0.00-0.00)	0.01 (0.00-0.02)	0.01 (0.00-0.01)	0.00 (0.00-0.01)
		0.00 (0.00-0.00)	0.01 (0.00-0.02)	0.00 (0.00-0.01)	0.00 (0.00-0.00)
BMI at age 18: Missing	n	29574 (29277-29870)	140 (116-164)	3063 (2959-3164)	32777 (32468-33087)
		29721 (29424-30003)	123 (100-148)	2772 (2548-2943)	32605 (32277-32925)
		29585 (29295-29870)	140 (117-164)	3052 (2946-3162)	32778 (32482-33074)
	Prop.	0.31 (0.31-0.32)	0.38 (0.33-0.43)	0.47 (0.45-0.48)	0.32 (0.32-0.33)
		0.31 (0.31-0.32)	0.40 (0.34-0.46)	0.47 (0.46-0.49)	0.32 (0.32-0.33)
		0.31 (0.31-0.32)	0.38 (0.33-0.43)	0.46 (0.45-0.48)	0.32 (0.32-0.33)

General: **MASD:** 4.539; **CIO:** 0.602
Task-specific: **MASD:** 0.160; **CIO:** 0.951

Cohort description by diabetes category. Percentile-based 95% bootstrap confidence intervals are reported for original data results. Median and percentile-based 95% confidence intervals of synthetic data results are reported, general synthesis replication results are printed in orange, task-specific synthesis replication results in violet. agg., aggregation; n, number of participants; prop., proportion; NA, indicator for missing values; BMI, body mass index; MASD, mean absolute standardised difference; CIO, confidence interval overlap

Table S22. Replication of Table 2

Variable	Model	Age at diagnosis	Hazard ratio	2.5%	97.5%
Birth order: First	Univariable	0–40	0.83 (0.61-1.14)	0.61 (0.45-0.82)	1.12 (0.83-1.57)
			1.06 (0.72-1.63)	0.72 (0.49-1.04)	1.58 (1.05-2.56)
Birth order: Second			0.69 (0.50-0.96)	0.50 (0.36-0.69)	0.94 (0.69-1.34)
			0.97 (0.65-1.51)	0.65 (0.44-0.98)	1.44 (0.95-2.37)
Birth order: >=Third			0.66 (0.45-0.96)	0.46 (0.30-0.67)	0.95 (0.66-1.38)
			0.97 (0.63-1.51)	0.63 (0.41-0.96)	1.48 (0.95-2.39)
Birth order: Unknown			1.07 (0.76-1.50)	0.77 (0.54-1.07)	1.49 (1.06-2.12)
			1.34 (0.89-2.10)	0.89 (0.59-1.34)	2.04 (1.33-3.39)
C-section delivery: Yes			1.51 (0.88-2.24)	0.96 (0.50-1.53)	2.37 (1.56-3.30)
			1.32 (0.69-2.13)	0.77 (0.33-1.37)	2.27 (1.42-3.35)
C-section delivery: Unknown			1.48 (1.14-1.87)	1.16 (0.89-1.48)	1.89 (1.48-2.36)
			1.40 (1.04-1.82)	1.07 (0.78-1.42)	1.82 (1.38-2.34)
Attended daycare: Yes			1.12 (0.86-1.49)	0.85 (0.66-1.11)	1.48 (1.13-2.01)
			1.08 (0.81-1.47)	0.81 (0.61-1.08)	1.45 (1.06-2.01)
Attended Daycare: Unknown			1.50 (1.08-2.07)	1.08 (0.78-1.50)	2.07 (1.49-2.86)
			1.40 (0.98-1.96)	0.99 (0.69-1.39)	1.98 (1.39-2.78)
Sex: Female			0.69 (0.57-0.84)	0.56 (0.46-0.69)	0.85 (0.70-1.04)
			0.84 (0.67-1.07)	0.68 (0.53-0.85)	1.06 (0.84-1.33)
Birth order: First	Multivariable full		0.85 (0.63-1.18)	0.63 (0.46-0.85)	1.17 (0.86-1.64)
			1.08 (0.73-1.66)	0.73 (0.50-1.07)	1.61 (1.06-2.63)
Birth order: Second			0.69 (0.50-0.98)	0.50 (0.36-0.70)	0.96 (0.70-1.38)
			0.97 (0.65-1.54)	0.65 (0.44-0.99)	1.46 (0.95-2.41)
Birth order: >=Third			0.65 (0.44-0.94)	0.45 (0.30-0.66)	0.94 (0.65-1.37)
			0.93 (0.59-1.47)	0.61 (0.39-0.93)	1.42 (0.90-2.33)
Birth order: Unknown			0.61 (0.16-2.23)	0.13 (0.05-0.67)	2.91 (0.53-7.56)
			1.10 (0.26-4.05)	0.43 (0.11-1.81)	2.79 (0.58-9.38)
C-section delivery: Yes			1.32 (0.78-1.97)	0.83 (0.44-1.33)	2.08 (1.36-2.94)
			1.22 (0.62-2.00)	0.71 (0.30-1.28)	2.11 (1.32-3.18)
C-section delivery: Unknown			1.46 (0.80-2.36)	0.89 (0.44-1.52)	2.42 (1.46-3.66)
			1.23 (0.57-2.21)	0.73 (0.30-1.39)	2.09 (1.09-3.48)
Attended daycare: Yes			1.05 (0.79-1.41)	0.79 (0.60-1.04)	1.39 (1.05-1.89)
			1.08 (0.80-1.47)	0.80 (0.60-1.08)	1.44 (1.06-2.00)
Attended Daycare: Unknown			1.46 (0.39-6.05)	0.31 (0.11-1.94)	6.92 (1.29-19.35)
			0.97 (0.28-3.73)	0.39 (0.13-1.65)	2.41 (0.58-8.82)
Sex: Female			0.67 (0.55-0.83)	0.54 (0.44-0.67)	0.83 (0.68-1.02)
			0.82 (0.64-1.05)	0.65 (0.51-0.83)	1.03 (0.81-1.31)
Birth order: First	Multivariable reduced		0.85 (0.63-1.17)	0.62 (0.46-0.85)	1.16 (0.86-1.63)
			1.08 (0.73-1.66)	0.73 (0.50-1.06)	1.60 (1.06-2.61)
Birth order: Second			0.69 (0.50-0.98)	0.50 (0.36-0.70)	0.96 (0.70-1.37)
			0.97 (0.65-1.53)	0.65 (0.44-0.99)	1.45 (0.94-2.39)
Birth order: >=Third			0.65 (0.44-0.95)	0.45 (0.30-0.65)	0.94 (0.65-1.37)
			0.93 (0.59-1.49)	0.61 (0.39-0.93)	1.43 (0.90-2.35)
Birth order: Unknown			0.63 (0.17-2.23)	0.13 (0.05-0.69)	3.01 (0.54-7.54)
			1.15 (0.26-4.14)	0.45 (0.12-1.90)	2.88 (0.61-9.55)
C-section delivery: Yes			1.35 (0.79-1.99)	0.85 (0.45-1.34)	2.12 (1.39-2.95)
			1.26 (0.66-2.04)	0.73 (0.31-1.30)	2.17 (1.36-3.24)
C-section delivery: Unknown			1.43 (0.81-2.20)	0.90 (0.45-1.49)	2.29 (1.47-3.29)
			1.30 (0.61-2.24)	0.78 (0.33-1.47)	2.14 (1.16-3.42)
Attended daycare: Yes			1.04 (0.79-1.40)	0.79 (0.60-1.04)	1.38 (1.04-1.88)
			1.07 (0.80-1.46)	0.80 (0.60-1.07)	1.44 (1.05-1.99)
Attended Daycare: Unknown			1.42 (0.36-5.89)	0.30 (0.11-1.95)	6.75 (1.15-18.61)
			1.00 (0.28-3.93)	0.41 (0.13-1.83)	2.49 (0.60-8.96)
Sex: Female			0.68 (0.56-0.83)	0.55 (0.45-0.67)	0.84 (0.69-1.02)
			0.83 (0.66-1.05)	0.67 (0.52-0.84)	1.04 (0.82-1.31)
Birth order: First		0–15	0.66 (0.38-1.15)	0.38 (0.22-0.64)	1.15 (0.67-2.07)
			0.99 (0.40-3.69)	0.39 (0.15-0.90)	2.52 (0.95-15.85)
Birth order: Second			0.54 (0.28-0.97)	0.30 (0.15-0.52)	0.95 (0.52-1.78)
			0.78 (0.29-2.82)	0.29 (0.09-0.69)	2.06 (0.77-12.44)
Birth order: >=Third			0.42 (0.17-0.86)	0.20 (0.06-0.42)	0.87 (0.43-1.73)
			0.57 (0.13-2.46)	0.18 (0.02-0.55)	1.81 (0.63-12.06)
Birth order: Unknown			0.74 (0.41-1.90)	0.03 (0.01-0.13)	20.11 (10.77-45.82)
			1.10 (0.04-22.05)	0.08 (0.00-4.04)	14.37 (0.32-189.11)
C-section delivery: Yes			1.68 (0.69-3.02)	0.85 (0.26-1.70)	3.32 (1.78-5.53)
			1.63 (0.29-4.01)	0.54 (0.04-1.74)	4.88 (2.49-12.12)
C-section delivery: Unknown			0.97 (0.12-2.31)	0.31 (0.01-1.02)	3.03 (1.62-5.30)
			1.08 (0.06-4.24)	0.27 (0.01-1.64)	4.50 (0.45-11.69)
Attended daycare: Yes			0.84 (0.48-1.54)	0.47 (0.28-0.81)	1.48 (0.83-3.02)
			0.98 (0.41-2.83)	0.41 (0.19-0.87)	2.28 (0.92-9.62)
Attended Daycare: Unknown			1.71 (0.92-4.92)	0.06 (0.02-0.30)	47.26 (25.07-108.73)
			1.50 (0.05-31.10)	0.12 (0.00-6.38)	19.00 (0.39-200.59)
Sex: Female			0.82 (0.55-1.22)	0.55 (0.37-0.81)	1.22 (0.84-1.86)
			0.84 (0.44-1.64)	0.46 (0.23-0.84)	1.55 (0.82-3.21)
Birth order: First		16–40	0.95 (0.66-1.43)	0.65 (0.45-0.96)	1.39 (0.95-2.14)
			1.11 (0.72-1.76)	0.71 (0.47-1.07)	1.71 (1.08-2.90)
Birth order: Second			0.78 (0.52-1.18)	0.52 (0.35-0.78)	1.15 (0.77-1.80)
			1.02 (0.67-1.68)	0.66 (0.43-1.03)	1.58 (1.01-2.80)
Birth order: >=Third			0.77 (0.49-1.19)	0.50 (0.31-0.76)	1.19 (0.78-1.88)
			1.02 (0.62-1.72)	0.64 (0.40-1.03)	1.61 (0.97-2.84)
Birth order: Unknown			0.62 (0.13-2.82)	0.11 (0.04-0.81)	3.66 (0.45-10.25)
			1.21 (0.26-4.60)	0.45 (0.11-1.98)	3.26 (0.62-11.10)
C-section delivery: Yes			1.13 (0.53-1.89)	0.61 (0.22-1.15)	2.10 (1.25-3.16)
			1.14 (0.50-1.99)	0.60 (0.20-1.20)	2.18 (1.25-3.36)
C-section delivery: Unknown			1.58 (0.81-2.50)	0.94 (0.41-1.61)	2.65 (1.59-3.88)
			1.30 (0.58-2.33)	0.76 (0.29-1.50)	2.23 (1.18-3.64)
Attended daycare: Yes			1.11 (0.80-1.57)	0.81 (0.58-1.11)	1.54 (1.09-2.22)
			1.09 (0.79-1.51)	0.80 (0.58-1.09)	1.49 (1.07-2.12)
Attended Daycare: Unknown			1.36 (0.28-7.17)	0.23 (0.08-2.20)	7.87 (0.91-24.07)
			0.95 (0.27-4.13)	0.37 (0.12-1.80)	2.49 (0.59-9.75)
Sex: Female			0.63 (0.50-0.80)	0.50 (0.39-0.63)	0.81 (0.64-1.03)
			0.83 (0.64-1.08)	0.65 (0.50-0.85)	1.05 (0.82-1.38)
**MASD:** 1.124; **CIO:** 0.728					

Type 1 diabetes risk estimates from Cox regression models with all cases, onset age 0–15 years (n = 100) and onset age 16–40 years (n = 271). Full models adjusted for paternal diabetes, maternal diabetes, migration background, premature birth, birth weight and being breastfed history. Reduced models adjusted for paternal diabetes, maternal diabetes and migration background. All models were stratified by birth year. Percentile-based 95% bootstrap confidence intervals are reported for original data results. Median and percentile-based 95% confidence intervals of synthetic data results are printed in orange. n, number of participants; 2.5% and 97.5%, regression 95% confidence interval limits; C-section, caesarean section; MASD, mean absolute standardised difference; CIO, confidence interval overlap

Table S23. Replication of univariable Cox regressions - Table 2 (general and task-specific synthesis)

Variable	Hazard ratio	2.5%	97.5%
Birth order: First	0.83 (0.61-1.14)	0.61 (0.45-0.82)	1.12 (0.83-1.57)
	1.06 (0.72-1.63)	0.72 (0.49-1.04)	1.58 (1.05-2.56)
	0.84 (0.62-1.17)	0.62 (0.46-0.85)	1.14 (0.85-1.61)
Birth order: Second	0.69 (0.49-0.96)	0.50 (0.36-0.69)	0.94 (0.68-1.35)
	0.97 (0.65-1.51)	0.65 (0.44-0.98)	1.44 (0.95-2.37)
	0.70 (0.51-0.97)	0.51 (0.37-0.70)	0.97 (0.71-1.35)
Birth order: >=Third	0.66 (0.46-0.95)	0.46 (0.31-0.66)	0.95 (0.67-1.38)
	0.97 (0.63-1.51)	0.63 (0.41-0.96)	1.48 (0.95-2.39)
	0.67 (0.47-0.97)	0.47 (0.32-0.68)	0.97 (0.70-1.39)
Birth order: Unknown	1.07 (0.77-1.52)	0.77 (0.55-1.08)	1.49 (1.08-2.14)
	1.34 (0.89-2.10)	0.89 (0.59-1.34)	2.04 (1.33-3.39)
	1.08 (0.77-1.51)	0.77 (0.54-1.07)	1.50 (1.08-2.12)
C-section delivery: Yes	1.51 (0.89-2.25)	0.96 (0.51-1.51)	2.37 (1.57-3.33)
	1.32 (0.69-2.13)	0.77 (0.33-1.37)	2.27 (1.42-3.35)
	1.52 (0.90-2.34)	0.97 (0.51-1.59)	2.39 (1.60-3.41)
C-section delivery: Unknown	1.48 (1.16-1.87)	1.16 (0.89-1.49)	1.89 (1.50-2.36)
	1.40 (1.04-1.82)	1.07 (0.78-1.42)	1.82 (1.38-2.34)
	1.46 (1.12-1.83)	1.15 (0.86-1.45)	1.86 (1.46-2.30)
Attended daycare: Yes	1.12 (0.84-1.51)	0.85 (0.64-1.13)	1.48 (1.11-2.03)
	1.08 (0.81-1.47)	0.81 (0.61-1.08)	1.45 (1.06-2.01)
	1.18 (0.89-1.57)	0.89 (0.68-1.17)	1.55 (1.16-2.10)
Attended Daycare: Unknown	1.50 (1.08-2.06)	1.08 (0.77-1.49)	2.07 (1.50-2.86)
	1.40 (0.98-1.96)	0.99 (0.69-1.39)	1.98 (1.39-2.78)
	1.53 (1.10-2.11)	1.11 (0.79-1.52)	2.12 (1.54-2.94)
Sex: Female	0.69 (0.56-0.85)	0.56 (0.46-0.69)	0.85 (0.70-1.04)
	0.84 (0.67-1.07)	0.68 (0.53-0.85)	1.06 (0.84-1.33)
	0.70 (0.57-0.87)	0.57 (0.47-0.71)	0.87 (0.71-1.06)
**General:** **MASD:** 1.834; **CIO:** 0.626			
**Task-specific:** **MASD:** 0.130; **CIO:** 0.961			

Type 1 diabetes univariable Cox regression estimates. Percentile-based 95% bootstrap confidence intervals are reported for original data results. Median and percentile-based 95% confidence intervals of synthetic data results are reported, general synthesis replication results are printed in orange, task-specific synthesis replication results in violet. 2.5% and 97.5%, regression 95% confidence interval limits; C-section, caesarean section; MASD, mean absolute standardised difference; CIO, confidence interval overlap

Figure S13. Replication of univariable Cox regressions - Table 2 (general and task-specific synthesis)

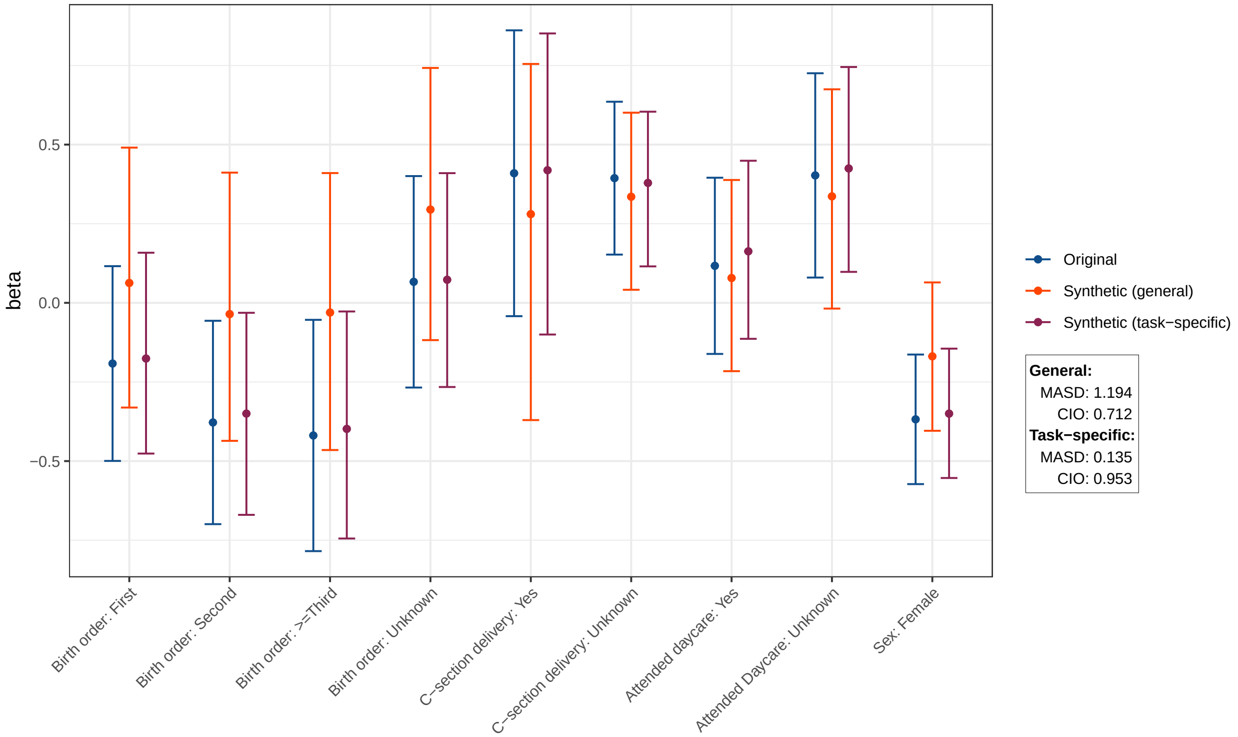

Type 1 diabetes univariable Cox regression estimates. Median beta estimates and percentile-based 95% confidence intervals computed from the beta coefficient distribution across synthesis repetitions are reported for synthetic data. C-section, caesarean section; MASD, mean absolute standardised difference; CIO, confidence interval overlap

Table S24. Replication of Figure 2 (general and task-specific synthesis)

Sex	Age at diagnosis	Therapy	n
Male	0-15	With insulin	36 (25-48)
			15 (8-24)
			42 (28-56)
		Unknown	13 (6-21)
			6 (2-12)
			12 (6-20)
	16-30	With insulin	74 (58-91)
			44 (31-58)
			73 (56-92)
		Unknown	6 (2-11)
			11 (5-18)
			7 (2-13)
	31-40	With insulin	64 (49-80)
			62 (45-80)
			58 (44-76)
		Unknown	11 (5-18)
			17 (9-27)
			10 (4-17)
Female	0-15	With insulin	45 (32-59)
			18 (10-28)
			42 (29-55)
		Unknown	6 (2-11)
			4 (1-8)
			6 (2-12)
	16-30	With insulin	55 (41-70)
			42 (28-56)
			61 (46-80)
		Unknown	5 (1-10)
			15 (8-24)
			5 (1-11)
	31-40	With insulin	46 (33-60)
			51 (37-67)
			42 (30-56)
		Unknown	10 (4-17)
			24 (14-38)
			9 (4-17)
**General:** **MASD:** 2.441; **CIO:** 0.445			
**Task-specific:** **MASD:** 0.417; **CIO:** 0.903			

Numbers of type 1 diabetes cases by sex, age at diagnosis (in years) and therapy. Percentile-based 95% bootstrap confidence intervals are reported for original data results. Median and percentile-based 95% confidence intervals of synthetic data results are reported, general synthesis replication results are printed in orange, task-specific synthesis replication results in violet. n, number of participants; MASD, mean absolute standardised difference; CIO, confidence interval overlap

Figure S14. Replication of Figure 2 (general and task-specific synthesis)

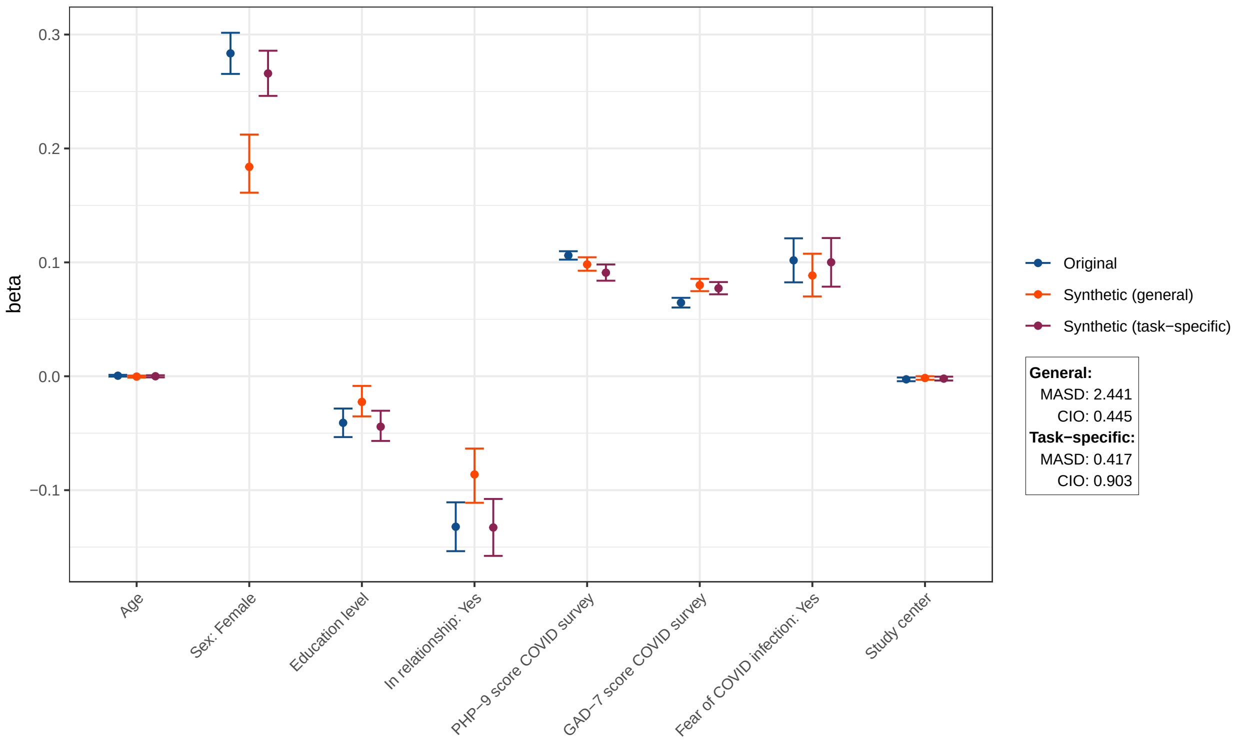

Numbers of participants with type 1 diabetes by sex, age at diagnosis (in years) and therapy. Percentile-based 95% bootstrap confidence intervals are reported for original data results. Median and percentile-based 95% confidence intervals of synthetic data results are reported. n, number of participants; MASD, mean absolute standardised difference; CIO, confidence interval overlap

C Comparative analysis of ARF performance across varying minimum node sizes and alternative synthesizers

The evaluation in the main text focuses on the reproducibility of published epidemiological analyses using ARF-generated synthetic data, providing an interpretable and application-oriented assessment of utility. However, synthetic data quality is inherently multidimensional, and downstream utility represents only one aspect. Important additional dimensions include privacy risk and generalisation to unseen data. In this section, we therefore provide a more comprehensive evaluation of the synthetic data generated by ARF, including an assessment of privacy risk (Supplement C.1) and generalisation to out-of-sample data (Supplement C.2). In addition, we report a runtime analysis of the data synthesis process (Supplement C.3).

In all experiments, we compared results across different values of the ARF minimum node size (2, 5, 10, 20, and 50), with 2 corresponding to the default setting. While random forests are generally considered relatively robust to hyperparameter choices in discriminative modelling,[10] this parameter controlling the granularity of the tree partitions directly influences the utility–privacy trade-off in generative modelling with ARF. In general, despite its potential benefits, we note that systematic hyperparameter optimization is often omitted in generative contexts due to the substantial computational overhead and the complexity of the evaluation process.[11] Results were further compared with five additional synthetic data generators to contextualise the findings and position ARF's performance relative to alternative synthesis approaches. Specifically, we included *synthpop*[12], *Bayesian Networks*[13] and their differentially private[14] variant *PrivBayes*[15], as well as the deep learning–based approaches *CTGAN*[16] and *TVAE*[16]. These represent popular methods for tabular data synthesis, available in well-maintained packages on CRAN or PyPI and able to handle missing data. Recent promising deep learning approaches for tabular data synthesis, such as diffusion-based models[17], do not currently meet these criteria. As both privacy and generalisation must be evaluated on test data excluded from synthesizer training, all experiments in this section were conducted on five different 50/50 train-test splits.

C.1 Utility-privacy trade-off

In this section, the utility dimension was again evaluated by replicating the downstream analyses in Figures 1–5. For descriptive analyses, we report the mean absolute standardised difference (MASD) and the the mean confidence interval overlap (CIO) for regression analyses. We refer to Supplement A for a definition of these metrics. For MASD calcuation, we quantified the standard error of the original estimates again via bootrapping with 2 000 repetitions. The calculation of the CIO was based on the confidence intervals obtained from the regression models. For the distribution analysis in Figure 2, we computed the mean of the Wasserstein distances (WD)[4] between training and synthetic data over all four subgroups. Average values over all five repetitions were reported.

To assess privacy risk, we performed distance-, local density-, and classifier-based membership inference attacks (MIAs)[18], as well as classifier-based attribute inference attacks (AIAs)[19]. Membership inference attacks aim to determine whether a given data point was part of the training data used to fit the synthesizer. Attribute inference attacks attempt to infer the value of a sensitive target variable given a set of key variables, which are typically assumed to be publicly available or easily accessible. The selection of key and target variables for each dataset is provided in Table S25.

Publication	Key variables	Target variable
Schikowski et al.[5]	Age, Sex	Hypertension
Fischer et al.[3]	Age, Sex, Height, Weight, Study Center	VAT
Wienbergen et al.[6]	Age, Sex, BMI, Education, Birth Germany	EOMI
Breau et al.[7]	Age, Sex, Weight, Height, Ethnicity	Income Household
Berger et al.[8]	Age, Sex, Study Center, Education	Suicidal Ideation
Tanoey et al.[9]	Age, Sex, Height, Migration Background	Diabetes

Table S25. Overview of key and target variables for each publication used for attribtute inference attacks. VAT, visceral adipose tissue; EOMI, early-onset myocardial infarction

The success of MIAs was evaluated using the true positive rate at a false positive rate of 0.01 (TPR@FPR=0.01). A value of 0.01 corresponds to performance equivalent to random guessing, while higher values indicate an increased privacy risk. In practice, the realistic privacy risk depends on the prevalence of training records in the underlying population and can therefore be substantially lower when the training dataset represents only a small fraction of that population. Furthermore, these attacks assume that an adversary has access to a reference dataset from the population. In our evaluation, this assumption was simulated using train–test splits, but such information may not always be available in practice. At the same time, the considered attacks represent only a subset of possible inference strategies. More sophisticated and computationally intensive approaches, such as shadow modelling attacks[19], could potentially achieve stronger results. However, unlike differentially private[14] methods, empirical attacks can never provide formal guarantees, as stronger attacks may exist or emerge in the future.

AIAs success was assessed via the area under the receiver operating characteristic curve (AUROC) for categorical targets and MASD for continuous targets (Fischer et al.[3]), using a random forest model. Results were compared to a baseline model trained on real test data. Perfect privacy preservation is indicated when a model trained on synthetic data performs no better than the baseline trained on test data.

The results are presented in Supplement Figures S15–S17. The ARF models consistently ranked among the synthesizers with the highest utility. Lower values of the minimum node size generally achieved higher utility, with only minor differences across minimum node sizes of 2, 5, and 10. We note that the results for ARF with a minimum node size of 2 were lower than those reported in the main text, as expected given the reduced training sample size in this evaluation. While utility fluctuated across most of the alternative synthesizers, synthpop was the most consistent competitor, showing better utility values than the ARF models for Wienbergen et al.[6] (synthpop: CIO $=$ 0.776; ARF: CIO $\leq$ 0.698) and Berger et al.[8] (synthpop: CIO $=$ 0.469; ARF: CIO $\leq$ 0.343), lower utility for Tanoey et al.[9] (synthpop: CIO $=$ 0.557; ARF: CIO $\leq$ 0.679), and comparable performance on the remaining datasets.

However, ARF consistently exhibited lower MIA risks than synthpop, which showed increased risks particularly for Wienbergen et al. (synthpop: TPR@FPR=0.01 $=$ 0.100; ARF: TPR@FPR=0.01 $\leq$ 0.0388) and Schikowski et al.[5] (synthpop: TPR@FPR=0.01 $=$ 0.053; ARF: TPR@FPR=0.01 $\leq$ 0.019). An exception was Berger et al.[8], where all models exhibited comparably low utility (CIO $\leq$ 0.5) and synthpop demonstrated the overall best utility–privacy trade-off. The elevated MIA risk for ARF with a minimum node size of 2 in Berger et al.[8] can be attributed to the dataset consisting almost entirely of categorical variables, where the limited combination space leads to overly close reproduction of feature combinations. Substantially lower risk values were achieved with minimum node sizes greater than 2. For Breau et al.[7], MIA risk values were elevated for all synthesizers, presumably due to the small dataset size, with TVAE achieving the most favourable utility–privacy trade-off.

While ARF demonstrated negligible attribute inference attack (AIA) risks across experiments, synthpop substantially increased prediction performance for the target variables in Breau et al.[7] (synthpop: AUROC $=$ 0.688; test baseline: AUROC $=$ 0.5) and further increased the already high baseline AUROC for Wienbergen et al.[6] (synthpop: AUROC $=$ 0.864; test baseline: AUROC $=$ 0.822).

Overall, the ARF models demonstrated the most favourable balance between utility and privacy for Schikowski et al.[5], Fischer et al.[3], Wienbergen et al.[6] (together with the Bayesian Network), and Tanoey et al.[9] (together with PrivBayes). They showed the second-best trade-off behind TVAE for Breau et al.[7] and behind synthpop for Berger et al.[8].

Figure S15. Utility (MASD) for downstream descriptive analyses from Schikowski et al.[5] (Figure 1) and Breau et al.[7] (Figure 4) vs. privacy risk MIA/AIA

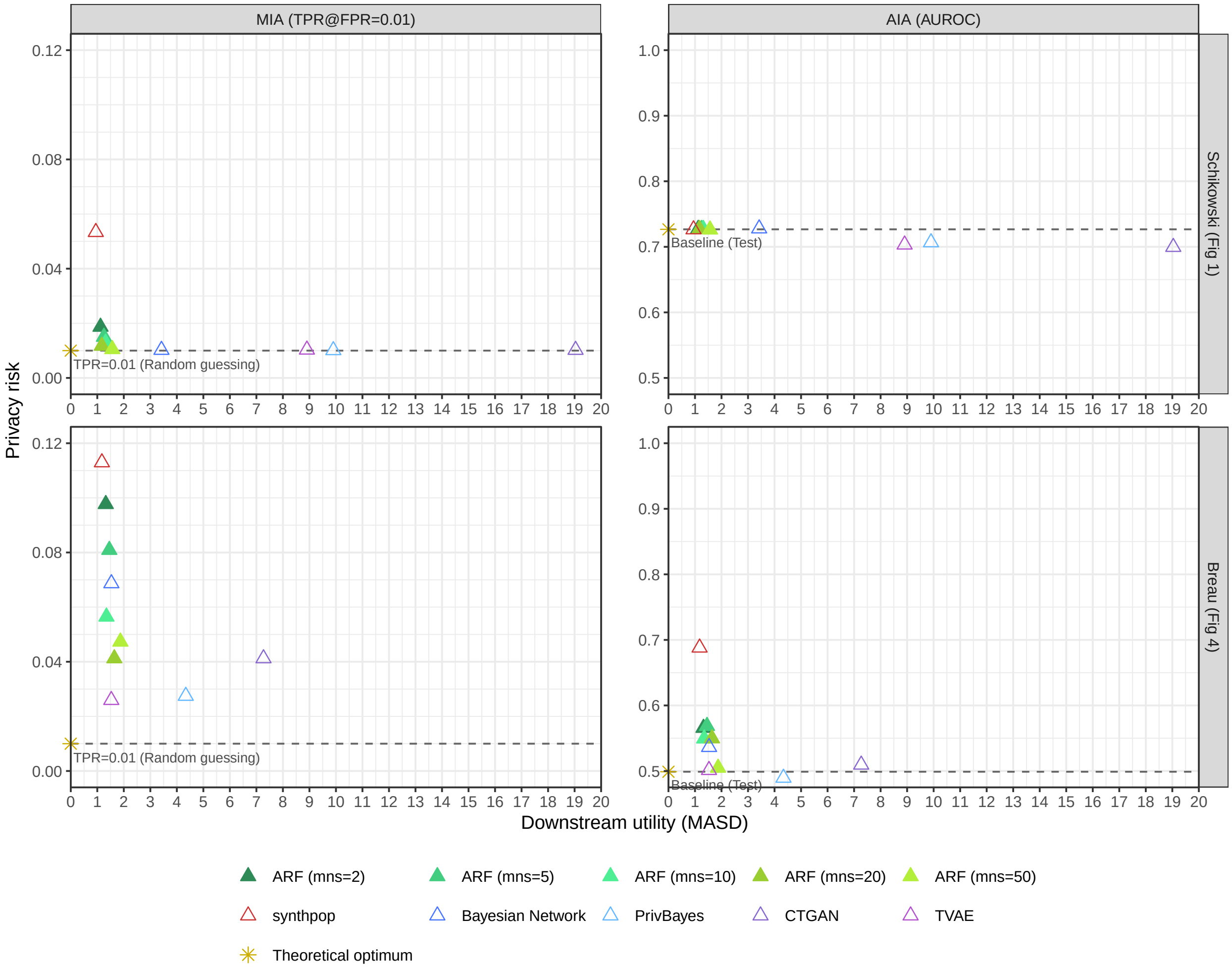

Comparison of ARF performance (downstream utility: MASD; privacy risk: MIA & AIA performance) across five minimum node sizes and five alternative synthesizers. Mean values over five synthesizer training repetitions are reported. ARF, adversarial random forests; MASD, mean absolute standardised difference; MIA, membership inference attack; TPR@FPR=0.01, attack true positive rate at a false positive rate of 0.01; AIA, attribute inference attack; AUROC, area under the receiver operating characteristic curve, Fig, figure; mns, minimum node size of random forest

Figure S16. Utility (WD) for downstream multivariate distribution analysis from Fischer et al.[3] (Figure 2) vs. privacy risk MIA/AIA

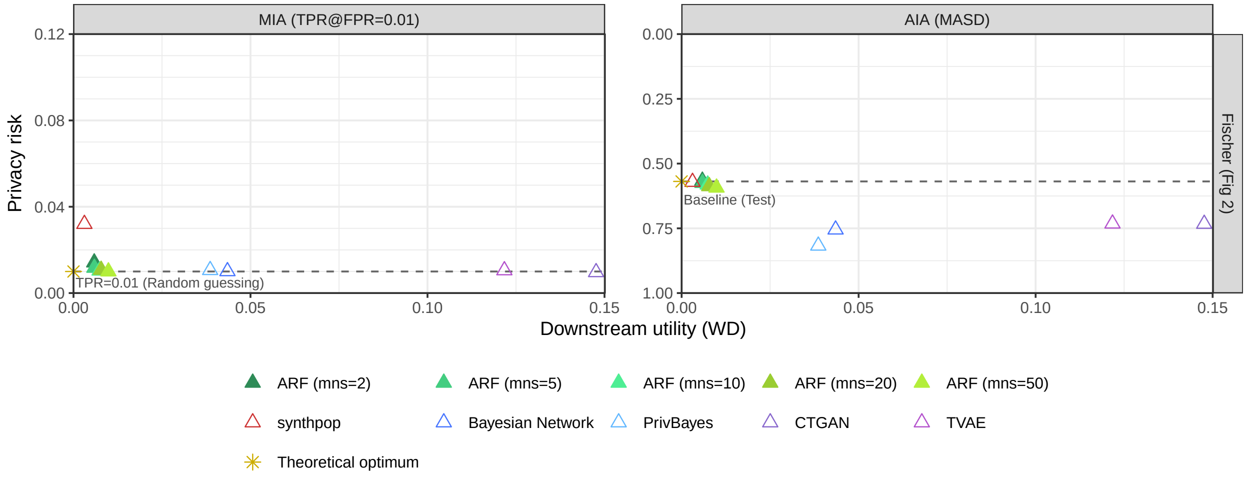

Comparison of ARF performance (downstream utility: WD; privacy risk: MIA & AIA performance) across five minimum node sizes and five alternative synthesizers. Mean values over five synthesizer training repetitions are reported. WD, multivariate Wasserstein distance; MASD, mean absolute standardised difference; MIA, membership inference attack; TPR@FPR=0.01, attack true positive rate at a false positive rate of 0.01; AIA, attribute inference attack; AUROC, area under the receiver operating characteristic curve, Fig, figure; mns, minimum node size of random forest

Figure S17. Utility (CIO) for downstream regression analyses from Wienbergen et al.[6] (Figure 3), Berger et al.[8] (Figure 5) and Tanoey et al.[9] (Figure 6) vs. privacy risk MIA/AIA

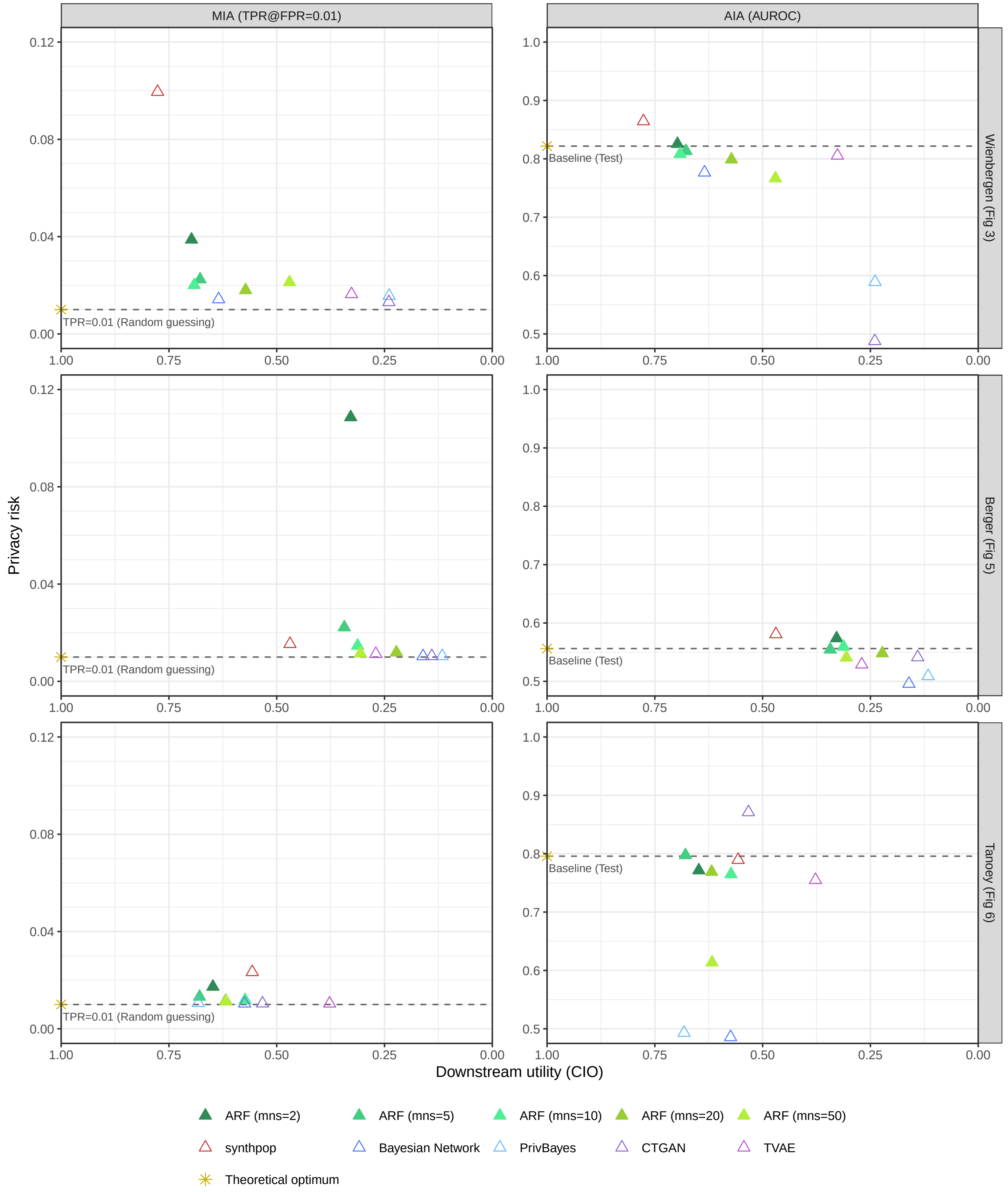

Comparison of ARF performance (downstream utility: CIO; privacy risk: MIA & AIA performance) across five minimum node sizes and five alternative synthesizers. Mean values over five synthesizer training repetitions are reported. CIO, mean confidence interval overlap; MIA, membership inference attack; TPR@FPR=0.01, attack true positive rate at a false positive rate of 0.01; AIA, attribute inference attack; AUROC, area under the receiver operating characteristic curve, Fig, figure; mns, minimum node size of random forest

C.2 Generalisation to unseen test data

For many applications, such as the use of synthetic data as test data or for data augmentation, it is important that the learned distributions generalise to the underlying population or unseen data rather than merely reproducing the training sample distribution. To assess this, we measured the multivariate Wasserstein distance (WD)[4] between synthetic data and test data not used for synthesizer training. Gower's distance[20] was used as the ground metric, enabling the application of WD to mixed-type data.

The results are shown in Supplement Figure S18 and plotted against the MIA privacy risks previously presented in Supplement A. For most datasets, the findings were consistent with the downstream utility results reported in Supplement C.1. Some differences were observed: TVAE now showed the highest distributional similarity to the test data for Berger et al.[8], while synthpop achieved the highest distributional similarity for Tanoey et al.[9], albeit at the cost of the highest MIA risk.

Overall, the results indicate that ARF did not merely reproduce the training distribution but achieves competitive generalisation to unseen data while often maintaining a favourable privacy risk level in comparison to the alternative synthesizers.

Figure S18. Distributional similarity (WD Syn-Test) vs. privacy risk MIA

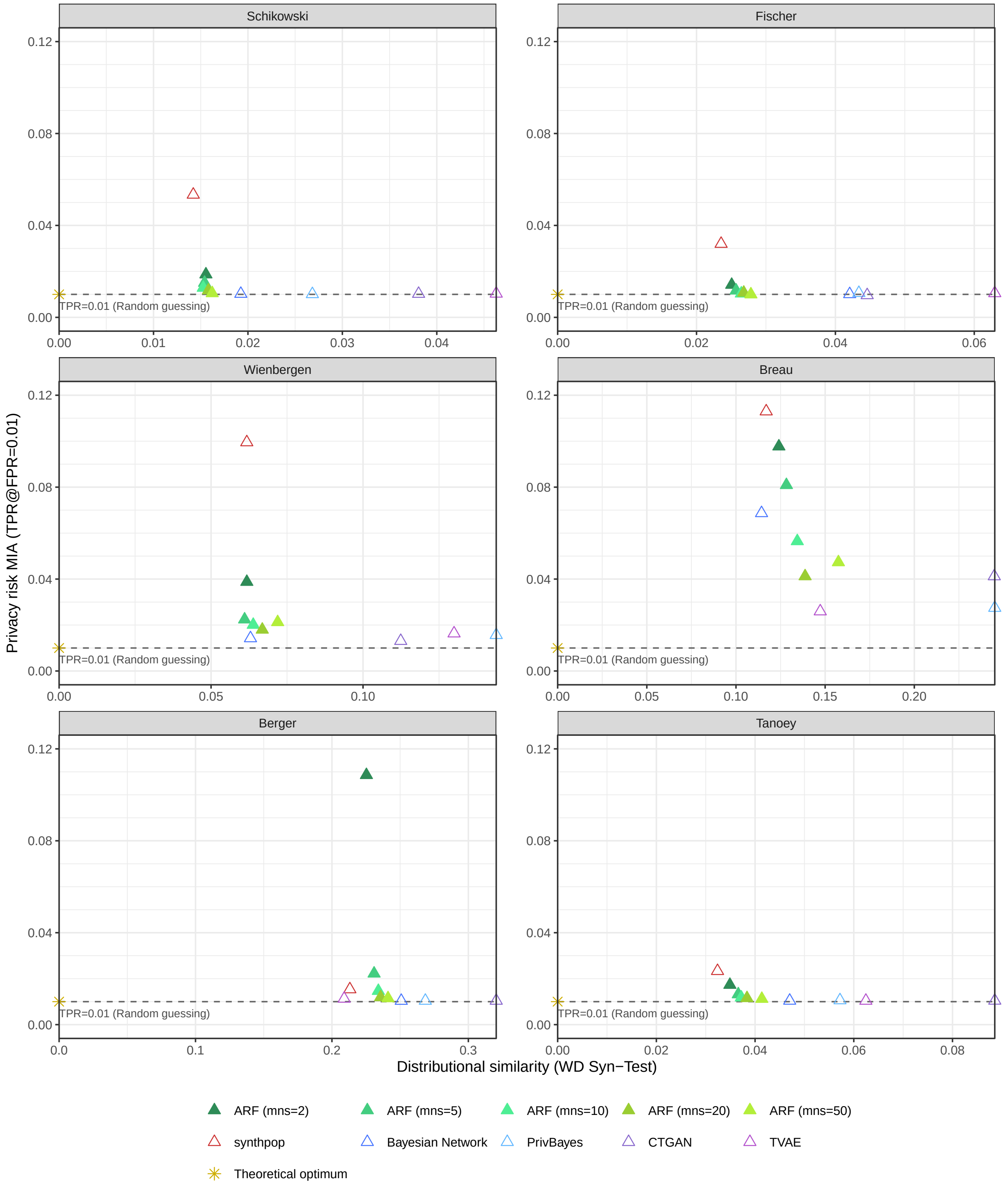

Comparison of ARF performance (distributional similarity: WD Syn-Test; privacy risk: MIA performance) across five minimum node sizes and five alternative synthesizers. Mean values over five synthesizer training repetitions are reported. WD Syn-Test, multivariate Wasserstein distance between synthetic and test data not used for synthesizer training; MIA, membership inference attack; TPR@FPR=0.01, attack true positive rate at a false positive rate of 0.01; mns, minimum node size of random forest

C.3 Runtime

Supplement Figure S19 shows runtime results for the smallest dataset (Breau et al.[7]) and the largest dataset (Berger et al.[8]). For Breau et al.[7], ARF and synthpop finished training and sampling in under 0.5 seconds, while the deep learning–based approaches required more than ten seconds and the Bayesian network–based approaches more than two minutes. For Berger et al.[8], ARF (median runtime: 35.29 seconds) performed orders of magnitude faster than all other synthesizers.

Figure S19. Synthesis runtime for Breau et al.[7] and Berger et al.[8]

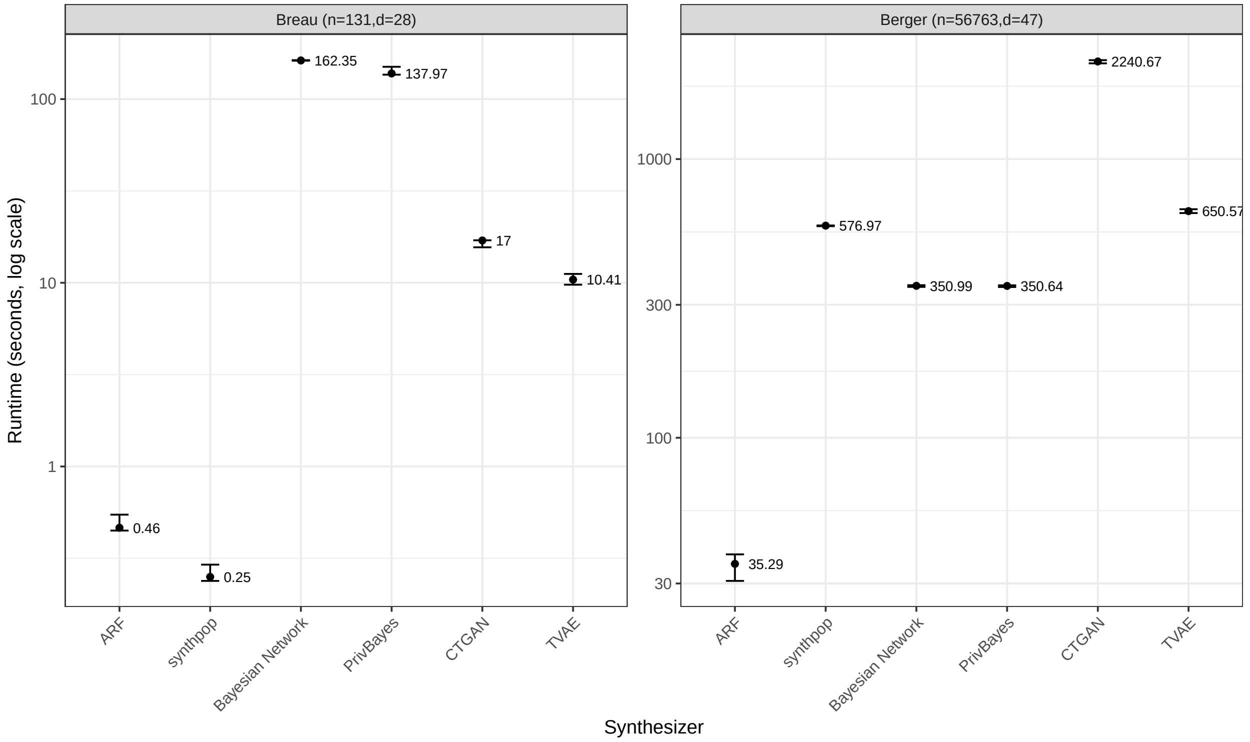

Synthesis runtime comparison (synthesizer training and sampling in seconds) for ARF and five alternative synthesizers. Median, minimum, and maximum values over five synthesizer repetitions are reported. ARF, adversarial random forests with minumum node size of two (default); n, number of training samples; d, dimensionality

References

[1] Bradley Efron and Robert J Tibshirani. *An introduction to the bootstrap*. New York: Chapman and Hall/CRC, 1994.

[2] Jörg Drechsler. Challenges in measuring utility for fully synthetic data. *Priv Stat Databases*, LNCS 2022;**13463**:220–33.

[3] Beate Fischer, Anja M Sedlmeier, Saskia Hartwig, et al. Anthropometrische Messungen in der NAKO Gesundheitsstudie—mehr als nur Größe und Gewicht [Anthropometric measures in the German National Cohort—more than weight and height]. *Bundesgesundheitsblatt, Gesundheitsforschung, Gesundheitsschutz* 2020;**63**(3):290–300.

[4] Cédric Villani. *Topics in optimal transportation*: American Mathematical Soc., 2021.

[5] Tamara Schikowski, Claudia Wigmann, Kateryna B Fuks, et al. Blutdruckmessung in der NAKO—methodische Unterschiede, Blutdruckverteilung und Bekanntheit der Hypertonie im Vergleich zu anderen bevölkerungsbezogenen Studien in Deutschland [Blood pressure measurement in the NAKO German National Cohort (GNC)—differences in methods, distribution of blood pressure values, and awareness of hypertension compared to other population-based studies in Germany]. *Bundesgesundheitsblatt Gesundheitsforschung Gesundheitsschutz* 2020;**63**(4):452–64.

[6] Harm Wienbergen, Daniel Boakye, Kathrin Günther, et al. Lifestyle and metabolic risk factors in patients with early-onset myocardial infarction: a case-control study. *Eur J Prev Cardiol* 2022;**29**(16):2076–87.

[7] Becky Breau, Hannah J Coyle-Asbil, Jess Haines, David WL Ma, and Lori Ann Vallis. ActiGraph cutpoints impact physical activity and sedentary behavior outcomes in young children. *J Meas Phys Behav* 2022;**5**(2):85–96.

[8] Klaus Berger, Steffi Riedel-Heller, Alexander Pabst, Marcella Rietschel, Dirk Richter, and NAKO-Konsortium. Einsamkeit während der ersten Welle der SARS-CoV-2-Pandemie—Ergebnisse der NAKO-Gesundheitsstudie [Loneliness during the first wave of the SARS-CoV-2 pandemic—results of the German National Cohort (NAKO)]. *Bundesgesundheitsblatt, Gesundheitsforschung, Gesundheitsschutz* 2021;**64**(9):1157–64.

[9] Justine Tanoey, Christina Baechle, Hermann Brenner, et al. Birth order, caesarean section, or daycare attendance in relation to child-and adult-onset type 1 diabetes: results from the German National Cohort. *Int J Environ Res Public Health* 2022;**19**(17):e10880.

[10] Philipp Probst, Marvin N Wright, and Anne-Laure Boulesteix. Hyperparameters and tuning strategies for random forest. *WIREs Data Min Knowl Discov* 2019;**9**(3):e1301.

[11] G Charbel N Kindji, Lina M Rojas-Barahona, Elisa Fromont, and Tanguy Urvoy. Tabular data generation models: An in-depth survey and performance benchmarks with extensive tuning. *Neurocomputing* 2025:131655.

[12] Beata Nowok, Gillian M. Raab, and Chris Dibben. synthpop: bespoke creation of synthetic data in R. *J Stat Softw* 2016;**74**(11):1–26.

[13] Daphne Koller and Nir Friedman. *Probabilistic graphical models: principles and techniques*: MIT press, 2009.

[14] Cynthia Dwork and Aaron Roth. The algorithmic foundations of differential privacy. *Found Trends Theor Comput Sci* 2014;**9**(3-4):211–487.

[15] Jun Zhang, Graham Cormode, Cecilia M. Procopiuc, Divesh Srivastava, and Xiaokui Xiao. PrivBayes: private data release via Bayesian networks. *ACM Trans Database Syst* 2017;**42**(4).

[16] Lei Xu, Maria Skoularidou, Alfredo Cuesta-Infante, and Kalyan Veeramachaneni. Modeling tabular data using conditional GAN. *Adv Neural Inf Process Syst* 2019;**32**.

[17] Hengrui Zhang, Jiani Zhang, Zhengyuan Shen, et al. Mixed-type tabular data synthesis with score-based diffusion in latent space. *ICLR* 2024.

[18] Joshua Ward, Yuxuan Yang, Chi-Hua Wang, and Guang Cheng. Ensembling membership inference attacks against tabular generative models. *AISec '25*, 2026:182–93.

[19] Theresa Stadler, Bristena Oprisanu, and Carmela Troncoso. Synthetic data – annonymisation groundhog day. *Proc USENIX Secur Symp* 2022:1451–68.

[20] John C Gower. A general coefficient of similarity and some of its properties. *Biometrics* 1971:857–71.
