# Supplementary material for: Can synthetic data reproduce real-world findings in epidemiology? A replication study using adversarial random forests
Source: Int J Epidemiol. 2026 Aug 29;55(5):dyag164. doi: 10.1093/ije/dyag164 (PMC13525842; doi:10.1093/ije/dyag164)
Supplement: Supplementary file 1 [file dyag164_supplementary_data.zip › ARF_SynthEpiRep_code.zip!/ARF_SynthEpiRep-main/GFHS/Breau et al./plots/Breau_fig1.pdf]

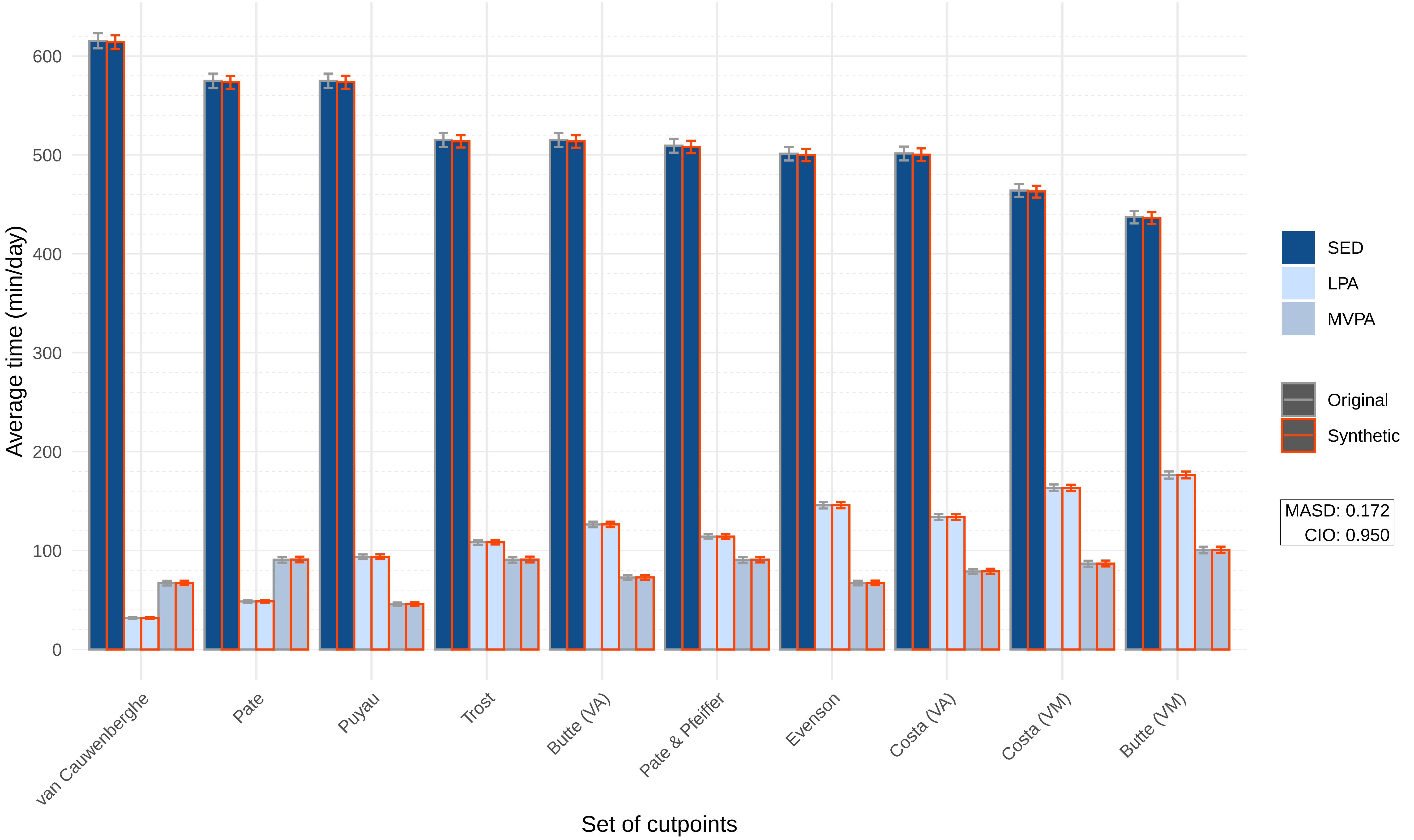

Average time (min/day)
0
100
200
300
400
500
600
van Cauwenberghe
Pate
Puyau
Trost
Butte (VA)
Pate & Pfeiffer
Evenson
Costa (VA)
Costa (VM)
Butte (VM)
Set of cutpoints
SED
LPA
MVPA
Original
Synthetic
MASD: 0.172
CIO: 0.950
