# Supplementary material for: Can synthetic data reproduce real-world findings in epidemiology? A replication study using adversarial random forests
Source: Int J Epidemiol. 2026 Aug 29;55(5):dyag164. doi: 10.1093/ije/dyag164 (PMC13525842; doi:10.1093/ije/dyag164)
Supplement: Supplementary file 1 [file dyag164_supplementary_data.zip › ARF_SynthEpiRep_code.zip!/ARF_SynthEpiRep-main/NAKO/Berger et al./plots/Berger_fig1.pdf]

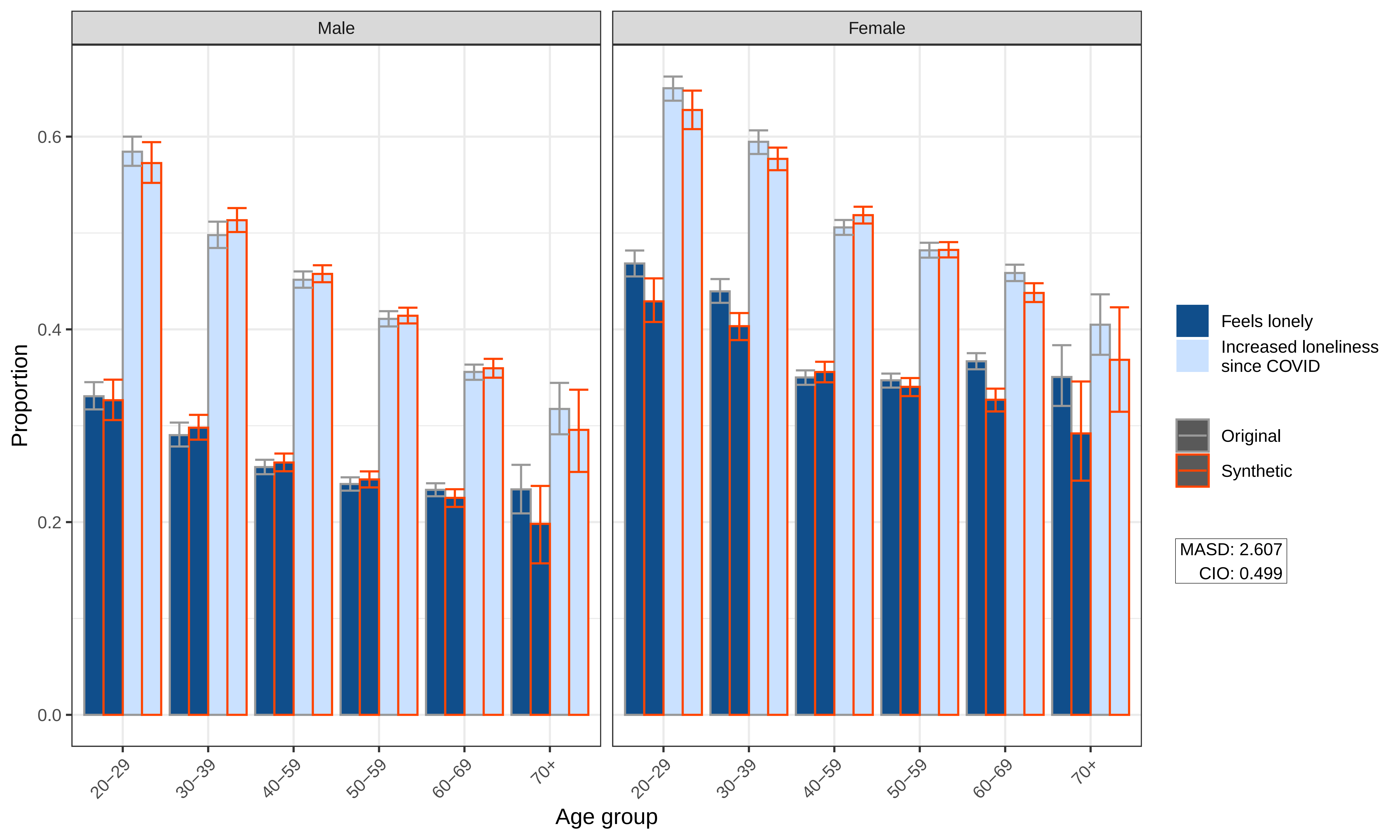

Male
Female
0.6
0.4
0.2
0.0
Proportion
20–29
30–39
40–59
50–59
60–69
70+
Age group
Feels lonely
Increased loneliness since COVID
Original
Synthetic
MASD: 2.607
CIO: 0.499
