# Supplementary material for: Can synthetic data reproduce real-world findings in epidemiology? A replication study using adversarial random forests
Source: Int J Epidemiol. 2026 Aug 29;55(5):dyag164. doi: 10.1093/ije/dyag164 (PMC13525842; doi:10.1093/ije/dyag164)
Supplement: Supplementary file 1 [file dyag164_supplementary_data.zip › ARF_SynthEpiRep_code.zip!/ARF_SynthEpiRep-main/NAKO/Berger et al./plots/Berger_fig2.pdf]

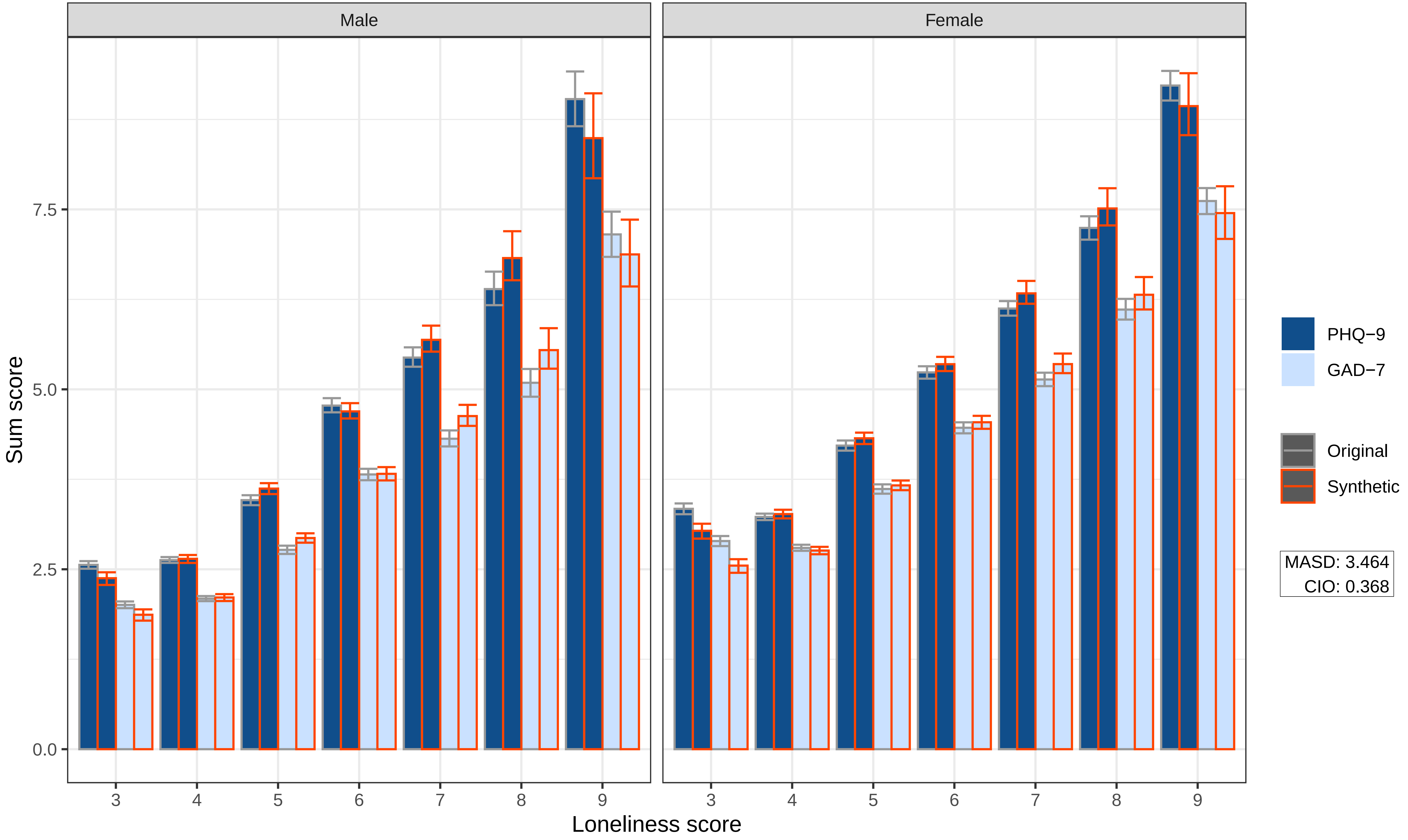

Male
Female
Sum score
7.5
5.0
2.5
0.0
3
4
5
6
7
8
9
Loneliness score
PHQ−9
GAD−7
Original
Synthetic
MASD: 3.464
CIO: 0.368
