# Supplementary material for: Can synthetic data reproduce real-world findings in epidemiology? A replication study using adversarial random forests
Source: Int J Epidemiol. 2026 Aug 29;55(5):dyag164. doi: 10.1093/ije/dyag164 (PMC13525842; doi:10.1093/ije/dyag164)
Supplement: Supplementary file 1 [file dyag164_supplementary_data.zip › ARF_SynthEpiRep_code.zip!/ARF_SynthEpiRep-main/NAKO/Berger et al./plots/Berger_fig3.pdf]

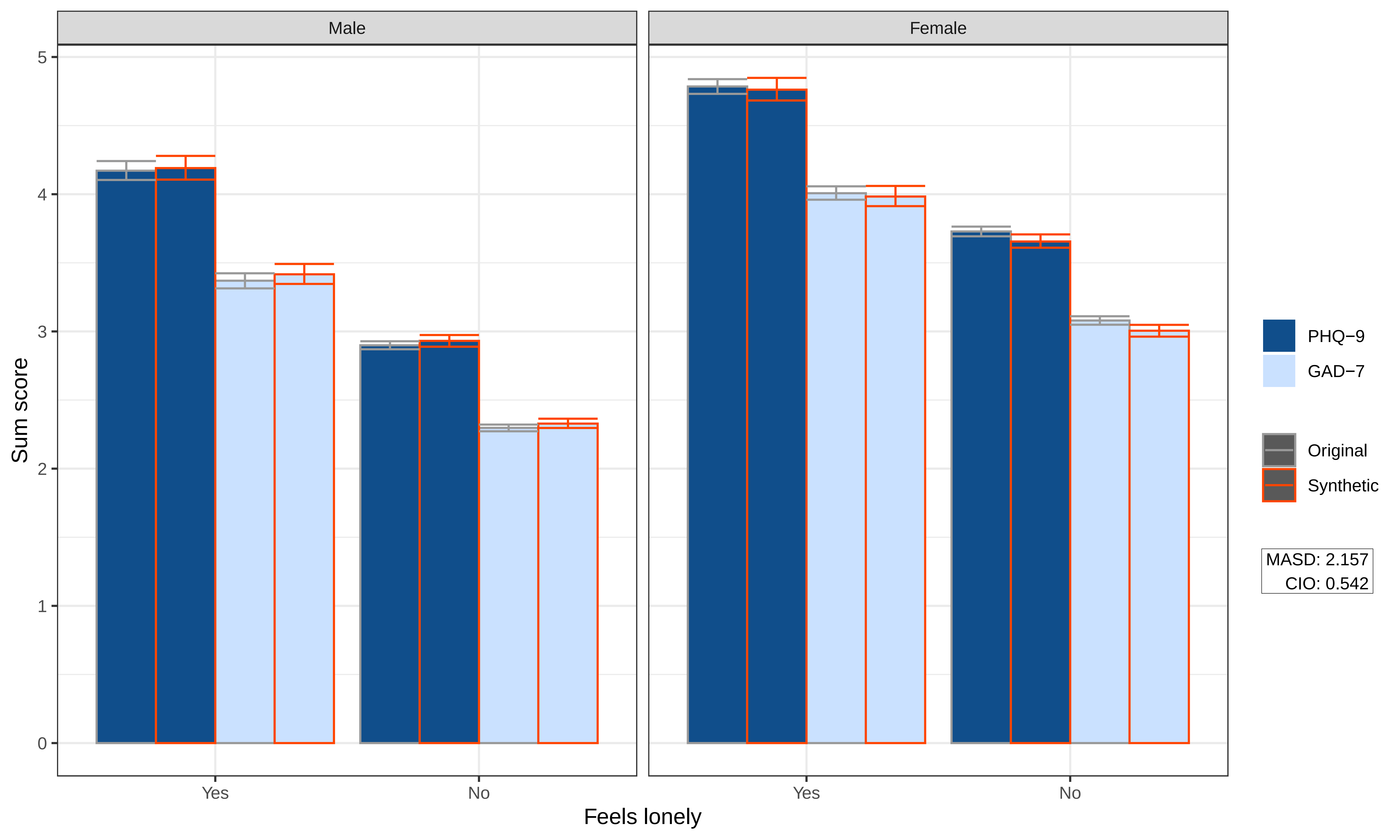
Male
Female
Sum score
5
4
3
2
1
0
Yes
No
Yes
No
Feels lonely
PHQ−9
GAD−7
Original
Synthetic
MASD: 2.157
CIO: 0.542
