# Supplementary material for: Can synthetic data reproduce real-world findings in epidemiology? A replication study using adversarial random forests
Source: Int J Epidemiol. 2026 Aug 29;55(5):dyag164. doi: 10.1093/ije/dyag164 (PMC13525842; doi:10.1093/ije/dyag164)
Supplement: Supplementary file 1 [file dyag164_supplementary_data.zip › ARF_SynthEpiRep_code.zip!/ARF_SynthEpiRep-main/NAKO/Fischer et al./plots/Fischer_fig3.pdf]

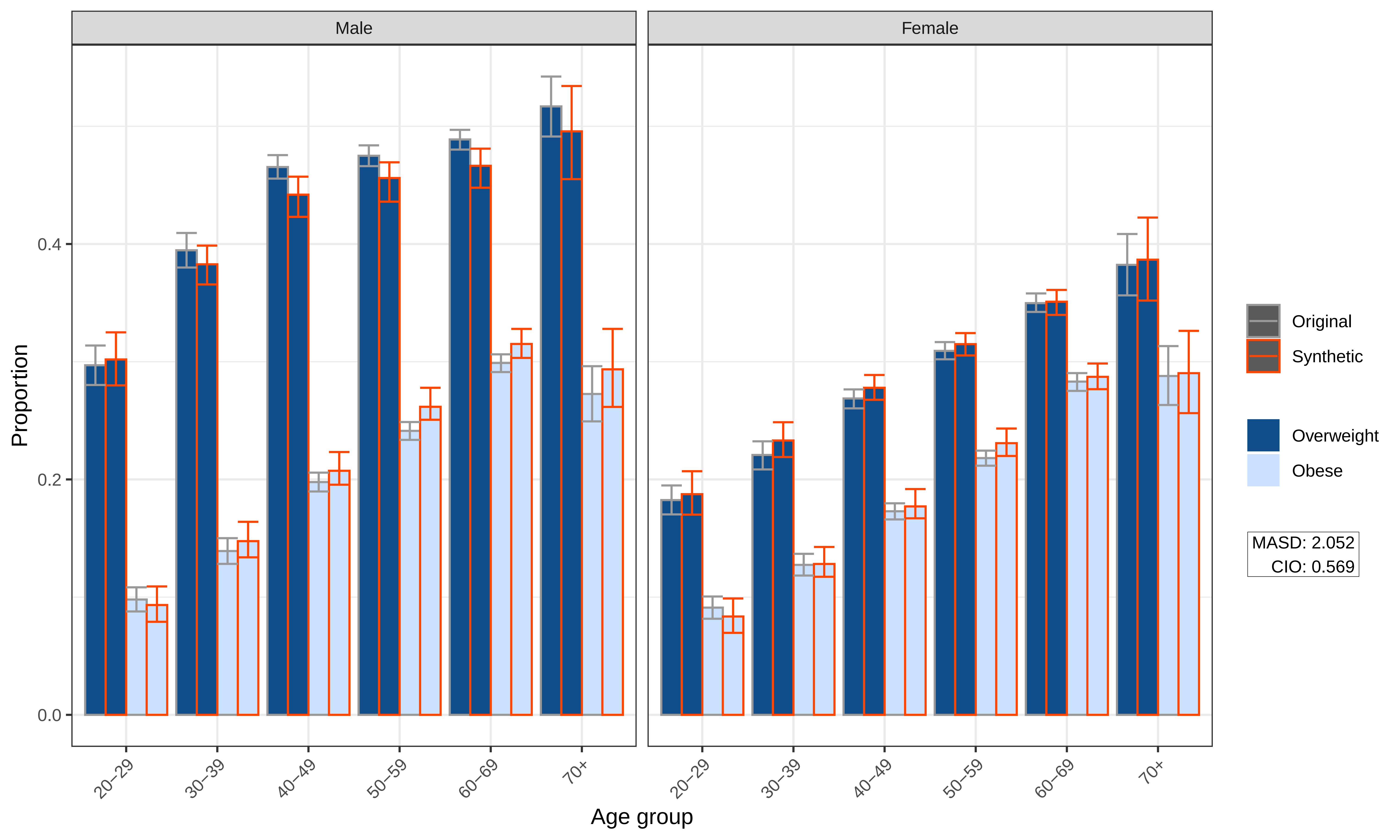

Male
Female
Proportion
0.0
0.2
0.4
20–29
30–39
40–49
50–59
60–69
70+
Age group
Original
Synthetic
Overweight
Obese
MASD: 2.052
CIO: 0.569
