# Supplementary material for: Can synthetic data reproduce real-world findings in epidemiology? A replication study using adversarial random forests
Source: Int J Epidemiol. 2026 Aug 29;55(5):dyag164. doi: 10.1093/ije/dyag164 (PMC13525842; doi:10.1093/ije/dyag164)
Supplement: Supplementary file 1 [file dyag164_supplementary_data.zip › ARF_SynthEpiRep_code.zip!/ARF_SynthEpiRep-main/NAKO/Fischer et al./plots/Fischer_fig5.pdf]

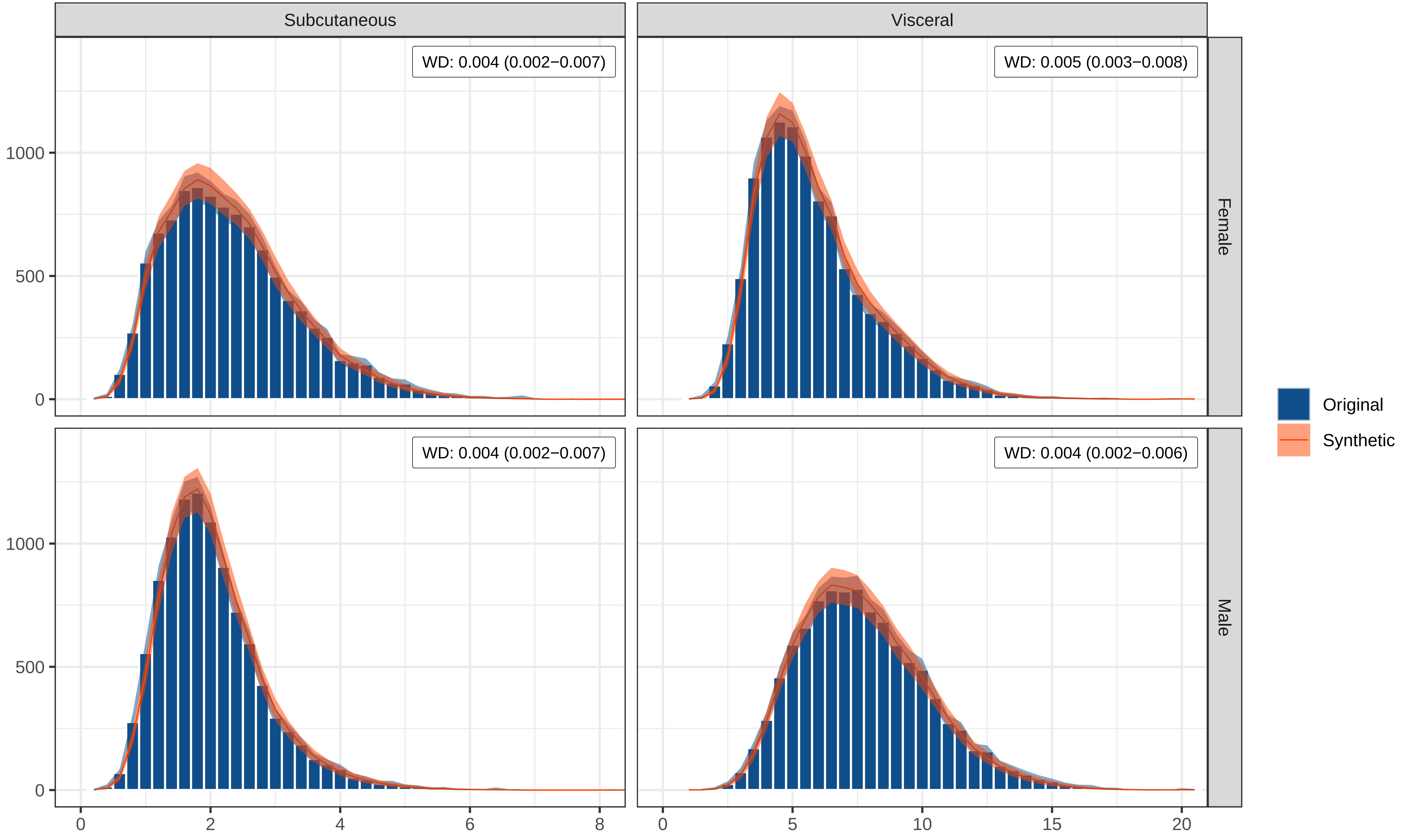

Subcutaneous
Visceral
Female
Male
WD: 0.004 (0.002−0.007)
WD: 0.005 (0.003−0.008)
WD: 0.004 (0.002−0.007)
WD: 0.004 (0.002−0.006)
1000
500
0
0
2
4
6
8
0
5
10
15
20
Original
Synthetic
