# Supplementary material for: Can synthetic data reproduce real-world findings in epidemiology? A replication study using adversarial random forests
Source: Int J Epidemiol. 2026 Aug 29;55(5):dyag164. doi: 10.1093/ije/dyag164 (PMC13525842; doi:10.1093/ije/dyag164)
Supplement: Supplementary file 1 [file dyag164_supplementary_data.zip › ARF_SynthEpiRep_code.zip!/ARF_SynthEpiRep-main/NAKO/Schikowski et al./plots/Schikowski_fig2.pdf]

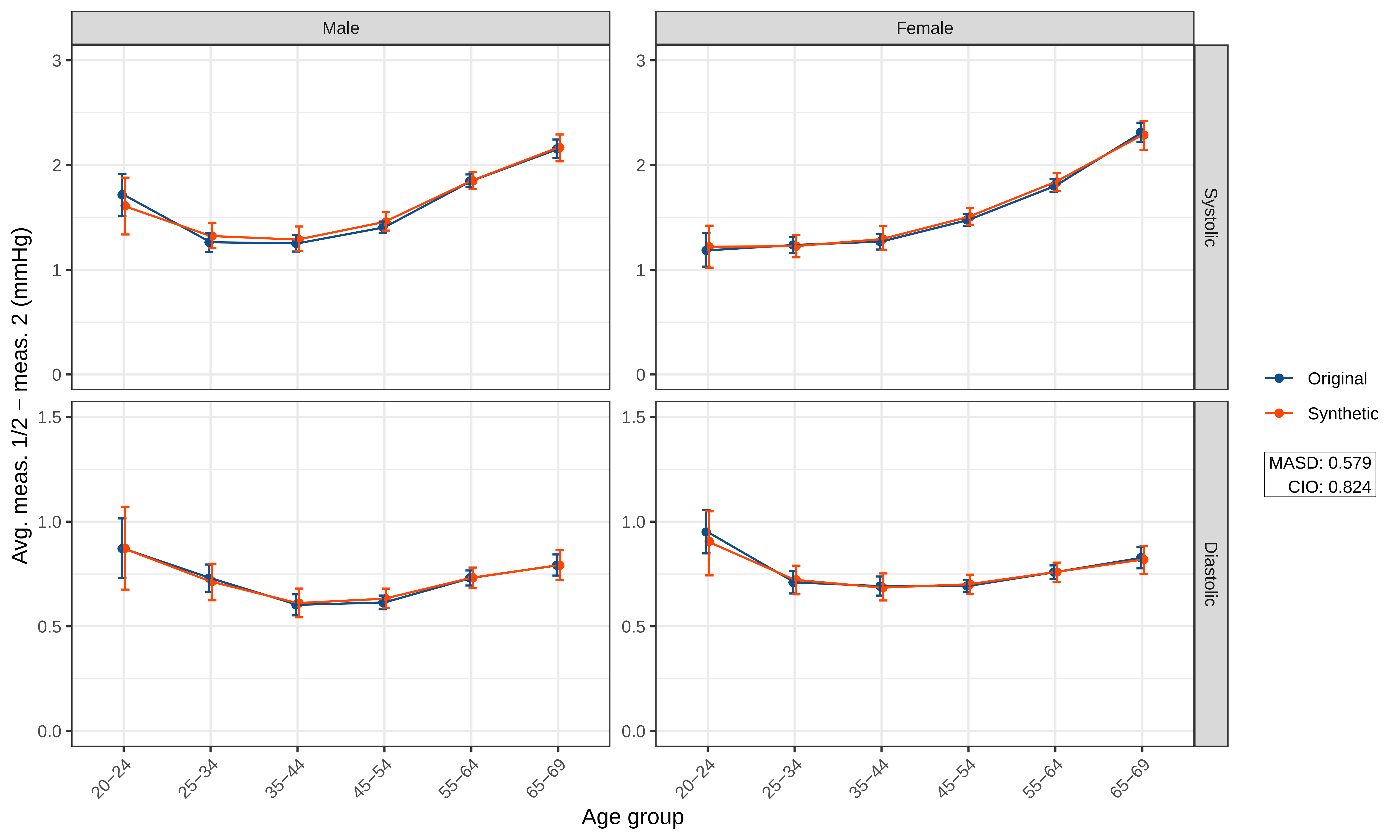
Male
Female
Systolic
Diastolic
Avg. meas. 1/2 − meas. 2 (mmHg)
Age group
20–24
25–34
35–44
45–54
55–64
65–69
0
1
2
3
0.0
0.5
1.0
1.5
Original
Synthetic
MASD: 0.579
CIO: 0.824
