# Supplementary material for: Can synthetic data reproduce real-world findings in epidemiology? A replication study using adversarial random forests
Source: Int J Epidemiol. 2026 Aug 29;55(5):dyag164. doi: 10.1093/ije/dyag164 (PMC13525842; doi:10.1093/ije/dyag164)
Supplement: Supplementary file 1 [file dyag164_supplementary_data.zip › ARF_SynthEpiRep_code.zip!/ARF_SynthEpiRep-main/NAKO/Schikowski et al./plots/Schikowski_fig3_4.pdf]

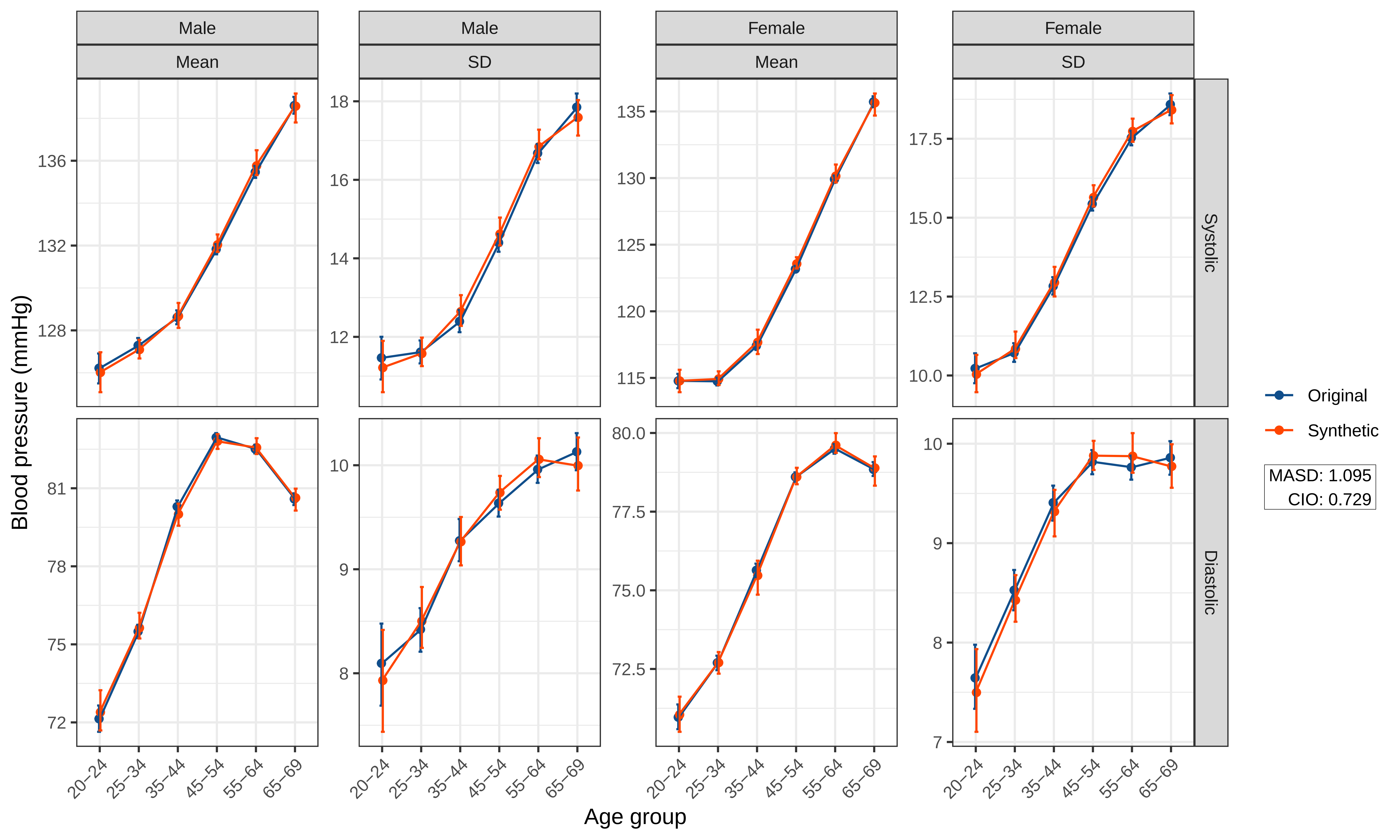
Male
Mean
Male
SD
Female
Mean
Female
SD
Systolic
Diastolic
Blood pressure (mmHg)
Age group
20–24
25–34
35–44
45–54
55–64
65–69
136
132
128
18
16
14
12
135
130
125
120
115
17.5
15.0
12.5
10.0
81
78
75
72
10
9
8
80.0
77.5
75.0
72.5
7
Original
Synthetic
MASD: 1.095
CIO: 0.729
