# Supplementary material for: Can synthetic data reproduce real-world findings in epidemiology? A replication study using adversarial random forests
Source: Int J Epidemiol. 2026 Aug 29;55(5):dyag164. doi: 10.1093/ije/dyag164 (PMC13525842; doi:10.1093/ije/dyag164)
Supplement: Supplementary file 1 [file dyag164_supplementary_data.zip › ARF_SynthEpiRep_code.zip!/ARF_SynthEpiRep-main/NAKO/Tanoey et al./plots/Tanoey_fig2_taskspec.pdf]

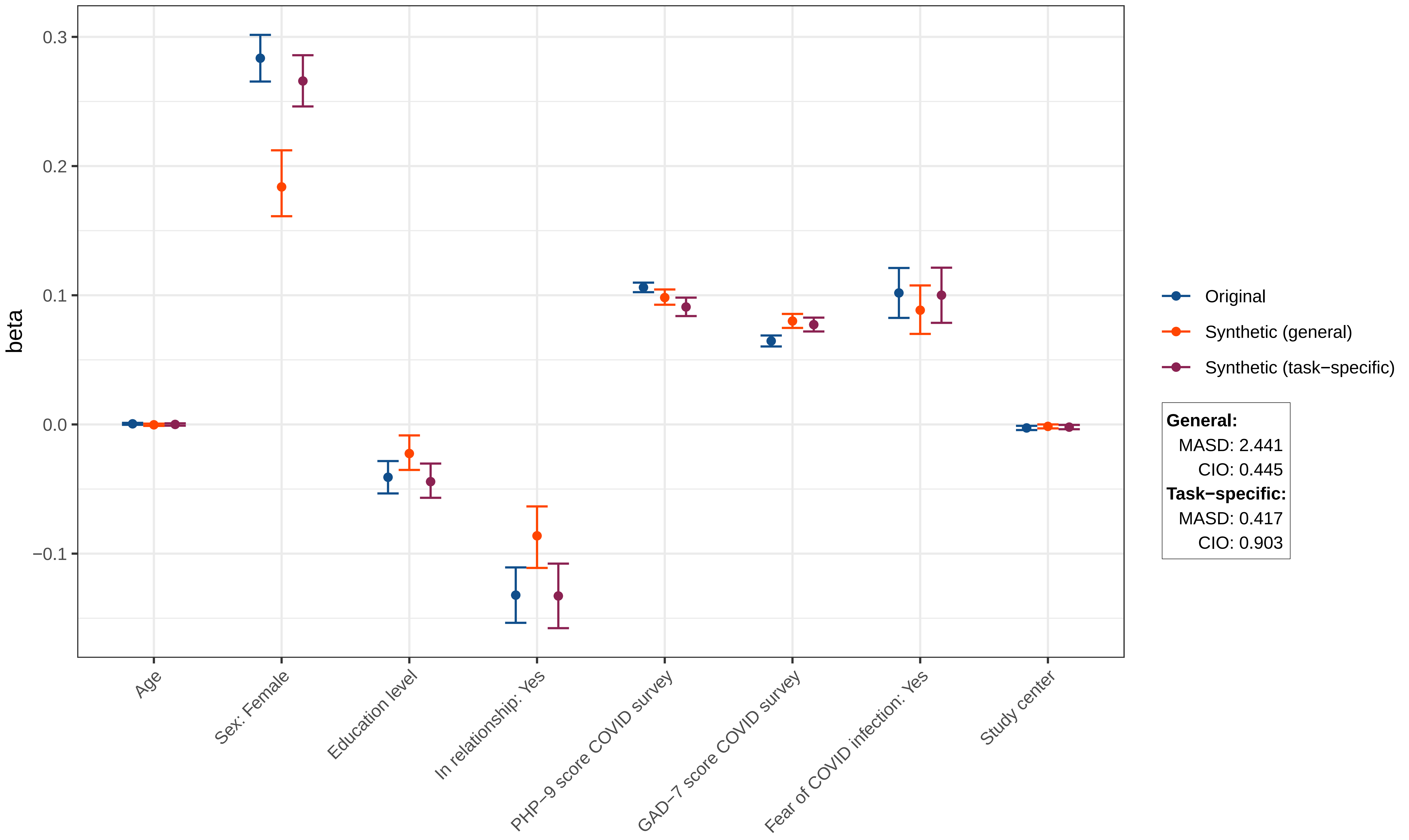

0.3
0.2
0.1
0.0
−0.1
beta
Age
Sex: Female
Education level
In relationship: Yes
PHP−9 score COVID survey
GAD−7 score COVID survey
Fear of COVID infection: Yes
Study center
Original
Synthetic (general)
Synthetic (task−specific)
General:
MASD: 2.441
CIO: 0.445
Task−specific:
MASD: 0.417
CIO: 0.903
