# Supplementary material for: Can synthetic data reproduce real-world findings in epidemiology? A replication study using adversarial random forests
Source: Int J Epidemiol. 2026 Aug 29;55(5):dyag164. doi: 10.1093/ije/dyag164 (PMC13525842; doi:10.1093/ije/dyag164)
Supplement: Supplementary file 1 [file dyag164_supplementary_data.zip › ARF_SynthEpiRep_code.zip!/ARF_SynthEpiRep-main/NAKO/Tanoey et al./plots/Tanoey_tab2_univariable_coxreg_taskspec.pdf]

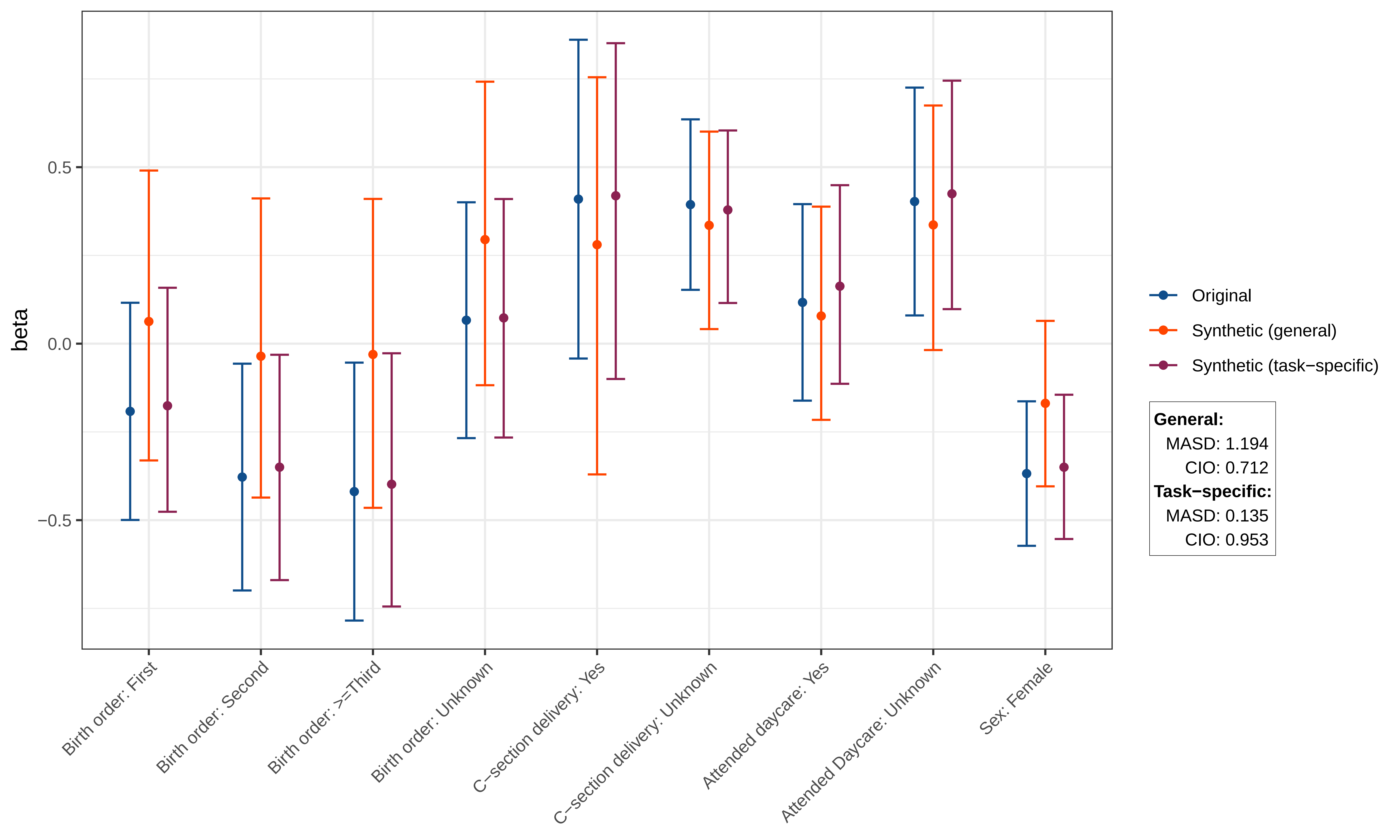

beta
0.5
0.0
−0.5
Birth order: First
Birth order: Second
Birth order: >=Third
Birth order: Unknown
C−section delivery: Yes
C−section delivery: Unknown
Attended daycare: Yes
Attended Daycare: Unknown
Sex: Female
Original
Synthetic (general)
Synthetic (task−specific)
General:
MASD: 1.194
CIO: 0.712
Task−specific:
MASD: 0.135
CIO: 0.953
