# Supplementary material for: Can synthetic data reproduce real-world findings in epidemiology? A replication study using adversarial random forests
Source: Int J Epidemiol. 2026 Aug 29;55(5):dyag164. doi: 10.1093/ije/dyag164 (PMC13525842; doi:10.1093/ije/dyag164)
Supplement: Supplementary file 1 [file dyag164_supplementary_data.zip › ARF_SynthEpiRep_code.zip!/ARF_SynthEpiRep-main/NAKO/Wienbergen et al./plots/Wienbergen_tab2_logreg_adjlvl1.pdf]

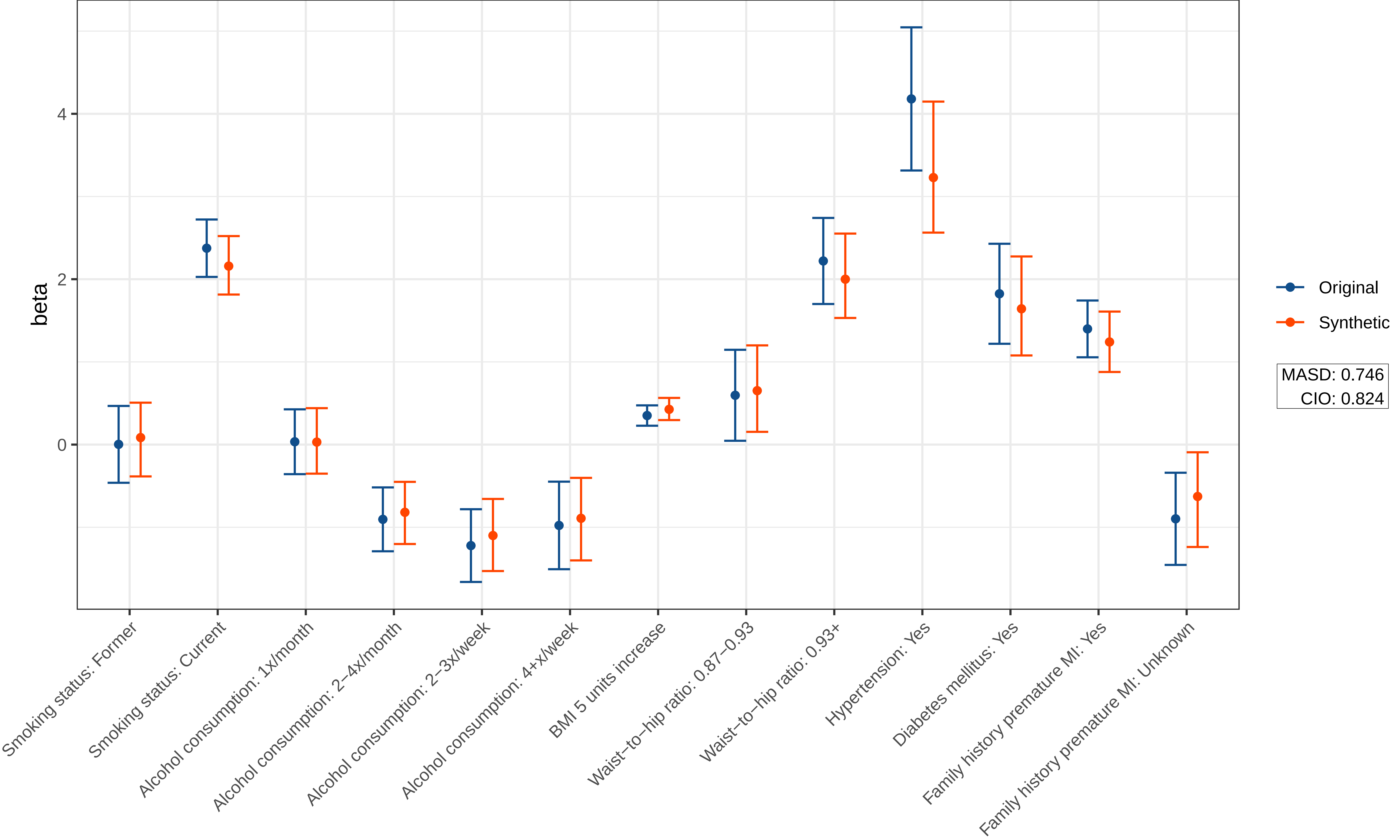

beta
4
2
0
Original
Synthetic
MASD: 0.746
CIO: 0.824
Smoking status: Former
Smoking status: Current
Alcohol consumption: 1x/month
Alcohol consumption: 2–4x/month
Alcohol consumption: 2–3x/week
Alcohol consumption: 4+x/week
BMI 5 units increase
Waist–to–hip ratio: 0.87–0.93
Waist–to–hip ratio: 0.93+
Hypertension: Yes
Diabetes mellitus: Yes
Family history premature MI: Yes
Family history premature MI: Unknown
