# Supplementary material for: Can synthetic data reproduce real-world findings in epidemiology? A replication study using adversarial random forests
Source: Int J Epidemiol. 2026 Aug 29;55(5):dyag164. doi: 10.1093/ije/dyag164 (PMC13525842; doi:10.1093/ije/dyag164)
Supplement: Supplementary file 1 [file dyag164_supplementary_data.zip › ARF_SynthEpiRep_code.zip!/ARF_SynthEpiRep-main/README.html]

README


# Can synthetic data reproduce real-world findings in epidemiology? A replication study using adversarial random forests

This repository contains the code which produced the results of the
manuscript “Can synthetic data reproduce real-world findings in
epidemiology? A replication study using adversarial random forests” by
Kapar et al. (2026).

## Summary

The paper proposes the use of adversarial
random forests (ARF) as a competitive, fast, and user-friendly
method for synthesizing tabular epidemiological data. To evaluate its
performance, statistical analyses from six epidemiological publications
were replicated and the results on synthetic data compared to original
findings. Additionally, the impact of dimensionality and variable
complexity on synthesis quality was assessed by limiting datasets to
variables relevant to individual analysis tasks, including necessary
derivations.

Supplementary analyses included a comparison of the ARF the effects
of the parameter `min_node_size` on synthesis quality, and a
comparison of the ARF to other popular synthesis methods for tabular
data. The trade-off between utility and privacy (measured by membership
and attribute inference attacks) was assessed as well as the
generalisation to unseen test data and the synthesis runtime.

## Replicated original publications

The replicated original publications cover blood pressure,
anthropometry, myocardial infarction, accelerometry, loneliness, and
diabetes, and are based on data from the German National Cohort (NAKO
Gesundheitsstudie), the Bremen STEMI Registry
U45 Study (BSR-U45), and the Guelph Family Health Study
(GFHS).

**Publication list**

- Tamara Schikowski, Claudia Wigmann, Kateryna B Fuks, et al.
  Blutdruckmessung in der NAKO—methodische Unterschiede,
  Blutdruckverteilung und Bekanntheit der Hypertonie im Vergleich zu
  anderen bevölkerungsbezogenen Studien in Deutschland [Blood pressure
  measurement in the NAKO German National Cohort (GNC)—differences in
  methods, distribution of blood pressure values, and awareness of
  hypertension compared to other population-based studies in Germany].
  *Bundesgesundheitsblatt Gesundheitsforschung Gesundheitsschutz*
  2020;**63**(4):452–64. https://doi.org/10.1007/s00103-020-03109-8
- Beate Fischer, Anja M Sedlmeier, Saskia Hartwig, et al.
  Anthropometrische Messungen in der NAKO Gesundheitsstudie—mehr als nur
  Größe und Gewicht [Anthropometric measures in the German National
  Cohort—more than weight and height]. *Bundesgesundheitsblatt,
  Gesundheitsforschung, Gesundheitsschutz*
  2020;**63**(3):290–300. https://doi.org/10.1007/s00103-020-03096-w
- Harm Wienbergen, Daniel Boakye, Kathrin Günther, et al. Lifestyle
  and metabolic risk factors in patients with early-onset myocardial
  infarction: a case-control study. *Eur J Prev Cardiol*
  2022;**29**(16):2076–87. https://doi.org/10.1093/eurjpc/zwac132
- Becky Breau, Hannah J Coyle-Asbil, Jess Haines, David WL Ma, and
  Lori Ann Vallis. ActiGraph cutpoints impact physical activity and
  sedentary behavior outcomes in young children. *J Meas Phys
  Behav* 2022;**5**(2):85–96. https://doi.org/10.1123/jmpb.2021-0042
- Klaus Berger, Steffi Riedel-Heller, Alexander Pabst, Marcella
  Rietschel, Dirk Richter, and NAKO-Konsortium. Einsamkeit während der
  ersten Welle der SARS-CoV-2-Pandemie—Ergebnisse der
  NAKO-Gesundheitsstudie [Loneliness during the first wave of the
  SARS-CoV-2 pandemic—results of the German National Cohort (NAKO)].
  *Bundesgesundheitsblatt, Gesundheitsforschung, Gesundheitsschutz*
  2021;**64**(9):1157–64. https://doi.org/10.1007/s00103-021-03393-y
- Justine Tanoey, Christina Baechle, Hermann Brenner, et al. Birth
  order, caesarean section, or daycare attendance in relation to child-and
  adult-onset type 1 diabetes: results from the German National Cohort.
  *Int J Environ Res Public Health*
  2022;**19**(17):e10880. https://doi.org/10.3390/ijerph191710880

## Data availablity

None of the data used in this work are openly available. Hence, this
repository does not contain any data files for reproduction.

The NAKO data are not openly available due to data protection
measures. However, scientists can apply for data access following the
official usage regulations and upon formal request to the NAKO use and
access committee (https://transfer.nako.de).

Participant data from the Bremen STEMI Registry U45 Study (BSR-U45)
are not publicly available.

Due to University of Guelph Research Ethics Board restrictions and
participant confidentiality, no GFHS participant data are publicly
available. The GFHS welcomes outside collaborators. Interested
investigators can contact GFHS investigators to explore this option,
which preserves participant confidentiality and meets the requirements
of the University of Guelph Research Ethics Board, to protect human
subjects.

## Repoistory organisation

- Publication-specific code is organised within subfolders of
  `/NAKO` and `/GFHS` folders
- Creation of full and task-specific original datasets:
  `create_train.R` (calls publication-specific code in
  publication folders)
- Data synthesis with R: `create_syn.R` (global for all
  publicaitons)
- Analyses replication and comparison: `run_analyses.R`
  (calls publication-specific code `analysis.R` and
  `analysis_taskspec.R` in publication folders)
- Results are stored in `/figures` and `/tables`
  subfolders of publication folders
- Supplementary analyses are stored in `/Supplement`

## Requirements

### Required packages

**For synthesis:**

- `arf`

**For statistical analyses:**

- `survival`
- `zscorer`
- `scales`

**For visualization:**

- `ggplot2`
- `ggh4x`
- `xtable`

**For parallel processing:**

- `doParallel`
- `foreach`

**For data processing and console output:**

- `data.table`

**For supplement:**

- `synthpop`
- `ddpm`(PyPI)
- `sdv` (PyPI)
- `ranger`
- `pROC`
- `caret`
- `approxOT`
- `philentropy`
- `binom`
- `microbenchmark`
