# Supplementary material for: Can synthetic data reproduce real-world findings in epidemiology? A replication study using adversarial random forests
Source: Int J Epidemiol. 2026 Aug 29;55(5):dyag164. doi: 10.1093/ije/dyag164 (PMC13525842; doi:10.1093/ije/dyag164)
Supplement: Supplementary file 1 [file dyag164_supplementary_data.zip › ARF_SynthEpiRep_code.zip!/ARF_SynthEpiRep-main/Supplement/plots/Appx_DU_CIO_Priv.pdf]

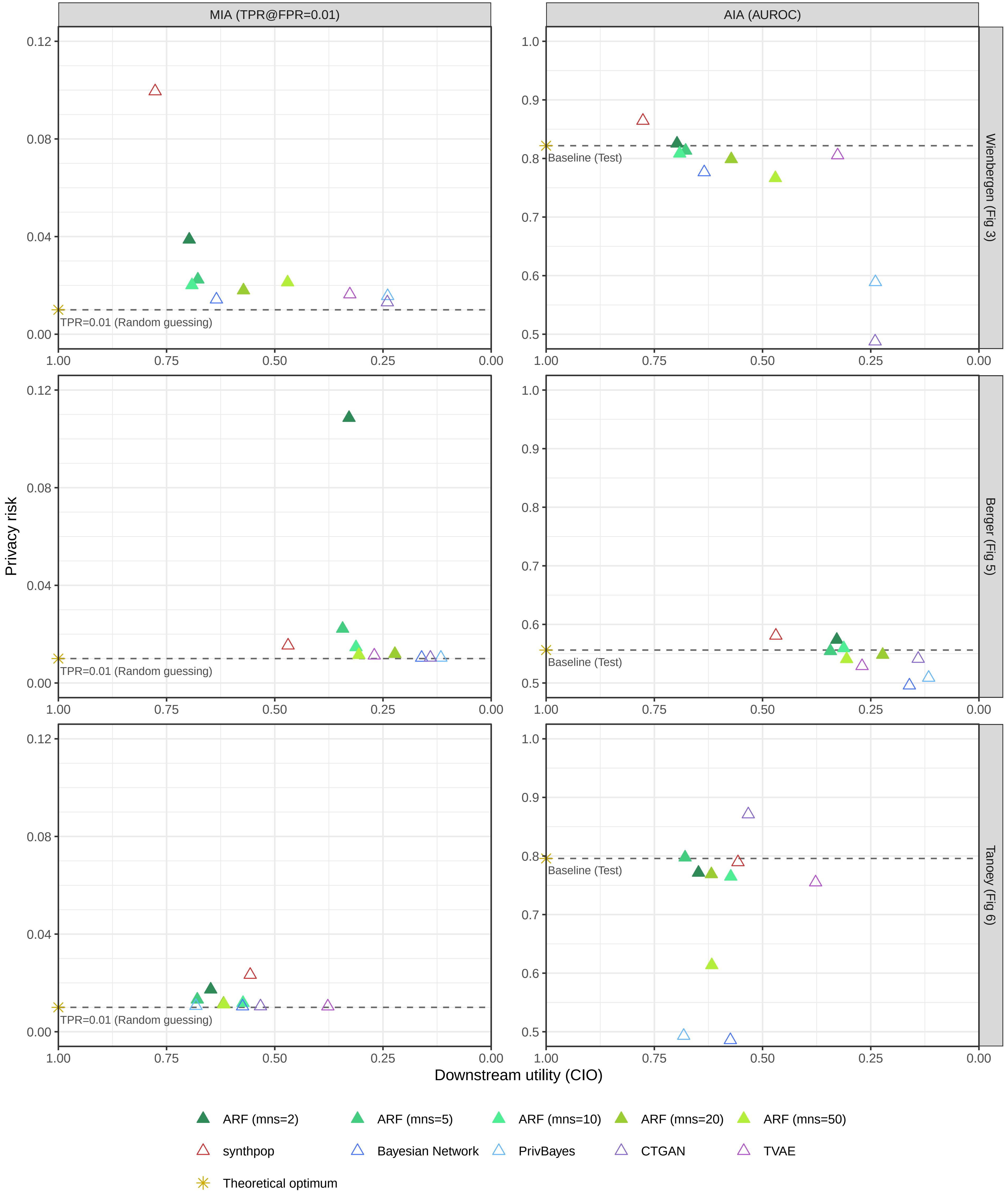

MIA (TPR@FPR=0.01)
AIA (AUROC)
Wienbergen (Fig 3)
Berger (Fig 5)
Tanoey (Fig 6)
TPR=0.01 (Random guessing)
Baseline (Test)
Privacy risk
Downstream utility (CIO)
ARF (mns=2)
ARF (mns=5)
ARF (mns=10)
ARF (mns=20)
ARF (mns=50)
synthpop
Bayesian Network
PrivBayes
CTGAN
TVAE
Theoretical optimum
