# Supplementary material for: Can synthetic data reproduce real-world findings in epidemiology? A replication study using adversarial random forests
Source: Int J Epidemiol. 2026 Aug 29;55(5):dyag164. doi: 10.1093/ije/dyag164 (PMC13525842; doi:10.1093/ije/dyag164)
Supplement: Supplementary file 1 [file dyag164_supplementary_data.zip › ARF_SynthEpiRep_code.zip!/ARF_SynthEpiRep-main/Supplement/plots/Appx_runtime_results.pdf]

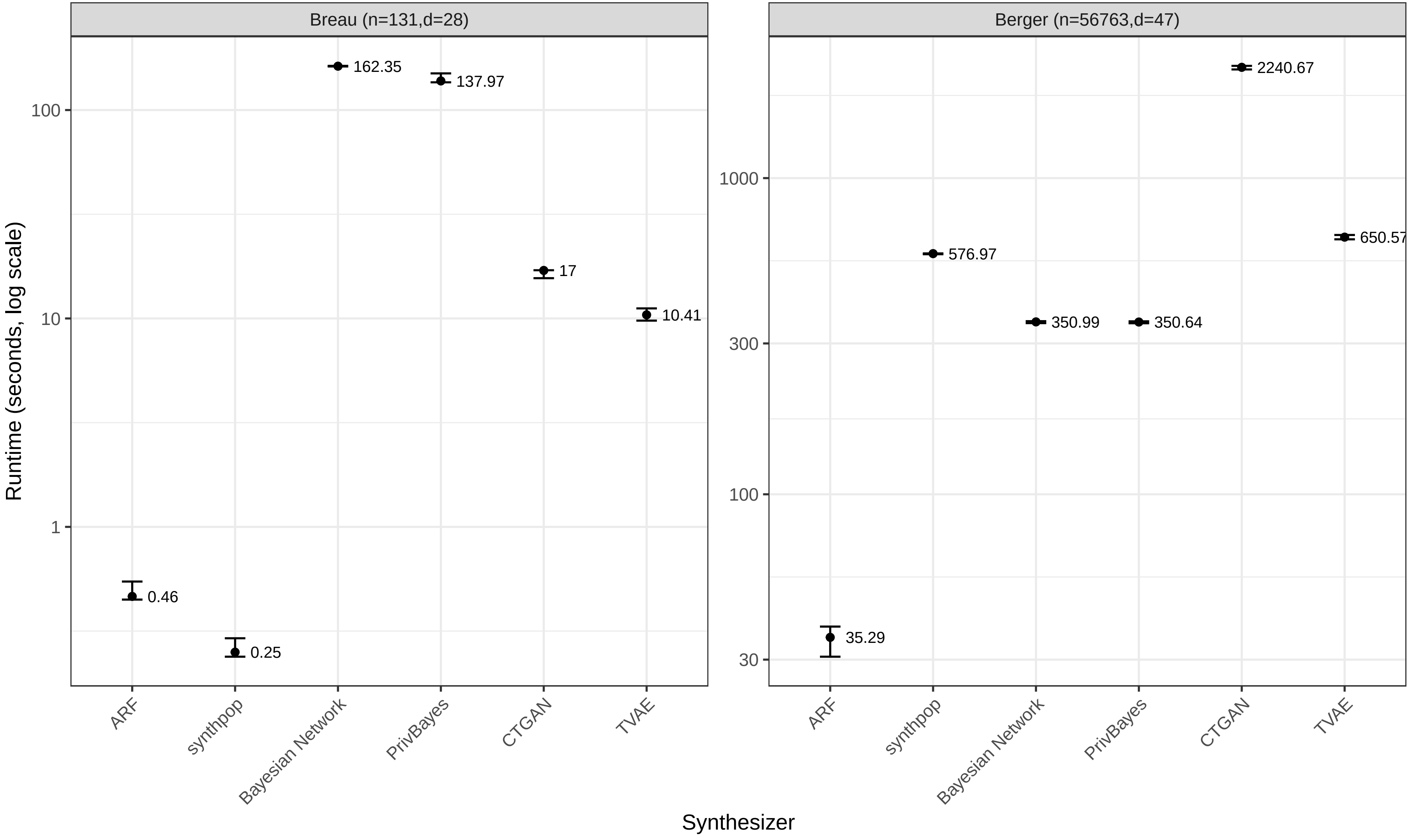

Breau (n=131,d=28)
Berger (n=56763,d=47)
Runtime (seconds, log scale)
100
10
1
1000
300
100
30
162.35
137.97
17
10.41
0.46
0.25
2240.67
650.57
576.97
350.99
350.64
35.29
ARF
synthpop
Bayesian Network
PrivBayes
CTGAN
TVAE
Synthesizer
